# A Reversibly Porous Supramolecular Peptide Framework**

**DOI:** 10.1002/chem.202202368

**Published:** 2022-10-01

**Authors:** Dominic F. Brightwell, Giada Truccolo, Kushal Samanta, Elliott J. Fenn, Simon J. Holder, Helena J. Shepherd, Chris S. Hawes, Aniello Palma

**Affiliations:** ^1^ Supramolecular Interfacial and Synthetic Chemistry Group School of Physical Sciences Ingram Building University of Kent CT2 7NH Canterbury UK; ^2^ School of Chemical and Physical Sciences Lennard-Jones Building Keele University ST5 5BG Staffordshire UK

**Keywords:** enantioselectivity, helical structures, host–guest systems, peptides, polyproline, supramolecular chemistry

## Abstract

The ability to use bio‐inspired building blocks in the assembly of novel supramolecular frameworks is at the forefront of an exciting research field. Herein, we present the first polyproline helix to self‐assemble into a reversibly porous, crystalline, supramolecular peptide framework (SPF). This framework is assembled from a short oligoproline, adopting the polyproline II conformation, driven by hydrogen‐bonding and dispersion interactions. Thermal activation, guest‐induced dynamic porosity and enantioselective guest inclusion have been demonstrated for this novel system. The principles of the self‐assembly associated with this SPF will be used as a blueprint allowing for the further development of helical peptide linkers in the rational design of SPFs and metal‐peptide frameworks.

## Graphical abstract

**The ability to use** bio‐inspired building‐blocks in the assembly of novel supramolecular frameworks is at the forefront of an exciting research field. Herein, we present the first polyproline helix to self‐assemble into a reversibly porous, crystalline, supramolecular peptide framework (SPF). Thermal activation, guest‐induced dynamic porosity and enantioselective guest inclusion have been demonstrated for this novel system.

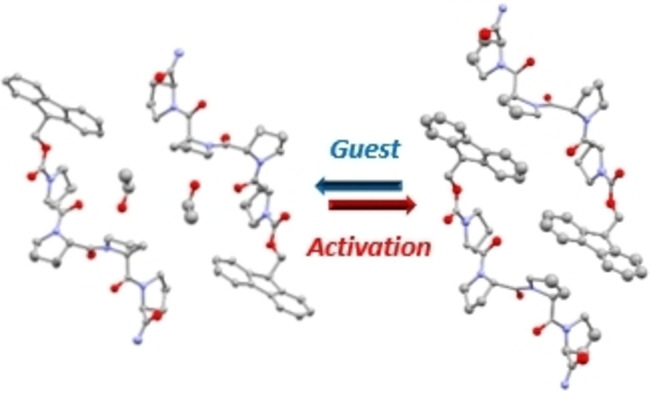

## Introduction

Peptide‐based porous frameworks are an emerging class of materials that have found applications as adaptive[^1^, ^2^, ^3^] and reversibly tuneable^[2]^ porous materials capable of capturing greenhouse gases,[^4^, ^5^, ^6^] and, due to their inherent chirality, have also found applications in facilitating chiral events and transformations,^[7]^ and separating chiral drugs.[^8^, ^9^] Peptides can be prepared at scale with high purity, have canonical and noncanonical amino acids incorporated into their primary structure with high accuracy and are biocompatible. As such, the efforts to investigate this class of compounds as chiral, tuneable ligands have seen a surge in recent years.[^8^, ^9^, ^10^, ^11^, ^12^, ^13^, ^14^] While efforts to post‐functionalise metal–organic frameworks (MOFs) with helical peptides,[^7^, ^15^] to induce chiral selectivity, and to synthesise a broad range of metal‐peptide frameworks (MPFs) have seen considerable advances,[^8^, ^9^, ^10^, ^12^, ^16^] it remains a challenge to develop peptide‐based extended porous networks guided by supramolecular interactions (e. g., H‐bonding, halogen bonding, π‐interactions and host–guest interactions).[^17^, ^18^, ^19^] To the best of our knowledge, the use of cyclic peptides to yield porous channels in crystalline materials and the use of self‐assembling hydrophobic dipeptides, are amongst the few examples of well characterised peptide‐based frameworks (i. e., supramolecular peptide frameworks; SPFs).[^4^, ^6^, ^13^, ^20^, ^21^] In recognition of the immense chemical space yet to be explored in this field, we set out to exploit peptides with stable secondary structures in the construction of SPFs. In particular, we focused our efforts on the use of polyproline helices as SPF building blocks due to the accessibility to rigid yet stimuli sensitive secondary structures.^[22]^ Polyprolines can interconvert between two different secondary conformations, polyproline II with all‐*trans* amide bonds and polyproline I with all‐*cis* amide bonds. This interconversion can be controlled as a function of the environment it is exposed to (i. e., temperature, solvent polarity and pH) and can be monitored by circular dichroism (CD) spectroscopy.[^23^, ^24^, ^25^] Also, polyproline helices have also shown remarkable resilience to a diverse range of functionalisation, in contrast to other helices used in supramolecular chemistry,[^26^, ^27^, ^28^] retaining their conformation even in short sequences.^[29]^ These properties make them promising candidates for the synthesis of novel porous SPFs.

**Figure 1:**
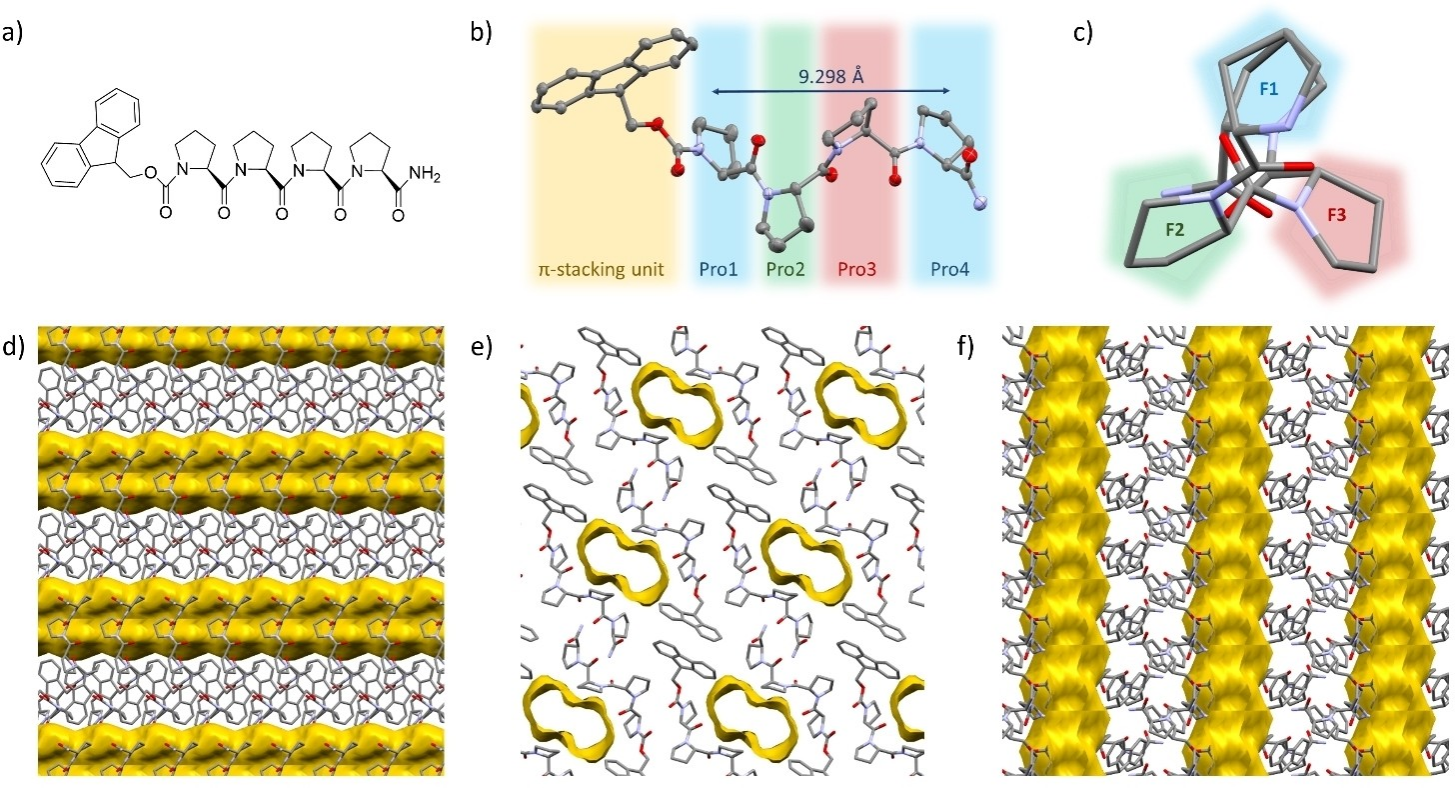
a) Proline tetramer Fmoc‐(Pro)_4_‐NH_2_ (PP_4_), b) Crystal structure of PP_4_(‐SPF), 50 % ellipsoids (Mercury), c) View along the axis displaying symmetry and the three faces (F1–3) of the helix; the Fmoc group has been removed for clarity, d) Crystal structure of PP_4_‐SPF showing packing and solvent accessible voids (yellow) viewed along the *a*‐axis, ethanol molecules were removed, Views along the e) *b*‐ and f) *c*‐axes.

Herein, we report the first reversibly porous SPF formed by the self‐assembly of a helical tetraproline peptide (Figure 1). This supramolecular peptide framework is formed by the self‐assembly of a short helical tetraproline through hydrogen‐bonding and Fmoc–Fmoc interactions. The remarkably resilient SPF is capable of reversibly hosting guest molecules within the channels through the guest‐induced reversion from a collapsed de‐solvated state. The inherently chiral nature of the peptide framework results in enantioselectivity of this adsorption process. Wennemer's group reported the first crystal structure of a polyproline hexamer in the polyproline II conformation providing insight into the stability of the polyproline helix,^[30]^ followed by Hanessian, who reported the crystal structure of the tetrameric proline congener (*cis‐*4,5‐methanoproline) in the polyproline II form.^[31]^ Previously, a crystal structure of a tetrameric oligoproline was reported by Matsuzaki, with several of the proline units deviating from the typical polyproline II conformation.^[32]^ However, reports have suggested that tetraproline can be found in the structured polyproline II helical conformation when in solution.[^29^, ^31^] To the best of our knowledge, tetraproline is the shortest polyproline II helix reported in the literature,[^29^, ^30^, ^31^] therefore we decided to focus our initial efforts on this oligomer in the construction of structured SPFs.

## Results and Discussion

An oligoproline tetramer, PP_4_ was successfully synthesized using solid‐phase peptide methodology in a quantitative yield (Supporting Information 1). The N terminus was capped with an Fmoc carbamate group to facilitate intramolecular assembly of peptides through interactions between the bulky aromatic groups, whereas the C terminus was amidated to act as a hydrogen‐bond donor (H_D_). PP_4_ was characterized in solution with far‐UV circular dichroism (CD) analysis of aqueous, ethanol, and propanol solutions (0.25 mM), showing that the peptide retains the polyproline II helix in all solvents (*λ*_max_ at 223/229 nm and *λ*_min_ at 202 nm; Supporting Information 5, Figure S6).

Crystals of PP_4_ suitable for single‐crystal X‐ray diffraction analysis were reproducibly obtained by slow cooling of a hot super‐saturated solution of PP_4_ in ethanol (Supporting Information 7). The structure was solved and refined to an atomic resolution of 0.81 Å (Figure 1), and confirmed that the peptide adopts the polyproline II helical conformation. The characteristic dihedral angles and an analysis of the puckering of the pyrrolidine rings, have been summarized in Table S1 (Supporting Information 9.2). We found that pyramidalization of the amide carbonyls clearly indicated the presence of n‐π* interactions and these interactions were more significant in prolines exhibiting the *exo* conformation with a larger degree of pyramidalization (Supporting Information 9.3, Table S2).[^30^, ^33^]

Analysis of the packing of PP_4_ showed an extended supramolecular peptide framework (PP_4_‐SPF) with channels extending in one dimension through the network (void volume 226 Å^3^, Supporting Information 9.4), which were occupied with disordered solvent molecules (i. e., ethanol; Supporting Information 9, Figure S8).

**Figure 2:**
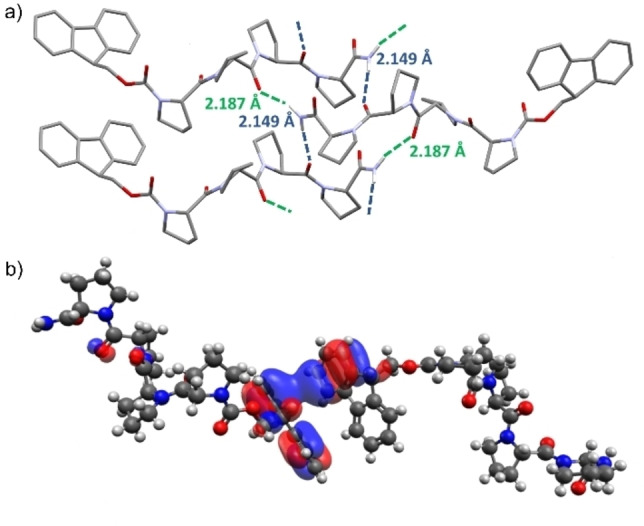
a) Hydrogen bonding between a single peptide unit and two opposing peptide units, through terminal amide hydrogens and Pro2 and Pro3 carbonyls (2.1930(18) and 2.1478(19) Å, respectively), that extends through the framework (PP_4_‐SPF), b) Model of the PP_4_ dimer (PP_4_‐SPF) illustrating orbital interactions between Fmoc groups. For details see Supporting Information 14.1).

The formation of the extended framework is driven by a combination of hydrogen‐bonding and Fmoc‐Fmoc interactions. The peptides are arranged in alternating antiparallel rows, extending through the network (Figure 1d–f). Each row of peptides is offset from the next row such that the C‐terminal amide can hydrogen bond with two other peptides. The C‐terminal amides hydrogen bond with the carbonyl group of Pro2 on the neighbouring peptide 1 and the Pro3 carbonyl on peptide 2 (2.1478(19) Å and 2.1930(18) Å respectively, Figure 2a), therefore each peptide acts as both a H_*D*_ and H_*A*_ with another peptide extending to create 2D sheets, stacked with weaker interlayer interactions in an alternating manner creating an extended network. The crystal structure suggests that interactions between the Fmoc groups determine the second aspect of the self‐assembly process. The closest distance (2.7515(19) Å) between the Fmoc moieties is that of a proton of one fluorenyl to the aromatic region of another (Supporting Information 9, Figure S10), well within the possible distance to classify a potential interaction.[^34^, ^35^, ^36^] It is also worth noting that a reversible single‐crystal to single‐crystal transition in the unit cell of PP_4_‐SPF (Supporting Information 9.1–9.3, PP_4_‐SPF_Flash_) is observed, whereby the Pro3 puckering switches from *exo* to *endo* (Supporting Information 9.3, Table 1). This was achieved by flash freezing crystals at 150 K, remarkably, upon returning to room temperature the crystals readopt their initial unit cell and conformation. This was not found when slowly ramping the temperature down to 150 K, with only a slight reduction of the cell volume, but no significant conformational changes.

Computational calculations at [B3LYP−D3(BJ)/6‐311++G(d,p)] on an Fmoc‐associated pair (geometry fixed to that of the PP_4_‐SPF crystal structure) showed orbital overlap between the groups (Figure 2b and Supporting Information 14, Figure S45). The electrostatic potential map showed relatively electron poor inner regions for the Fmoc groups, as expected, but no obvious points of significant electrostatic interaction between the groups (Supporting Information 14.1, Figure S44). Energy decomposition analysis (EDA) of the isolated dimer system (geometry fixed to that of the PP_4_‐SPF crystal structure) was used to determine the relative breakdown of the intermolecular forces between the Fmoc groups in the crystal lattice. Calculations were run with two different functionals, BLYP−D3(BJ) and PBE−D, giving interaction energies of −44.2 and −32.4 kJ mol^−1^ respectively. The breakdown suggests that dispersion interactions are the predominant interaction between the Fmoc groups with smaller contributions from electrostatic and orbital interactions (and the Pauli repulsive interaction, Supporting Information 14.2, Table S4).

Due to the porosity of the framework, it has the potential for “activation” by removal of the solvent from the pores, thereby allowing the introduction of other guests into the activated pores. To investigate the ability for thermal release of ethanol from the framework channels, ^1^H NMR analysis of PP_4_‐SPF in [D_4_]methanol was carried out after activating the crystals for various timeframes under vacuum at 45 °C. These studies revealed that thermal activation could gradually reduce the ethanol content over time up to a 90 % reduction after 12 h, compared to the initial content present within the framework (Supporting Information 11, Figure S15). Subsequently, simultaneous thermogravimetric analysis (TGA) and differential scanning calorimetry (DSC) of PP_4_‐SPF showed a crystalline melting point with an endothermic peak (150 °C, Supporting Information 6, Figure S7).

**Figure 3:**
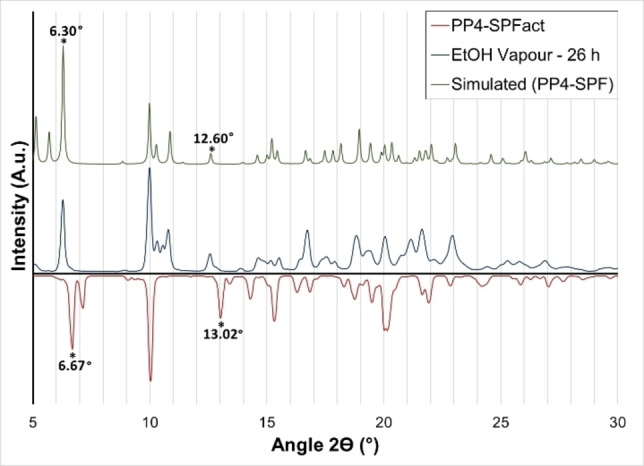
Top: PD‐XRD of PP_4_‐SPF simulated from SC‐XRD data (green). Bottom: PP_4_‐SPF after activation at 45 °C under high vacuum (red) and PP_4_‐SPF_act_ after incubation in a chamber saturated with ethanol vapour for 26 h (blue) showing a clear match with the solvated structure; key peaks indicative of phase change are highlighted.

Powder X‐ray diffraction (PD‐XRD) analyses revealed a change in phase upon de‐solvation, with significant differences to the original sample (Figure 3, Supporting Information 12, Figure S18). Due to these changes SCXRD analysis was carried out on an activated crystal to investigate the change in porosity. Although the crystal showed very poor single crystallinity after the evacuation of the solvent, and no useful reflections were observed beyond 1 Å resolution (Supporting Information 12.1–12.3), the diffraction data could be solved to give a connectivity model (Supporting Information 12.3, Figure S21). This low‐resolution model is presented purely as a comparative connectivity model which suggests collapse of the porous structure with the 2D hydrogen bonded layers shifted relative to one another such that the Fmoc group fills the pore space. However, the predicted powder pattern for the modelled desolvated structure matches very well with the powder pattern of the bulk material (Figure 3, Supporting Information 12.1), thus suggesting the correct assignment of the collapsed structure for the material. Gas absorption studies supported these findings, with negligible gas absorption due to pore collapse upon solvent removal (Supporting Information 12).

Subsequently, the activated sample was then soaked in the ethanol mother liquor to investigate if this phenomenon was reversible. Remarkably, within 15 minutes the crystals showed an almost complete phase change back towards the original structure with a complete return clear after 21 h (Supporting Information 12.2, Figure S20). To rule out the possibility of partial recrystallization causing the phase change, a sample of PP_4_‐SPF_act_ after PD‐XRD analysis, while still on the mount, was placed in a chamber with saturated ethanol vapour for 26 h, before again recording the powder diffraction, which showed a complete change from the activated phase to the original phase (Figure 3, Supporting Information 13.4). This type of behaviour, exhibiting a dynamic porosity whereby the framework is capable of reversible collapse, is more typically seen for MOFs or coordination polymers, whereby the conformational flexibility of a ligand or weak interactions between 2D layers allows for an adaptable transition, dependent on the guest molecules present.[^1^, ^37^, ^38^] However, there have been somewhat similar cases with supramolecular/hydrogen‐bonded frameworks.[^38^, ^39^, ^40^, ^41^] In this case the behaviour results due to the 2D hydrogen‐bonded layers of peptide shifting against one another, behaving as weakly bound 2D interdigitated layers.^[37]^ Thus negligible conformational change of the peptide units occurs and the strong hydrogen bond interactions are retained along two axes (Supporting Information 12.3). This exemplifies the benefits of the exceptional rigidity of the polyproline helix compared to other biomolecules,^[42]^ highlighting its applicability as a structural unit in constructing supramolecular frameworks.

Due to the framework exhibiting dynamic porosity, attempts were made to see if this behaviour would apply with other guests. PP_4_‐SPF_act_ was soaked in hexane and showed no change in phase on the powder pattern, as such hexane was used as the diluent for other guest molecules. The framework was soaked in a 5 % solution of various guests (Table S3, further details Supporting Information 13). After soaking the SPF was washed thoroughly with fresh hexane to remove excess guest. PDXRD analysis was then carried out to determine whether a change in phase had occurred (i. e., reinflation) for each guest (Supporting Information 13.4). The mol % of guest retained within the pores was analysed by ^1^H NMR, after dissolving the framework in [D_4_]methanol (Table S3, Supporting Information 13 and 13.3).

All the guests analysed (acetone, THF, 1‐bromohexane, ethyl acetate, toluene, (±) 1‐phenylethanol, and iodine), elicited a change in phase from the de‐solvated structure, after analysis of the solid SPF after soaking, suggesting they all act to reinflate the structure (Supporting Information 13.4). However, differences in the powder patterns of the samples suggests either a degree adaptation of the framework to accommodate different guests when compared to the original ethanol guest or ordering of the new guest molecules resulting in new diffraction peaks. Although, differences in peak intensities and loss of some low angle peaks are likely attributable to both loss of crystal quality through the processes of activation and guest inclusion. It should be noted that the activated material loses crystallinity when mechanically ground (Supporting Information 12, Figure S18), and so the measured diffraction patterns for the (unground) materials also exhibit some preferred orientation effects. Analysis of the ^1^H NMR data showed significantly higher proportions of hexane than for the SPF soaked alone in hexane (Table S3, Supporting Information 13), which suggests that once porosity is recovered hexane is adsorbed into the framework. However, the proportion of guest alters significantly (e. g., from 5 % *v*/*v*), favouring adsorption of guest molecules over the hexane diluent (Supporting Information 13).

**Figure 4:**
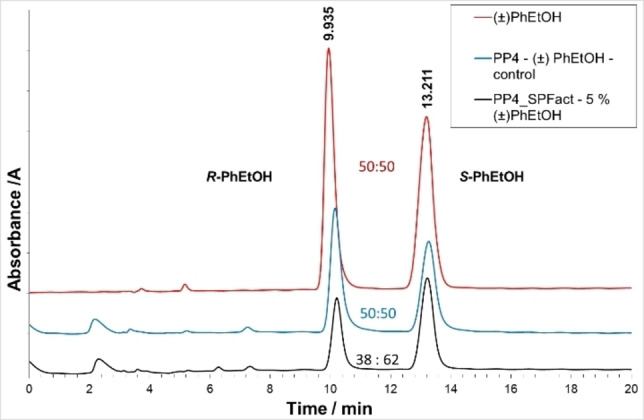
Comparative HPLC chromatogram of a solution of (±) 1‐phenylethanol (red), after incubation of amorphous PP_4_ in (±) 1‐phenylethanol (5 % in hexane; blue), and after extraction from PP_4_‐SPF_act_ after 1.5 h of incubation in (±) 1‐phenylethanol (5 % in hexane; black). The enantioselectivity towards the guest encapsulated within the de‐solvated SPF is highlighted. (For further details, see Supporting Information 13.5).

As the SPF building blocks are inherently chiral, with the specific helicity introduced by the polyproline II conformation, we decided to investigate the framework's ability for enantioselective adsorption. With this in mind, and with the successful encapsulation of the chiral molecule (±) 1‐phenylethanol (PhEtOH) within the framework (<60 mol%, Table S3, Supporting Information 13), chiral HPLC was utilized to investigate whether any enantioselectivity was exhibited (Supporting Information 13.5). These results showed a clear shift in the enantiomeric ratio from the reference racemic (±) PhEtOH after exposure to PP_4_‐SPF (≈24 % *ee*, (*S*)‐1‐phenylethanol) (Figure 4, Supporting Information 13.5).

**Figure 5:**
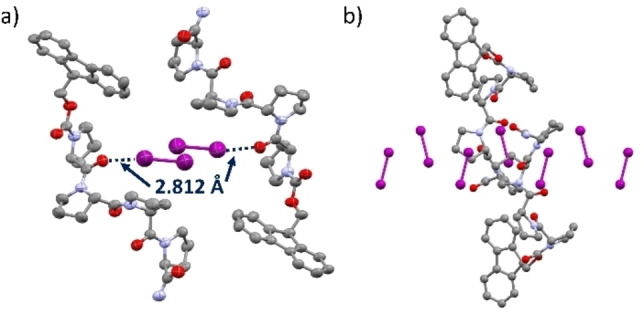
Crystal structures of PP_4_‐SPF@I_2_. a) View along the *b*‐axis, two parallel peptide units’ halogen bond interactions (I⋅⋅⋅O distance of 2.812(16) *2.812* Å) with two molecules of iodine (purple), 50 % ellipsoids (Mercury). b) View along the *a*‐axis showing iodine filling the channels.

Activated single crystals were then exposed to a solution of iodine in hexane to allow visualization of a guest within the reinflated pores through single‐crystal diffraction, even at lower guest loadings, due to the high electron density of iodine. A colour change was evident with the crystals taking on a yellow‐orange hue. SC‐XRD analysis of PP_4_‐SPF@I_2_ was performed which showed that the SPF readopted the initial solvated structural geometry and was able to adsorb iodine with a chemical occupancy for I_2_ of ≈12 % (Figure 5, Supporting Information 13.1). The interatomic distances (2.813(16) Å) between iodine and the Pro1 carbonyl oxygens were indicative of halogen bond formation (Figure 5). To investigate the ability to perform reversible guest absorption with iodine, the same single crystal of PP_4_‐SPF@I_2_ was thermally treated, while mounted on the stage, using a heated flow of N_2_ at 50 °C for 3 h, prior to repeating SC‐XRD analysis. The crystal was mounted without the use of any inert oil, to facilitate guest loss. Upon analysis of the thermally treated crystal, the chemical occupancy of I_2_ was reduced by 50 % while crystallinity of the sample was retained (Supporting Information 13.1), thus demonstrating the potential of the PP_4_‐SPF to perform thermally responsive guest release.

## Conclusion

In conclusion, the first supramolecular peptide framework formed by the self‐assembly of a polyproline helix (PP_4_‐SPF) using the resilience and rigidity of the polyproline II helix in a short oligoproline is reported. This SPF shows remarkable reversible porosity and the ability to reversibly host chemical guests in its channels with exhibited enantioselectivity of this adsorption process. The self‐assembling principles associated with this SPF can be used as a blueprint in the synthesis of novel helical SPFs and have exciting potential for the rational design of functionalized cavities, offering an exceptional level of positional control of easily incorporated new functionalities. These materials have clear applications in chemical separation and potential chiral catalysis.

Deposition Numbers 2127748, 2127749, 2127750, 2127751, and 2156434 contain the supplementary crystallographic data for this paper. These data are provided free of charge by the joint Cambridge Crystallographic Data Centre and Fachinformationszentrum Karlsruhe Access Structures service.

## Conflict of interest

The authors declare no conflict of interest.

1

## Supporting information

As a service to our authors and readers, this journal provides supporting information supplied by the authors. Such materials are peer reviewed and may be re‐organized for online delivery, but are not copy‐edited or typeset. Technical support issues arising from supporting information (other than missing files) should be addressed to the authors.

Supporting Information

## Data Availability

The data that support the findings of this study are available in the supplementary material of this article.
