# Supplementary material for: A Reversibly Porous Supramolecular Peptide Framework
Source: Chemistry. 2022 Oct 1;28(66):e202202368. doi: 10.1002/chem.202202368 (PMC9828346; doi:10.1002/chem.202202368)
Supplement: Supplementary file 1 — Supporting Information [file CHEM-28-0-s001.pdf]

Chemistry–A European Journal

Supporting Information

A Reversibly Porous Supramolecular Peptide Framework

Dominic F. Brightwell, Giada Truccolo, Kushal Samanta, Elliott J. Fenn, Simon J. Holder, Helena J. Shepherd, Chris S. Hawes, and Aniello Palma*

Table of Contents

SI 1. Synthesis of Fmoc-Pro_4-NH_2 (PP_4): 2

SI 2. Chromatography and Mass spectrometry data 3

SI 3. NMR Experiments: 5

SI 4. FT-IR Analysis of PP_4: 7

SI 5. Circular Dichroism Spectroscopy of PP_4 8

SI 6. Simultaneous thermogravimetric analysis of PP_4-SPF 8

SI 7. Crystallisation of PP_4-SPF 9

SI 8. X-Ray Single Crystal Structure Analysis 9

SI 9. Crystal Structure Data from PP_4-SPF SC-XRD analysis 10

SI 9.1 Crystal data and structure refinement for PP_4-SPF 12

SI 9.2. Proline ring puckering and helix parameters 12

SI 9.3. PP_4 n-π* interactions 13

SI 9.4. Modelling of PP_4-SPF pores 14

SI 10. PD-XRD of PP_4-SPF 15

SI 11. NMR Studies to determine thermal activation potential 16

SI 12. Gas Adsorption Studies 18

SI 12.1 Single Crystal X-ray Diffraction of the Desolvated Phase (PP_4-SPF_{act}) 20

SI 12.2 Re-solvation Experiments 21

SI 12.3 Crystal structure of PP_4-SPF_{Act} 22

SI 13. Framework Host-Guest chemistry: 24

SI 13.1 Crystal Structure of PP_4-SPF@I_2 25

SI 13.2 Crystal data and structure refinement for PP_4-SPF@I_2 and PP_4-SPF@I_{2_Heated} 26

SI 13.3 ^{1}H NMR studies of SPF after guest encapsulation 27

SI 13.4 Powder Diffraction data after SPF_{act} guest soaking 36

SI 13.5 Enantioselectivity studies of PP_4-SPF_{act} for (±) 1-Phenylethanol 42

SI 14. Computational Methods 43

SI 14.1 - Molecular Orbital and Electrostatic Potential Modelling 43

SI 14.2 Energy Decomposition Analysis 48

Acknowledgements 49

References 49

SI 1. Synthesis of Fmoc-Pro$_4$-NH$_2$ (PP$_4$):

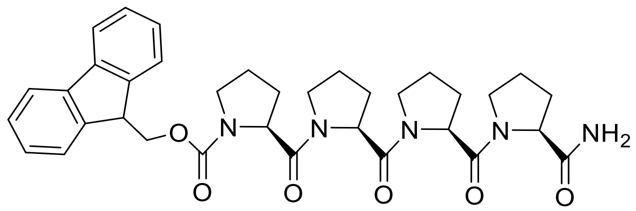

PP$_4$

Materials *– Rink Amide MBHA resin (100-200 mesh, 0.3 mmol/g) 1% DVB, Fmoc-L-amino acids, N,N-Diisopropylethylamine (DIPEA) and benzotriazol-1-yl-oxytripyrrolidinophosphonium hexafluorophosphate (PyBOP) were obtained from Fluorochem Ltd (Derbyshire, UK).*

Deprotection – Initial deprotection: A solution of DMF/Piperidine (20 %) (5 ml) was added to the resin (1 g, 0.3 mmol), pre-swollen in DCM for 20 mins and washed with DMF (4 ml x 4), and agitated for 15 mins, before repeating and washing with DMF (4 ml x 4). Further deprotections: A solution of DMF/Piperidine (20 %) (4 ml) was added to the resin and agitated for 15 mins, before repeating and washing with DMF (4 ml x 4).

Coupling Reaction - A solution of PyBOP (4 eq, 624.5 mg, 1.2 mmol), DIPEA (4 eq, 210 µL, 1.2 mmol) and Fmoc-Pro-OH (2 eq, 202.4 mg, 0.6 mmol) in DMF (3.5 ml) was added to the resin and agitated for 20 mins before repeating and washing with DMF (4 ml x 4).

Peptide Synthesiser Method – Synthesised on a 0.1 mmol scale on Rink amide MBHA resin using a Liberty™ microwave peptide synthesiser (CEM) utilising Fmoc solid-phase peptide synthesis techniques and repeated steps of single deprotections, and couplings interspaced with washings (4 x 4 ml DMF). The synthesis was paused after the final coupling step and the resin removed from the reaction vessel before stopping the synthesis (to prevent gradual loss of the Fmoc group). Deprotection: 20 % piperidine in DMF (4.5 ml) for 5 min with 30 W microwave irradiation at 90 °C. Coupling: Fmoc-Pro-OH (1.5 ml, 0.2 M, 3 eq.), DIC (1.2 ml, 0.5 M, 6 eq.), Oxyma Pure (0.6 ml, 0.5 M, 3 eq.) in DMF, and DMF (3 ml) for 5 min at 90 °C with 30 W microwave irradiation.

Peptide Cleavage - The resin was then washed with DCM (x 4), before the Fmoc-protected peptide was cleaved from the resin with TFA (95 % in DCM) for 1.5 h. The resin was then washed with the cleavage cocktail (x 2) and the filtrate was concentrated under reduced pressure before precipitation in cold Et_2O and centrifugation. The solution was then decanted and the solid repeatedly washed with cold Et_2O to isolate Fmoc-(Pro)$_4$-NH$_2$, **PP$_4$**, as a white solid in a quantitative yield after drying under vacuum. **PP$_4$** was then used without any further purification, < 99 % purity by analytical reverse-phase HPLC. Crystalline samples were crystallised from a hot EtOH solution of **PP$_4$** (≈25 mgml^{-1}) (see **SI 7** for full crystallisation conditions).

PP$_4$, **^{1}H NMR** (400 MHz, MeOD) δ 7.85 (dd, *J* = 14.4, 7.3 Hz, 2H), 7.70 – 7.58 (m, 2H), 7.48 – 7.31 (m, 4H), 4.76 – 4.68 (m, 1H), 4.66 – 4.61 (m, 0.5H), 4.58 (dd, *J* = 9.0, 4.1 Hz, 0.5H), 4.46 – 4.36 (m, 2H), 4.34 – 4.25 (m, 1H), 4.19 (t, *J* = 4.6 Hz, 0.5H), 4.08 (dd, *J* = 8.7, 3.2 Hz, 0.5H), 3.87 – 3.78 (m, 1H), 3.78 – 3.70 (m, 1H), 3.63 (q, *J* = 7.0 Hz, 4H), 3.59 – 3.52 (m, 1H), 3.51 – 3.42 (m, 1H), 3.42 – 3.35 (m, 1H), 2.32 – 1.74 (m, 17H). **^{13}C NMR** (101 MHz, MeOD) δ = 177.0, 172.8, 172.6, 172.3, 172.1, 156.6, 156.2, 145.4, 142.6, 128.8, 128.2, 126.2, 125.7, 120.9, 68.7, 67.0, 61.2, 59.6, 59.3, 59.1, 58.3, 48.3, 48.0, 47.8, 30.70, 30.0, 29.1, 28.8, 25.8, 25.2, 24.0, 18.4. m/z calcd. FT-IR = ν_{max}/cm^{-1} 2956.88 (C-H), 2881.65 (C-H),

1683.86 (C=Os), 1624.06 (C=Os). for $[M+H]^+$ $C_{35}H_{42}N_5O_6^+$: 628.313; found: $[M+H]^+$ 628.3137, $[M+Na]^+$ 650.2938, $[2M+Na]^+$ 1277.6010

SI 2. Chromatography and Mass spectrometry data

HP-LC - Purity determined by analytical HPLC using UV detection at 225 nm: >99 %, on a Kromasil 100-5-C18, 4.6 x 250 mm, 5 to 100 % B over 14.5 mins, R_t = 12.47 min. A is 0.1 % formic acid/water and B is 0.1 % formic acid/methanol.

High-resolution Mass-Spectrometry (ESI+) - Separated on a Phenomenex 2.1 x 150 mm, 3.6 um, XB-C18 Aeris Widepore column at 50 °C on a Waters H-Class Acquity UPLC. R_t = 10.49 mins. Using a 0.1% formic acid/acetonitrile gradient: 5% B for 2.55 minutes then from 5% B to 95% B over 15 minutes, and held at 95% B for 2 minutes. A is 0.1% formic/Water, B is 0.1% formic/Acetonitrile. Flow rate is 0.25 ml/min. The flow is directed into the electrospray source of a Waters G2-Si mass spectrometer, operating in positive ion mode, at 2.5 kV and mass spectra recorded from 100-3000 m/z. Data was analysed with Waters Mass Lynx software. m/z calcd. for $[M+H]^+$ $C_{35}H_{42}N_5O_6^+$: 628.313; found: $[M+H]^+$ 628.3137, $[M+Na]^+$ 650.2938 $[2M+Na]^+$ 1277.6010

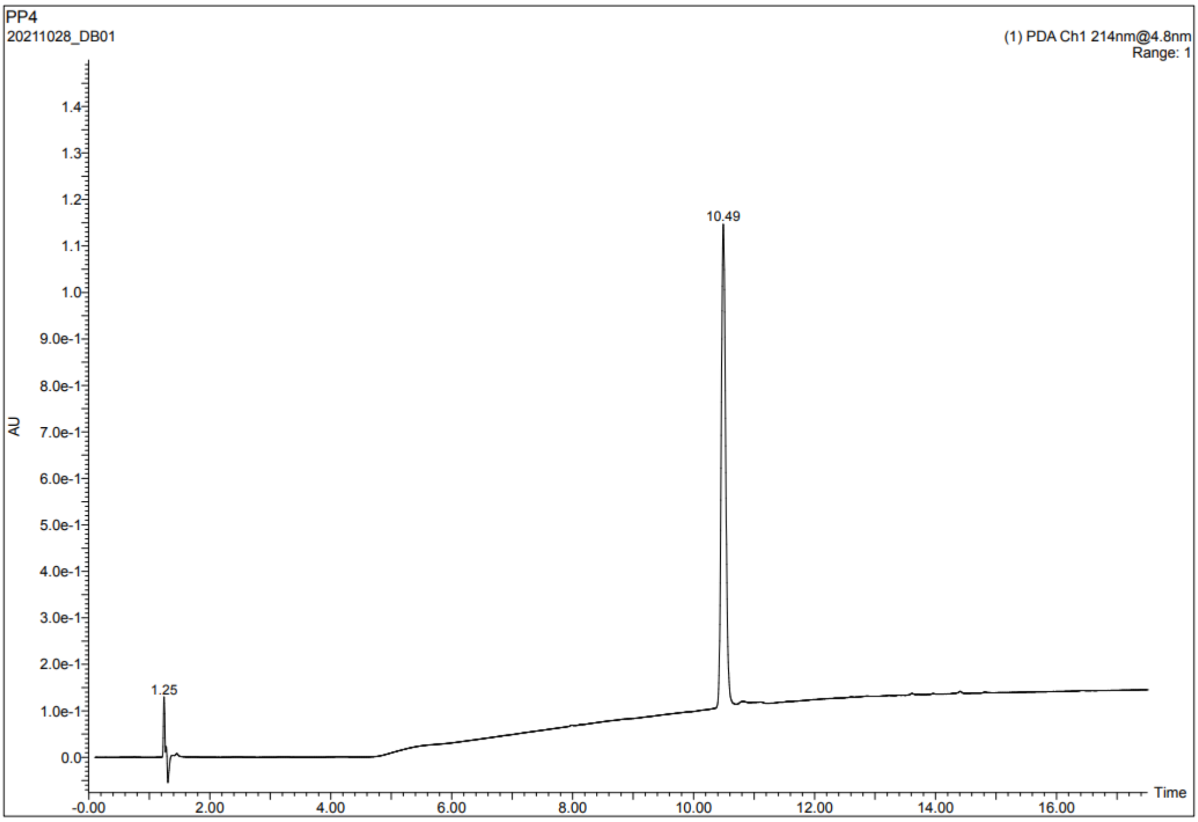

Figure S1 – Analytical UPLC chromatogram from HR-MS analysis of $\mathbf{PP_4}$ (R_t = 10.49 mins)

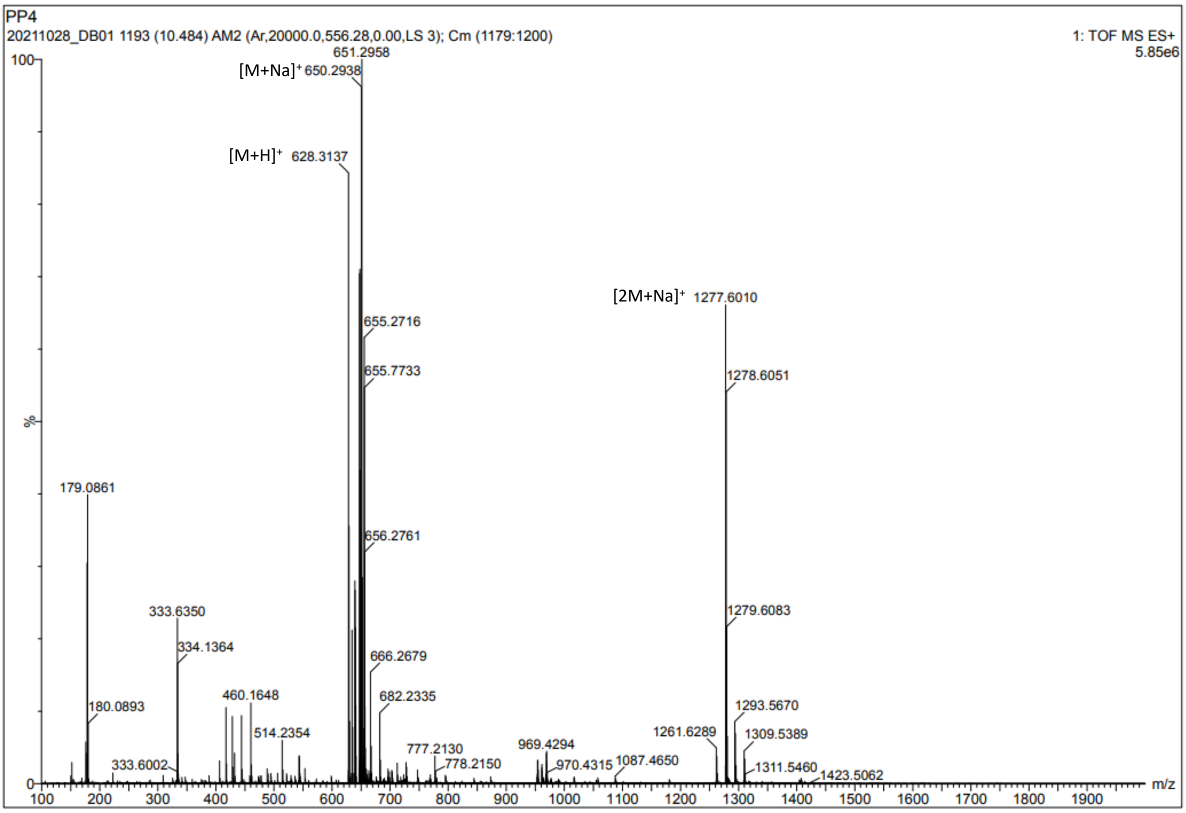

Figure S2 – Mass spectrum (R_t = 10.49 mins) from HRMS analysis of **PP_4**

SI 3. NMR Experiments:

NMR experiments were recorded on a Bruker Avance II 400 MHz spectrometer in MeOD.

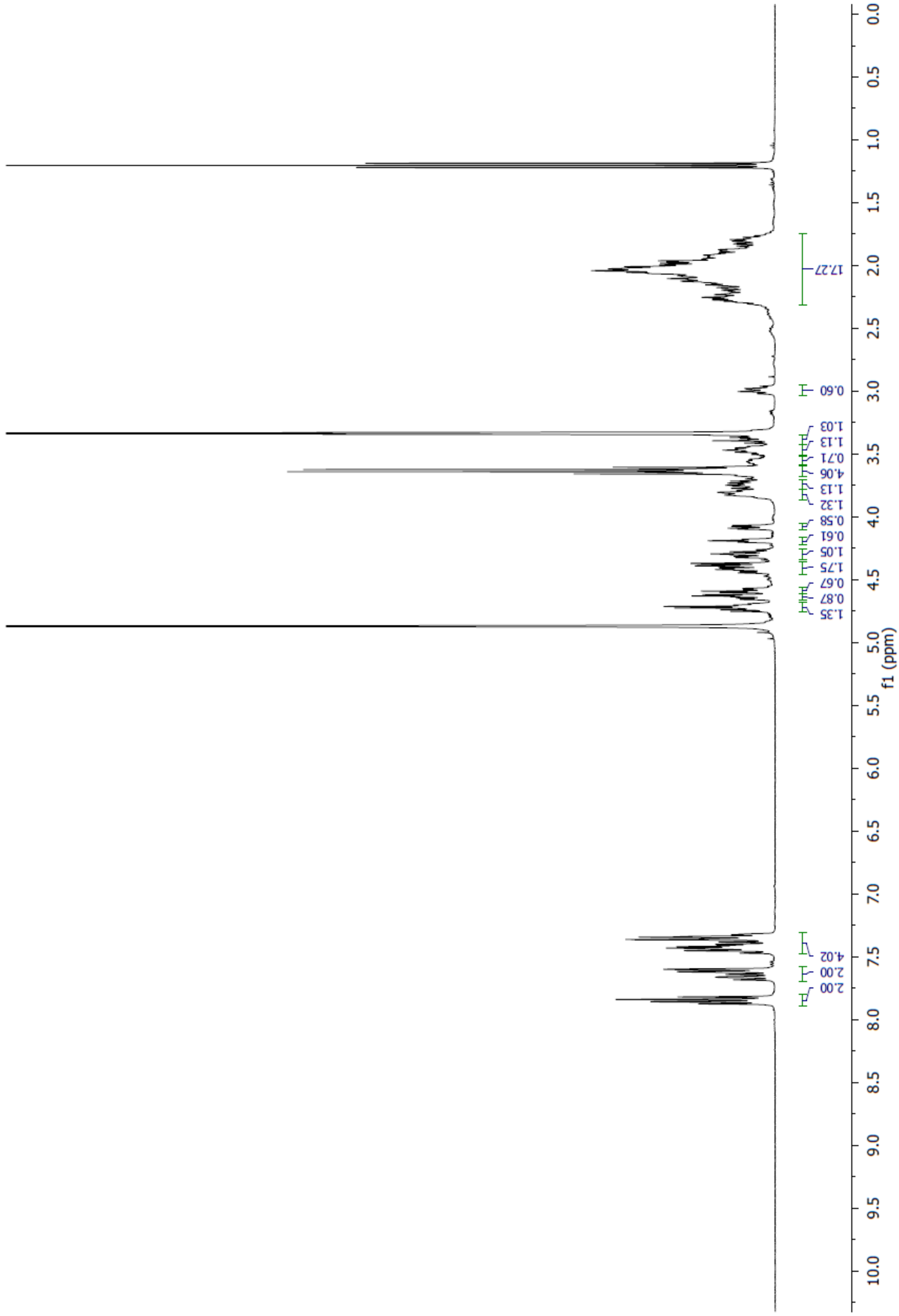

Figure S3 - ^{1}H NMR of **PP_4 (400 MHz, MeOD)**

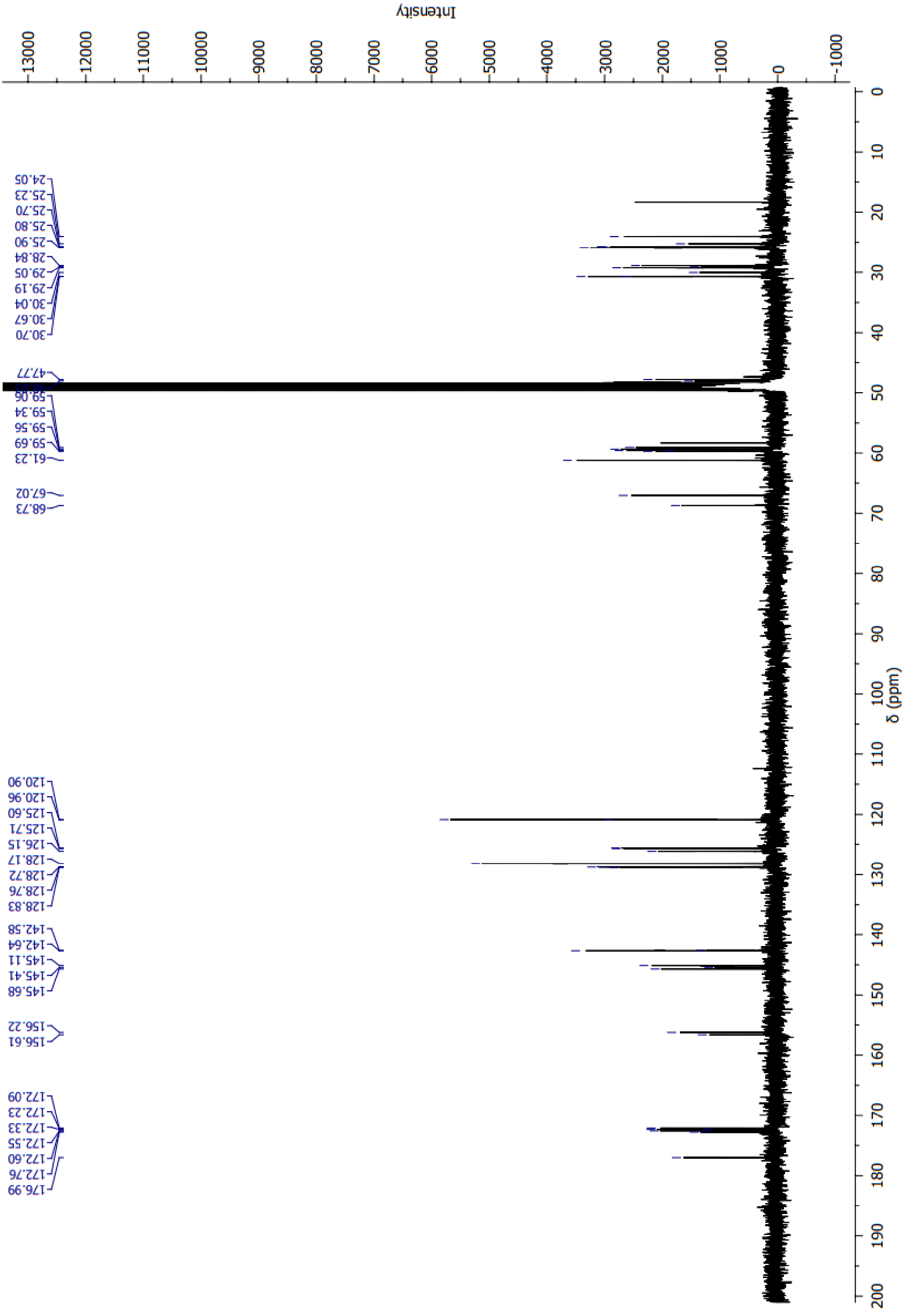

Figure S4 - ^{13}C NMR of **PP_4 (101 MHz, MeOD)**

SI 4. FT-IR Analysis of PP_4:

FT-IR spectroscopy was carried out on a Shimadzu IR Affinity 1S spectrophotometer on $\mathbf{PP_4}$ as a dry solid. The C=O stretching band frequency was typical for that of a PPII helix at 1624 cm^{-1}.[1]

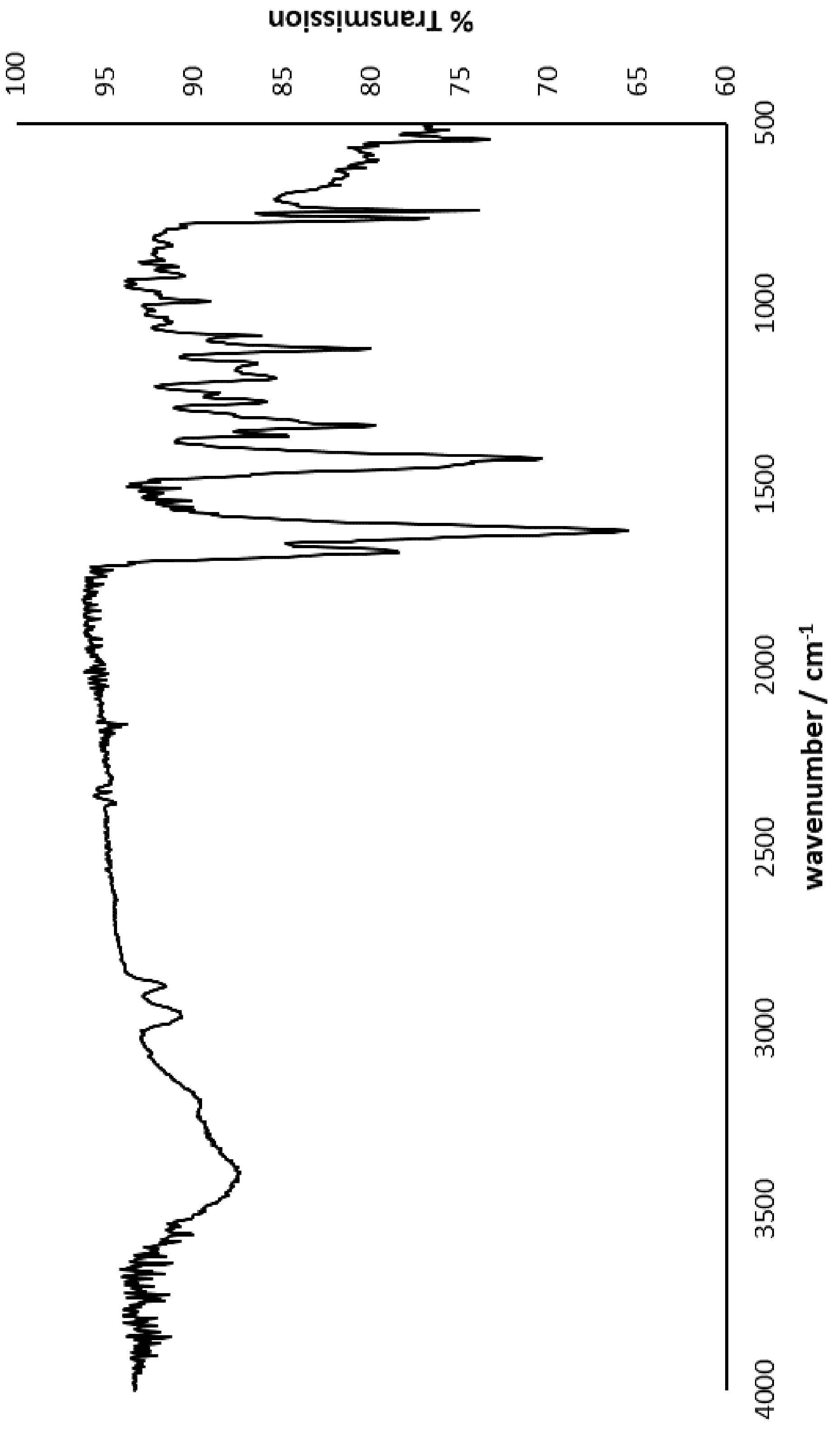

Figure S5 – FT-IR Spectrum of $\mathbf{PP_4}$

SI 5. Circular Dichroism Spectroscopy of PP_4

CD experiments were carried out on a Jasco J-715 spectropolarimeter. Spectra were recorded using a spectral bandwidth of 190-260 nm, at 20 °C, with a scan rate of 100 nm/min. CD data are given in ellipticity (mdeg). The spectra are formed of 4 accumulations and a spectrum of the solvent blank was subtracted from the raw CD data. A Quartz cell was used with a 1 mm path length using 0.25 mM peptide solutions. All samples were kept in solution for 14 days prior to recording CD spectra to ensure final stable conformation has been achieved due to slow conversion between polyproline helices (i.e. Polyproline II→ Polyproline I).

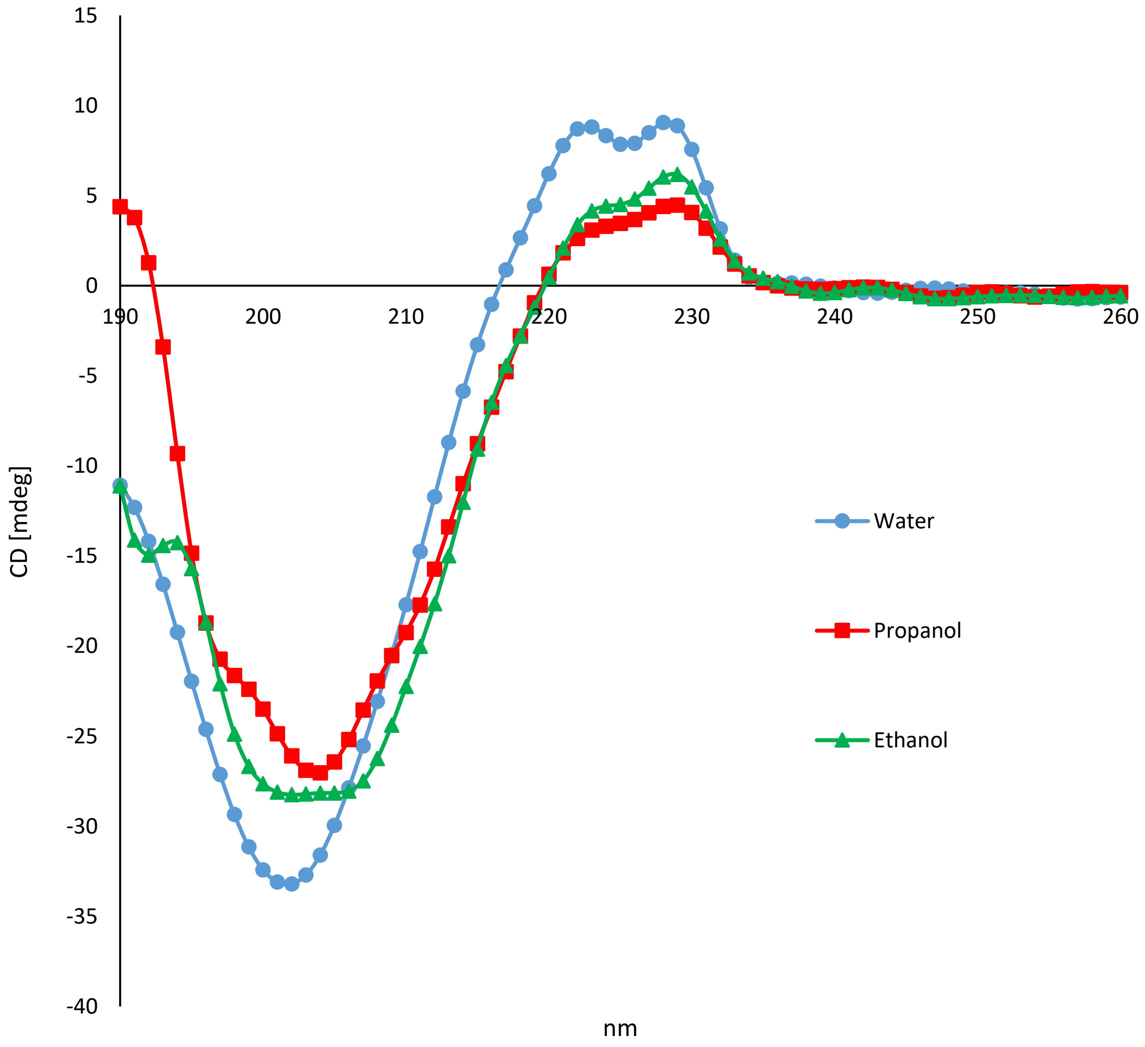

Figure S6 – CD Spectrum overlay of **PP_4** in water, ethanol, and propan-1-ol (0.25 mM) after incubating for 14 days

SI 6. Simultaneous thermogravimetric analysis of PP_4-SPF:

Thermogravimetric analysis was carried out on a STA 409 PC Luxx Simultaneous thermal analyser with N_2 as the purge gas and a temperature range of 30-600 °C and a ramp rate of 10 K/min. A powdered sample of the crystalline **PP_4-SPF** was packed into an aluminium crucible after drying excess ethanol under vacuum at 20 °C for 45 mins and then grinding.

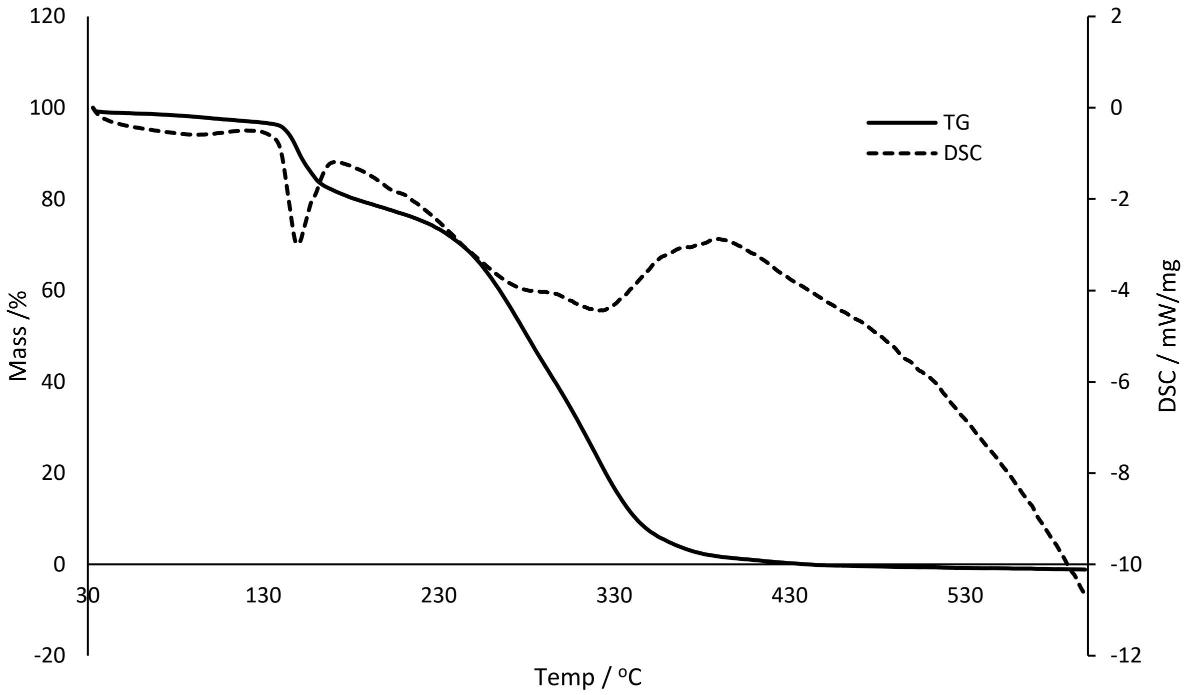

Figure S7 – TGA and DSC trace of **PP$_4$-SPF** showing a crystalline melting point on the DSC trace at 150 °C, mass lost during melting, 12.4 %. Mass loss is likely due to the release of encapsulated solvent upon melting.

SI 7. Crystallisation of PP$_4$-SPF

Crystallisation conditions: Peptide **PP$_4$** was fully dissolved (≈25-30 mgml^{-1}) in hot EtOH before slowly cooling the solution from 60+ °C overnight, forming crystals (colourless planks) in solution. Slow evaporation of the EtOH solution elicits crystal formation on the walls of the vial.

All samples were stable outside of the mother liquor and no significant degradation occurred after air drying (stable at rt out of solution for ≈2-3 months before crystal deterioration).

Iodine adsorption: Single crystals of **PP$_4$-SPF** were heated at 45 °C for 16 hours before soaking in a solution of iodine in hexane for 1 week. A colour change of colourless to orange/yellow crystals was observed over time (*further details* SI13.1).

SI 8. X-Ray Single Crystal Structure Analysis

Single crystal XRD data for the solvated structure and iodine treated sample were collected on a Rigaku Oxford Diffraction SuperNova A S2 single crystal diffractometer using Cu radiation. Sample specific details can be found in the CIF files.

Using the software Olex2,[2] the structures were solved with the ShelXT structure solution program using intrinsic phasing and refined with the ShelXL refinement package using least squares minimization.[3],[4],[5]

CCDC-2127748-2127751, and CCDC-2156434 contain the supplementary crystallographic data for this paper, including structure factors and refinement instructions, and can be obtained free of charge from The Cambridge Crystallographic Data Centre, 12 Union Road, Cambridge CB2 1EZ, UK (e-mail: depos-it@ccdc.cam.ac.uk), or via https://www.ccdc.cam.ac.uk/getstructures.

SI 9. Crystal Structure Data from PP_4-SPF SC-XRD analysis

The crystal structure data was obtained from colorless crystals, crystallised by dissolving $\mathbf{PP_4}$ in hot EtOH (60 °C, ≈25 $mgml^{-1}$) and allowing to slowly cool to room temperature forming crystals rapidly overnight within the solution. The crystals were stable outside of solution at room temperature showing no signs of deterioration over the timeframe of the experiment. For the $\mathbf{PP_4}$**-SPF** crystal, in order to model the disordered solvent, the ethanol molecule was split into two components with a total occupancy of 1. The distances between the oxygen-carbon and carbon-carbon atoms were set to the expected values of 1.43 Å and 1.51 Å respectively. Upon refinement, these distances were then fixed to the same value between the two components and the anisotropic displacement parameters were set to be equivalent within the molecule. For the $\mathbf{PP_4}$**-SPF**$_{\mathbf{\_Flash}}$ the same restraints and constraints were applied as above, with the two components of the ethanol molecule set to 0.75 and 0.25. The oxygen atom of the most abundant component was further split into two, and the total occupancy for its two components was set to the sum of 0.75. A further restraint between the CH_3- carbon and the oxygen atom was necessary to model this component, whose distance was set to be 2.4 Å.

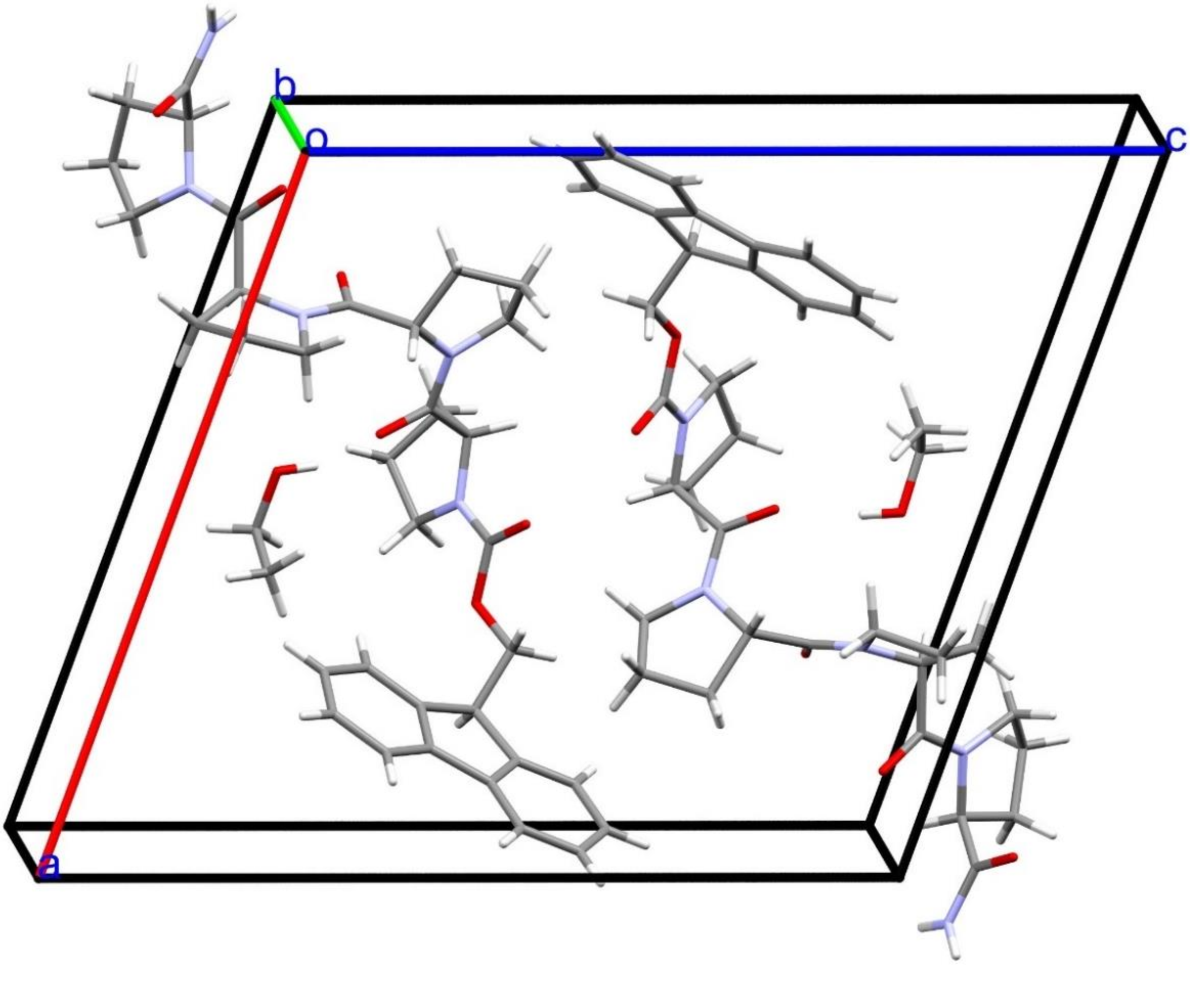

Figure S8 – Unit cell of $\mathbf{PP_4}$**-SPF** showing all molecules with centroids within the cell, *a* axis (red), *b* axis (green), *c* axis (blue), short contacts (2.7933(15) Å) between both peptides between the Cβ hydrogen, on Pro3 to the centroid of the opposing peptide's C24-29 aromatic ring in the Fluorenyl moiety

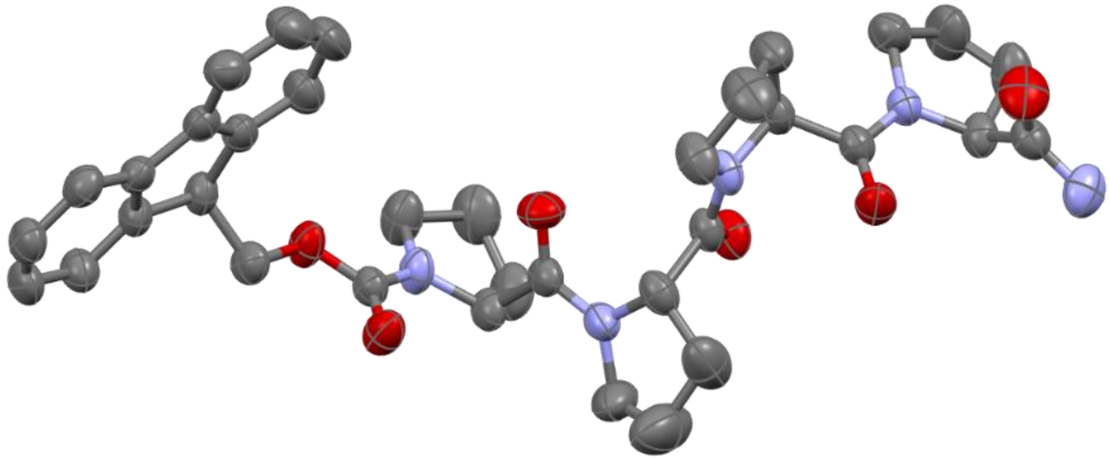

Figure S9 – Crystal structure of **PP$_4$-SPF** showing the asymmetric unit. Atomic displacement parameters are drawn at 50 % probability. Hydrogen atoms have been removed for clarity.

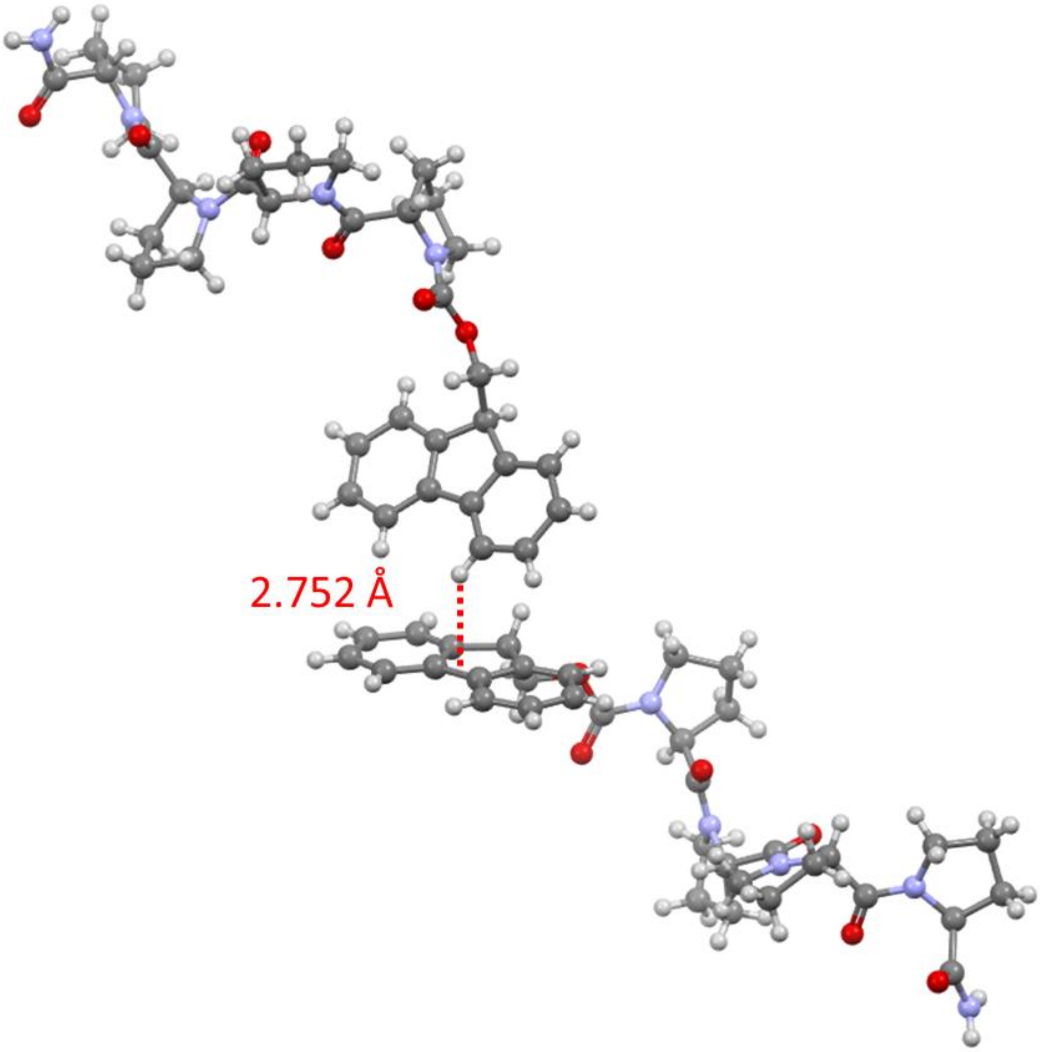

Figure S10 – **PP$_4$-SPF** crystal structure showing the short contact (*dashed red line*, 2.7515(19) Å) between a Fmoc proton on one peptide to the centroid of the aromatic region of a neighbouring fluorenyl moiety

SI 9.1 Crystal data and structure refinement for PP_4-SPF

PP_4-SPF was slowly cooled from 290 K to 150 K with a ramp rate of 280 K/h, while **PP_4-SPF$_{Flash}$** placed on the mount at 150 K (flash frozen). Upon returning to room temperature **PP_4-SPF$_{Flash}$** crystals readopt their initial unit cell and conformation. This was not found when slowly ramping the temperature down to 150 K, with only a slight reduction of the cell volume, but no significant conformational changes.

Identification code	**PP_4-SPF**	**PP_4-SPF$_{_Flash}$**
Empirical formula	$C_{37}H_{47}N_5O_7$	$C_{37}H_{41}N_5O_7$
Formula weight	673.79	667.75
Temperature/K	150(2)	150(2)
Crystal system	monoclinic	monoclinic
Space group	$P2_1$	$P2_1$
a/Å	16.4182(3)	15.5793(2)
b/Å	6.27740(10)	6.34080(10)
c/Å	18.2112(3)	18.5772(3)
α/°	90	90
β/°	109.152(2)	107.249(2)
γ/°	90	90
Volume/Å^3	1773.03(6)	1752.62(5)
Z	2	2
ρ_{calc}g/cm^3	1.262	1.265
μ/mm^{-1}	0.716	0.724
F(000)	720.0	708.0
Crystal size/mm^3	0.217 × 0.063 × 0.054	0.298 × 0.056 × 0.042
Radiation	Cu Kα (λ = 1.54184)	Cu Kα (λ = 1.54184)
2Θ range for data collection/°	8.84 to 146.496	9.972 to 144.02
Index ranges	$-16 \le h \le 20$, $-7 \le k \le 7$, $-22 \le l \le 19$	$-19 \le h \le 18$, $-7 \le k \le 7$, $-22 \le l \le 22$
Reflections collected	20137	19739
Independent reflections	6927 [R_{int} = 0.0225, R_{sigma} = 0.0217]	6762 [R_{int} = 0.0233, R_{sigma} = 0.0225]
Data/restraints/parameters	6927/8/444	6762/9/452
Goodness-of-fit on F^2	1.040	1.055
Final R indexes [I>=2σ (I)]	R_1 = 0.0383, wR_2 = 0.1041	R_1 = 0.0449, wR_2 = 0.1271
Final R indexes [all data]	R_1 = 0.0397, wR_2 = 0.1055	R_1 = 0.0464, wR_2 = 0.1295
Largest diff. Peak/hole / e Å^{-3}	0.56/-0.55	0.67/-0.36
Flack parameter	0.01(5)	0.04(5)

SI 9.2. Proline ring puckering and helix parameters

Proline is known to exhibit two different forms of ring puckering; *exo* and *endo* (Figure S12*),*[6] As such the ring puckering conformations of each proline residue have been assigned, along with the main chain dihedral angles (Table S1).

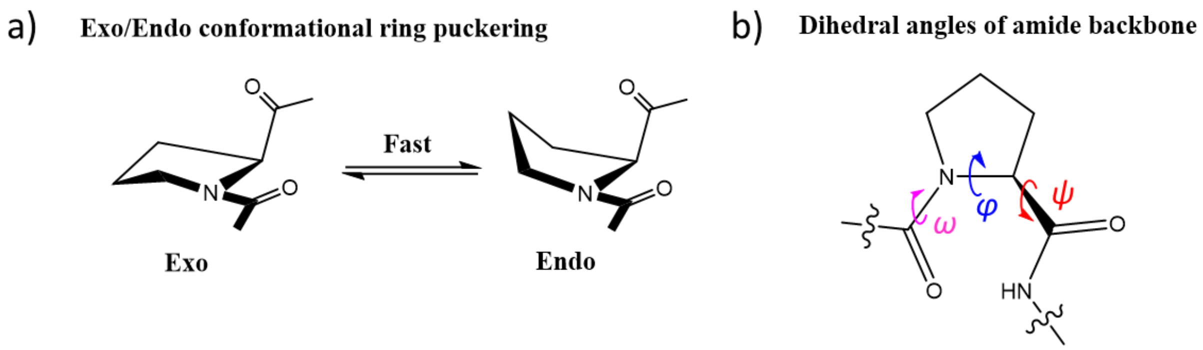

Figure S11 – a) diagram of conformational change due to endo/exo puckering of a proline ring, b) diagram showing the dihedral angles of a proline residue

Table S1. Helix dihedral angles and ring puckering of peptide 1 from PP_4-SPF and PP_4-SPF_{Flash} SC-XRD data

Residue	ω/ °		φ/°		ψ / °		Ring Pucker	
	SPF	SPF_{Flash}	SPF	SPF_{Flash}	SPF	SPF_{Flash}	SPF	SPF_{Flash}
**Pro1**	179.8(2)	-175.4(2)	-77.3(3)	-74.7(3)	154.5(2)	155.5(2)	C^{β}-exo/C^{γ}-endo twist	C^{β}-exo/C^{γ}-endo twist
**Pro2**	179.5(2)	-179.2(2)	-65.4(3)	-75.6(3)	140.8(2)	154.2(2)	**C^{γ}-exo envelope**	**C^{β}-exo/C^{γ}-endo twist**
**Pro3**	174.8(2)	174.1(2)	-70.8(3)	-69.6(3)	163.5(2)	163.2(2)	C^{β}-exo/C^{γ}-endo twist	C^{β}-exo/C^{γ}-endo twist
**Pro4**	-173.3(2)	-174.4(2)	-65.1(3)	-62.1(3)	151.2(2)	146.8(3)	C^{γ}-exo envelope	C^{γ}-exo envelope

SI 9.3. PP_4 n-π* interactions

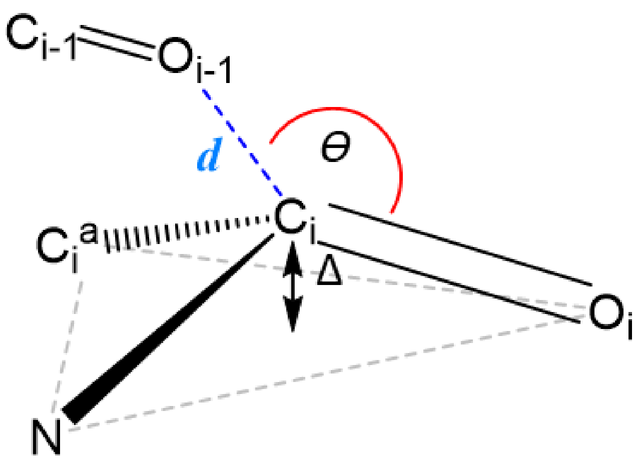

Figure S12 – Diagram depicting the Bürgi-Dunitz trajectory, angle (ϴ) and pyramidalization (Δ), of the *n-π** interaction between adjacent carbonyl groups.

**Table S2. Trajectory angles, distances and pyramidalization of PP_4 carbonyls from PP_4-SPF and PP_4-SPF_{Flash} SC-XRD data**						
**Residue**	**θ_{BD} / °**		***d* / Å**		**Δ / Å**	
	**SPF**	**SPF_{Flash}**	**SPF**	**SPF_{Flash}**	**SPF**	**SPF_{Flash}**
**Pro1**	98.7(2)	95.4(2)	3.204(3)	3.155(3)	0.019	0.018
**Pro2**	103.1(2)	**98.2(2)**	2.937(3)	**3.120(3)**	0.025	**0.018**
**Pro3**	86.9(2)	86.6(2)	3.059(3)	3.084(3)	0.004	0.002
**Pro4**	94.0(2)	95.3(2)	2.964(3)	2.912(3)	0.031	0.026

The polyproline II helix is stabilised by *n-π** interactions, between the carbonyl non-bonding orbitals to the next residue's carbonyl antibonding orbital following the Bürgi-Dunitz trajectory, this effect causes a degree of pyramidalization of the typically planar carbonyl (Figure S13). The greater the degree of pyramidalization and the closer to the typical Bürgi-Dunitz angle and distance, the greater the strength of the *n-π** interaction is likely to be. Analysis of these interactions in the crystal structure data (Table S2) shows that the transition of Pro2 from *exo* to *endo*, exhibited in the flash frozen (150 K) sample (**PP_4-SPF_{Flash}**), resulted in a reduction in pyramidalization by 28 % (0.025-0.018 Å), which supports the theory that the endo conformation disfavours n-π* interactions when combined with the weaker pyramidalization exhibited for all *endo* prolines,[7] compared to those in the *exo* conformation.

SI 9.4. Modelling of PP_4-SPF pores

The void space for **PP_4-SPF** was calculated with Mercury (Probe radius, 1.2 Å, grid spacing, 0.7 Å; Volume 226.19 $Å^3$, 12.8 % of unit cell). The channel is viewed along the *b* axis and is depicted in yellow. The channel is anisotropic, so both the shortest and longest edge to edge distances were measured (shortest, 4.8 x 7.7 Å; largest, 5.0 x 11.7 Å).

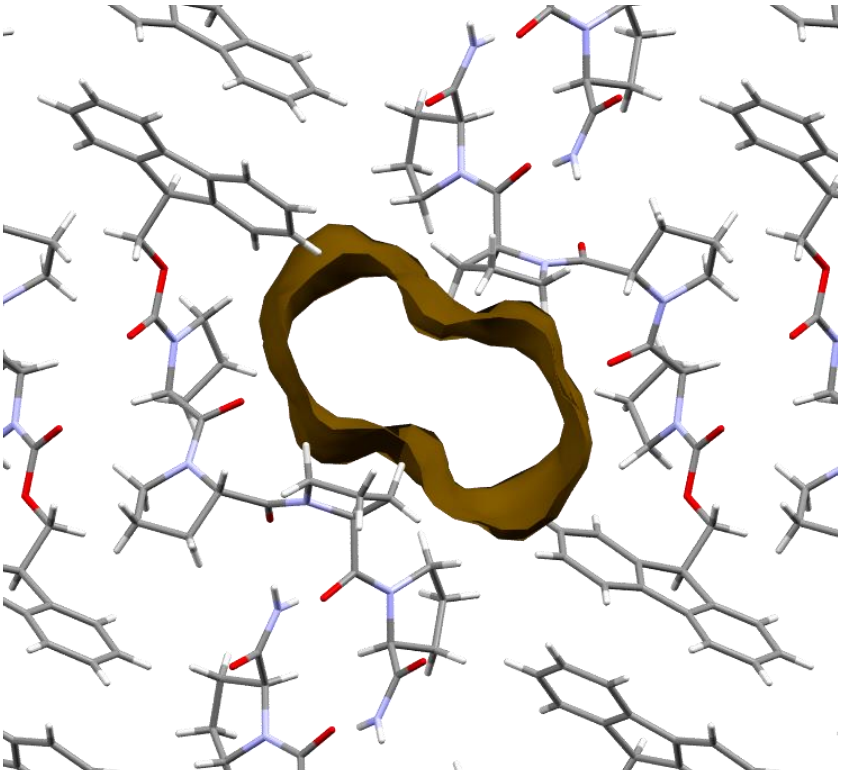

Figure S13 – Model of the channel (yellow) within the crystal structure of **PP$_4$-SPF** (Mercury)

SI 10. PD-XRD of PP$_4$-SPF

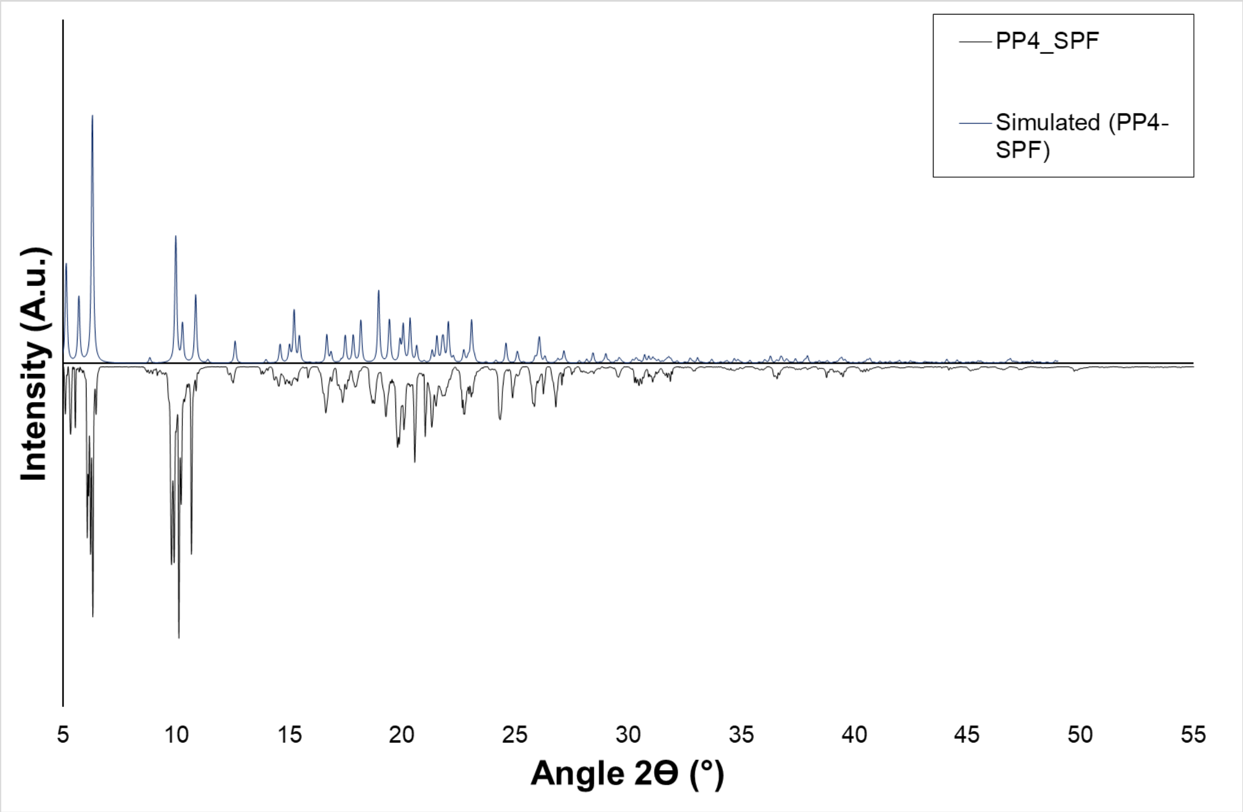

Figure S14 – PD-XRD of **PP$_4$-SPF** simulated from SC-XRD data (*top*) and of freshly isolated crystals of **PP$_4$-SPF** without grinding (*bottom*).

X-ray powder diffraction patterns were measured with a Bruker D8 Advance diffractometer with Cu-Kα radiation (λ = 1.54178 Å). All samples were mounted on a zero-background silicon single crystal sample holder with data collected in the range 5 – 55° (2θ). All samples were measured at room temperature and compared against the simulated patterns from the single crystal datasets (150 K).

SI 11. NMR Studies to determine thermal activation potential

Through NMR studies we were able to demonstrate that drying under high vacuum at 45 °C was sufficient for almost complete desorption of ethanol from PP_4-**SPF** (8 % mol/mol, i.e. 1 g **PP_4-SPF**: 6.4 mg EtOH). Drying under vacuum at 30 °C was insufficient with significant residual ethanol (85 % mol/mol, i.e. 1 g PP_4-**SPF_{act}**: 62.4 mg EtOH).

NMR analysis was carried out in MeOD from samples of **PP_4-SPF** crystals after thermal treatment. Crystals of **PP_4-SPF** was dried under vacuum at 30 °C to remove excess ethanol giving an initial ethanol content of 97 % mol/mol (0.97 eq EtOH: 1 eq **PP_4**). Subsequent heating under high vacuum resulted in a reduction of ethanol down to 9.7 % mol/mol after heating under vacuum at 45 °C for 12 hours. The recorded spectra are shown with the ethanol %, heating temperature and time. These results were reproducible with different crystal batches with only slight differences in ethanol percentage (± < 10 %).

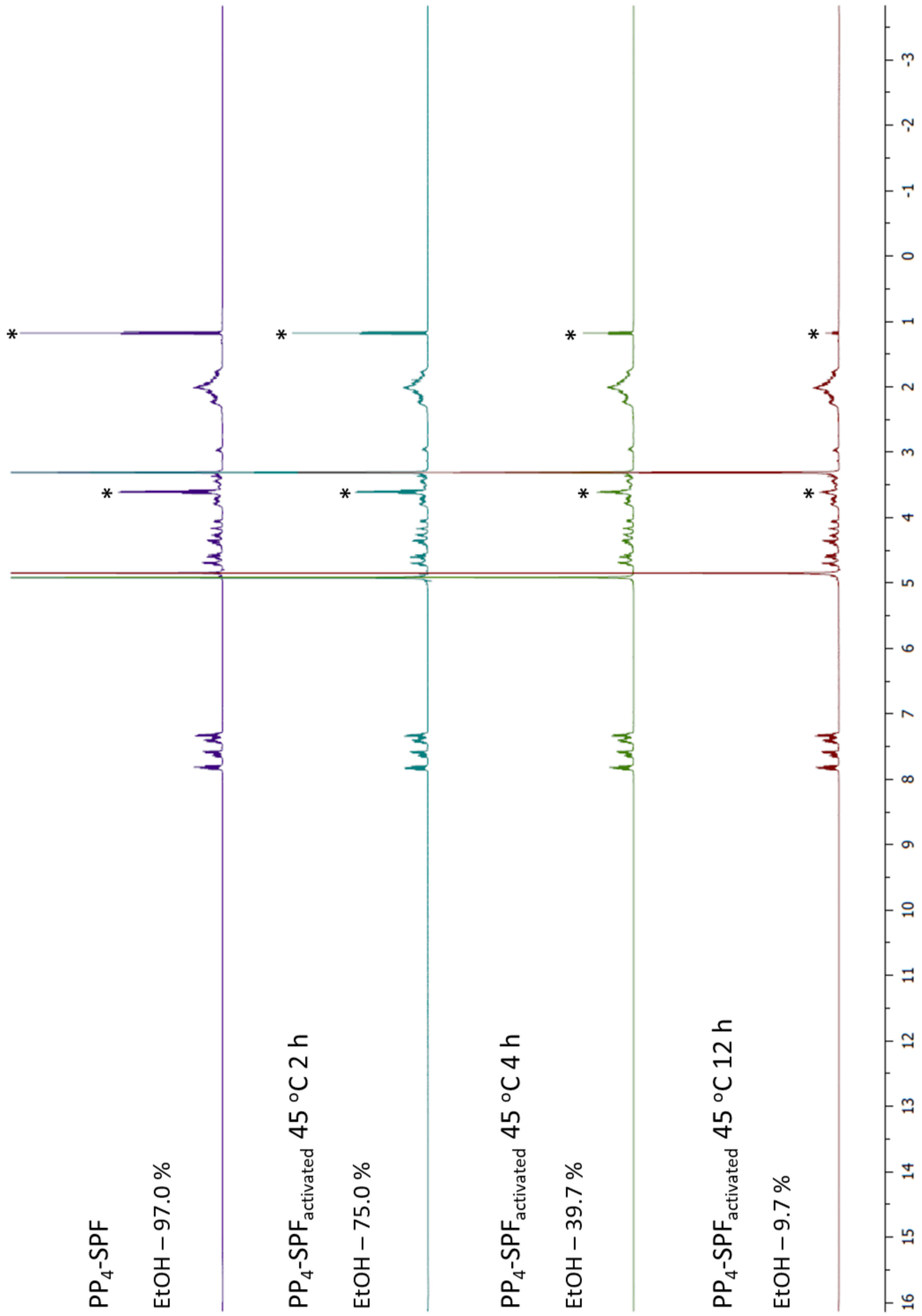

Figure S15 – ^{1}H NMR spectra of dissolved **PP$_4$-SPF** with varying degrees of thermal treatment, highlighting the remaining ethanol (mol/mol %) after treatment.

SI 12. Gas Adsorption Studies

The freshly isolated **PP_4-SPF** was activated by degassing at 45 °C for a total of 29 hours under dynamic vacuum, and the volumetric uptake of N_2 (77 K) and CO_2 (280 K) was measured. In both cases, no significant adsorption was observed across the loading range $P/P_0 = 10^{-7} – 1.0$ (N_2) or 0 – 1 bar (CO_2) consistent with adsorption only on the surfaces of the particles without penetration into the material, and a surface area <10m^2/g.

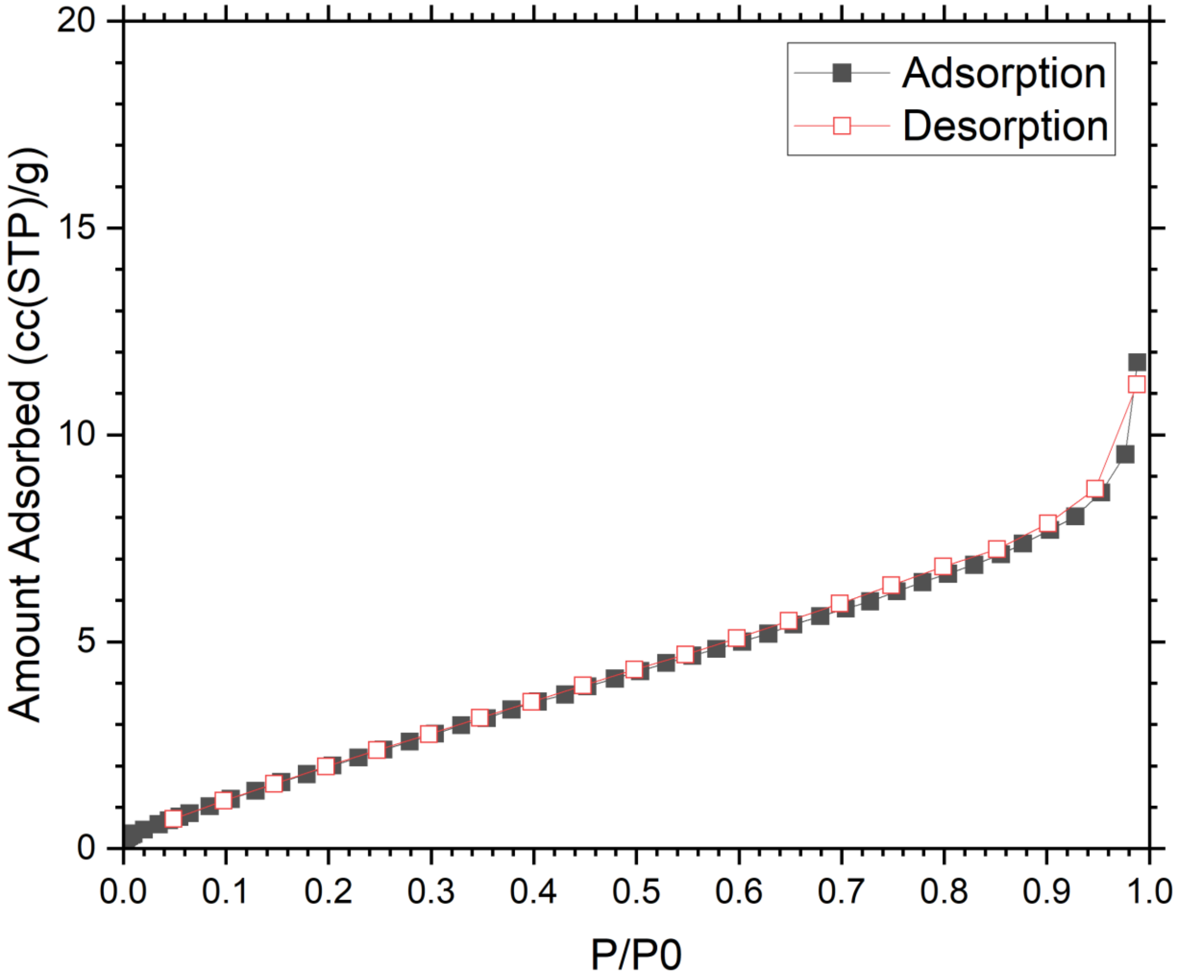

Figure S16 – N_2 adsorption isotherm for the desolvated **PP_4-SPF** (77 K)

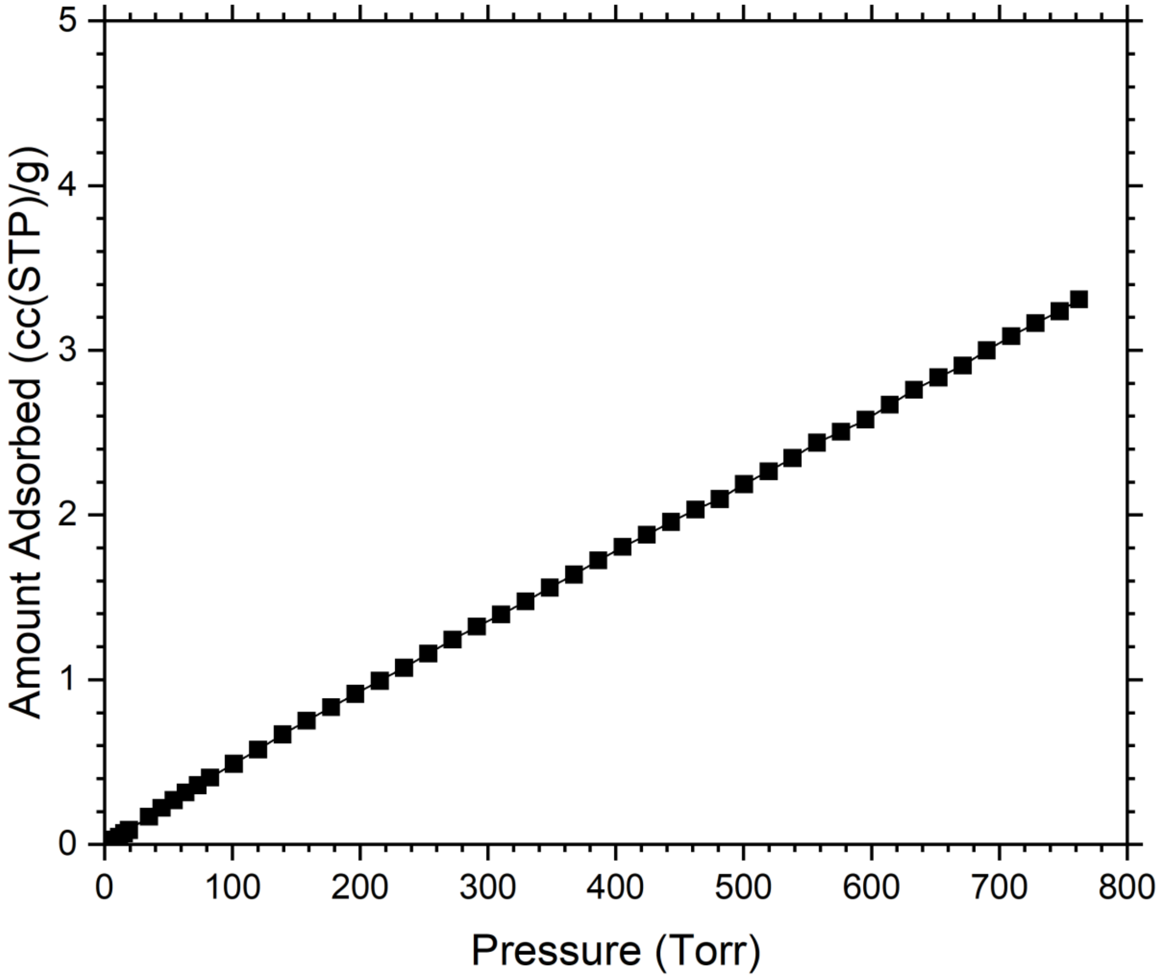

Figure S17 – CO_2 adsorption isotherm for desolvated **PP_4-SPF** (280 K).

Gas adsorption measurements were performed using a Quantachrome Autosorb iQ using N_2 and CO_2 at N4.5 grade or better. Temperature control was provided by a liquid nitrogen dewar for N_2 measurements and a Julabo recirculating chiller for CO_2 measurements. Samples were activated prior to the adsorption experiment at 45 °C for 6 hours dynamic vacuum provided by a rotary oil pump followed by evacuation under high vacuum provided by a turbomolecular pump at 45 °C for 15 and then 30 °C for a further 8 hours. The final desolvated sample mass was 165.2 mg.

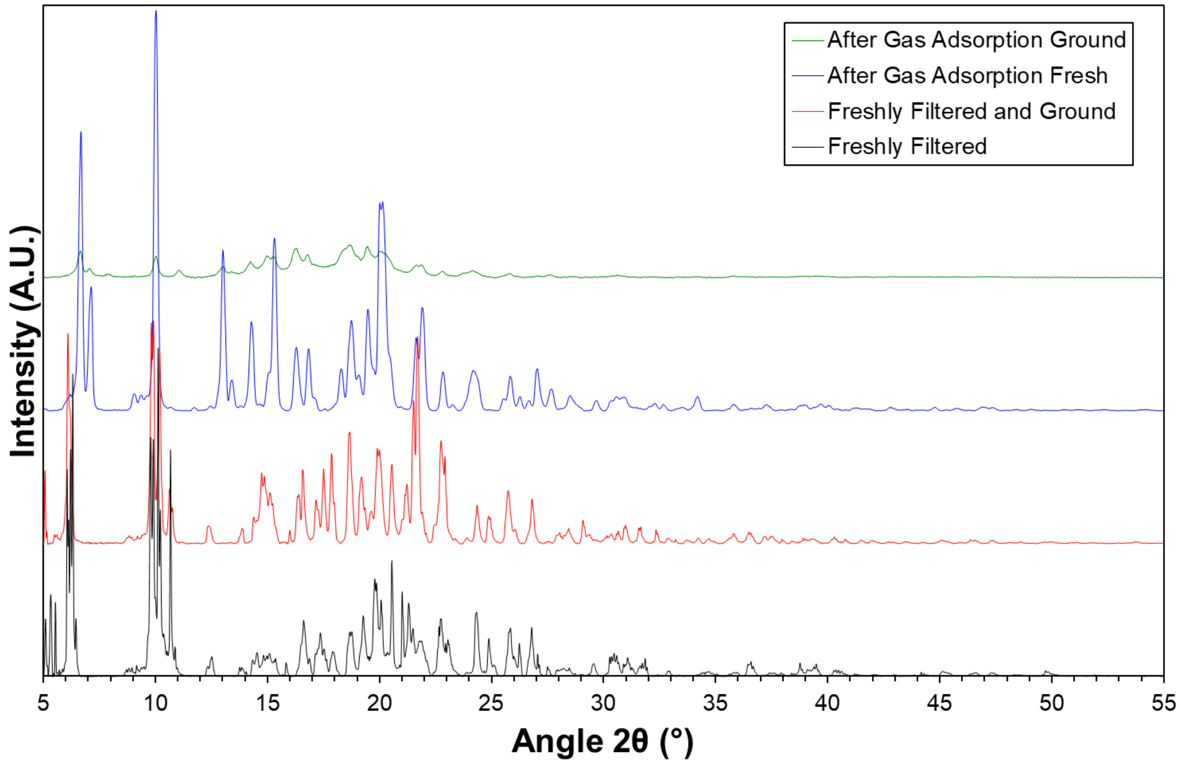

Figure S18 – X-ray powder diffraction pattern of the freshly isolated, solvated **PP_4-SPF** (**black**), this sample after grinding in air (**red**), the sample immediately following de-solvation and gas adsorption experiments (**blue**) and the desolvated sample after grinding (**green**).

SI 12.1 Single Crystal X-ray Diffraction of the Desolvated Phase (PP_4-SPF$_{act}$)

A single crystal from the desolvated phase following gas adsorption was subjected to single crystal X-ray diffraction. The crystal quality was poor following the phase transition on de-solvation, with cracking of the crystallites and a significant decrease in both resolution and intensity. Nonetheless, a new unit cell could be indexed (**SI 12.3**) which was consistent with a *ca* 15% contraction of the *a* unit cell edge compared to the pristine sample, with smaller increases on *b* and *c* while maintaining monoclinic $P2_1$ symmetry. Despite the poor-quality diffraction an approximate structure model could be obtained suggesting a rearrangement and contraction of the individual PP4 molecules and closing of the solvent channels following de-solvation. The X-ray powder diffraction pattern simulated from this model closely matches that obtained from the bulk de-solvated material (Figure **S20**), which supports the assignment of the non-porous structure model.

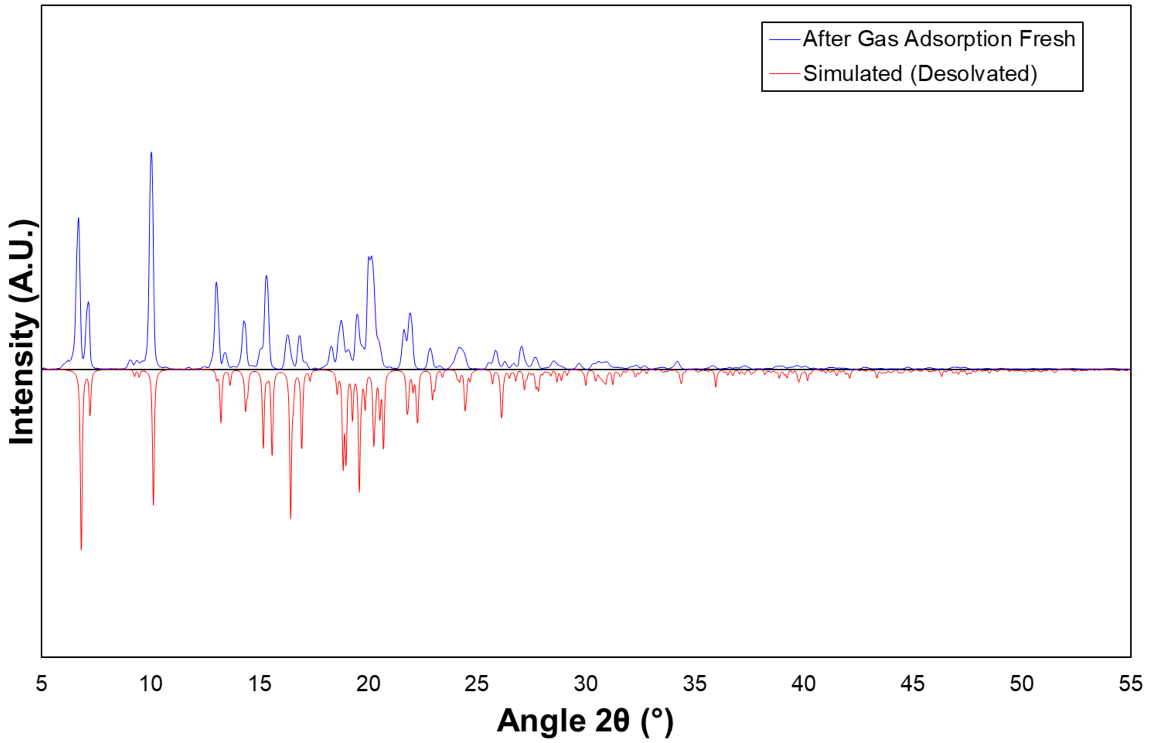

Figure S19 – X-ray powder diffraction pattern of the desolvated **PP_4-SPF** following gas adsorption (**blue**), and the simulated pattern from the single crystal X-ray dataset of a crystal following de-solvation (**red**)

SI 12.2 Re-solvation Experiments

A 30 mg sample of the desolvated material (following the gas adsorption experiments) was immersed in 5 mL of ethanol at room temperature in a sealed vial. Two samples were isolated from this suspension by filtration: one after 15 minutes, and one after 21 hours. Although both phases share an intense reflection at 10.1° (2θ), the immediate disappearance of the reflections at 6.8° and 7.2° in the desolvated phase and appearance of the reflection at 6.2°, as well as clear differences above 12° for the two phases indicate immediate regeneration of the solvated phase under these conditions.

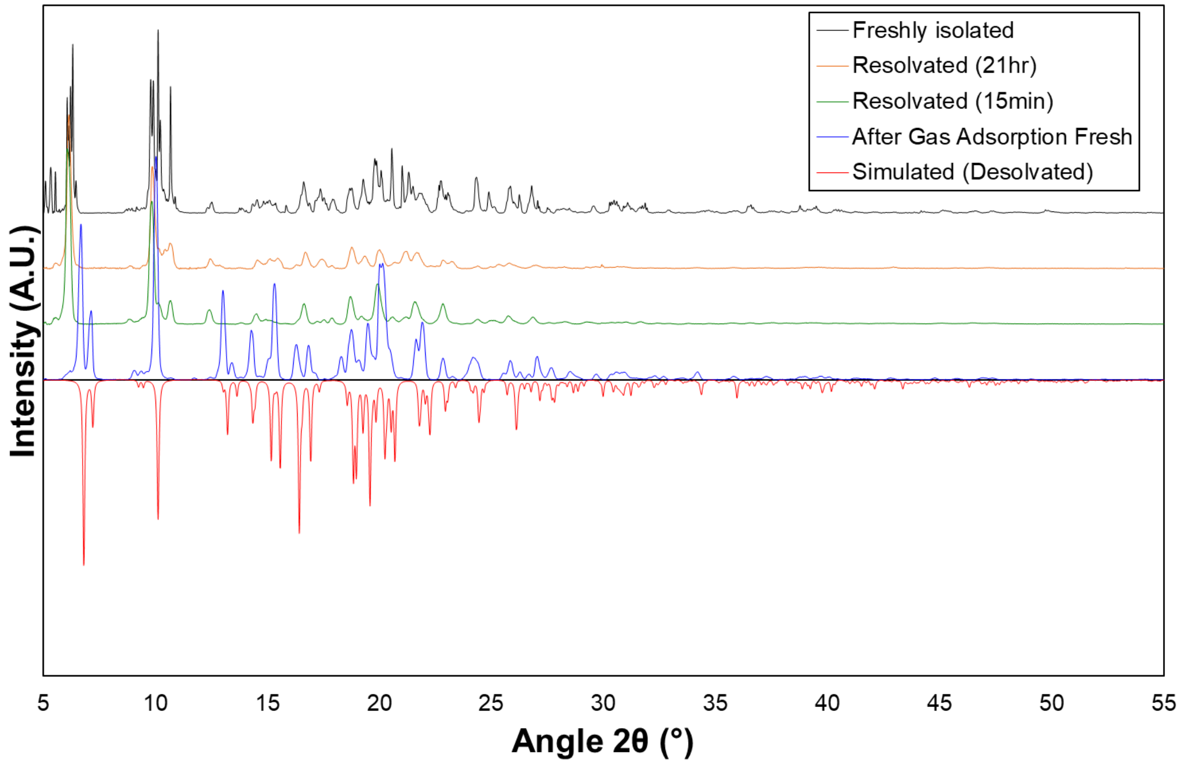

Figure S20 – Comparative X-ray powder diffraction patterns of **PP_4-SPF** desolvated (**blue**), following 15 minutes and 21 hours of soaking in ethanol (**green** and **yellow**, respectively), and the freshly isolated solvated material (**black**), compared with the simulated pattern for the desolvated sample (**red**).

SI 12.3 Crystal structure of PP_4-SPF_{Act}

The single crystal X-ray diffraction data for the desolvated material were collected with a Bruker D8 Quest ECO diffractometer using Mo-Kα radiation (λ = 0.71073 Å). A crystal was isolated from the sample following the gas adsorption experiments and mounted on a Mitegen micromount in NVH immersion oil and cooled to 150 K using an Oxford cryostream. Data collection and reduction were controlled using the Bruker APEX-3 suite of programs.[8] Multi-scan absorption corrections were applied using SADABS.[9] All data were solved using the intrinsic phasing routine within SHELXT[4] and refined on F^2 using full-matrix least squares procedures with SHELXL[5] within the OLEX-2 package.[2] The crystal showed very poor single crystallinity after the evacuation of the solvent, and no useful reflections were observed beyond 1 Å resolution, and all observed reflections showed a high degree of mosaicity. While the diffraction data could be solved to give a connectivity model and accurate unit cell parameters, the poor data quality precludes any meaningful refinement of anisotropic displacement parameters. This model is presented purely as a comparative connectivity model for comparison with the observed X-ray powder diffraction data; no attempt has been made to restrain U_{iso} parameters to avoid over-interpretation of the model.

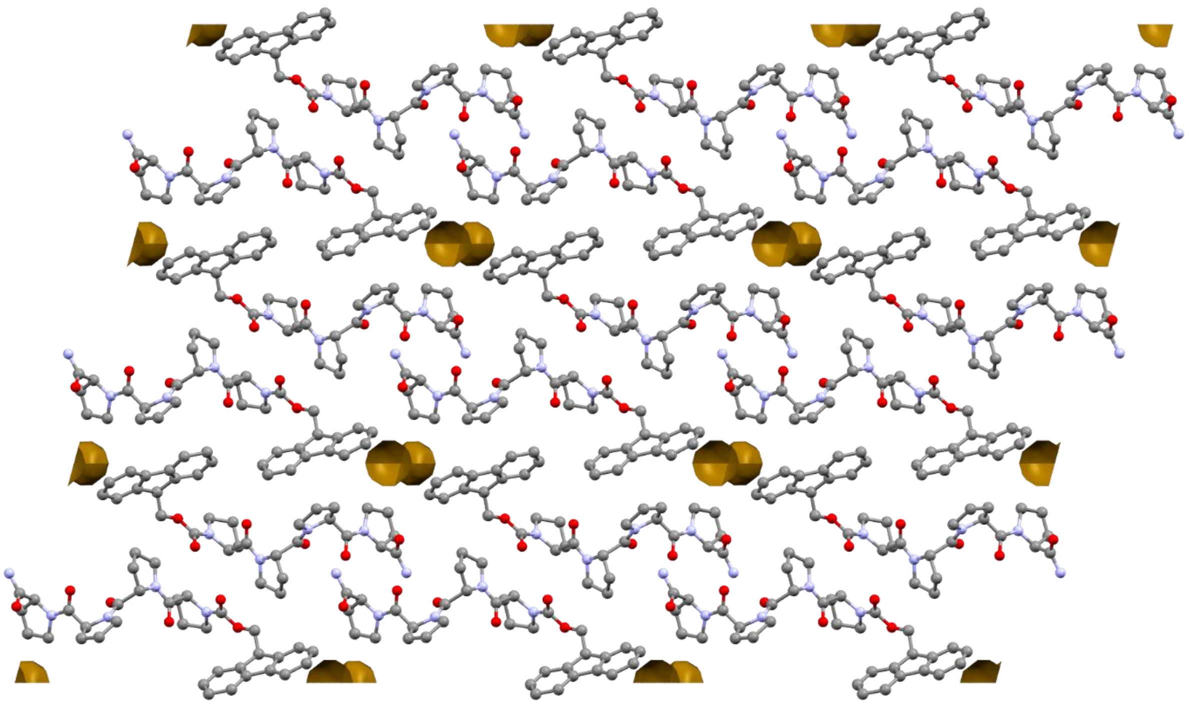

Figure S21 – Crystal structure of **PP$_4$-SPF$_{_Act}$** showing the desolvated structure highlighting significantly smaller void spaces ((yellow) 32.81 Å^3, 2.0 %-unit cell volume; Probe radius 1.2 Å, grid spacing 0.7 Å^2). Ball and stick model, packing 3x3x3.

**Crystal data and structure refinement for PP$_4$-SPF$_{_act}$**	
Identification code	**PP$_4$-SPF$_{_act}$** (PP4dried)
Empirical formula	$C_{35}H_{41}N_5O_6$
Formula weight	627.73
Temperature/K	150.0
Crystal system	monoclinic
Space group	$P2_1$
a/Å	13.438(6)
b/Å	6.535(3)
c/Å	19.346(8)
α/°	90
β/°	105.094(9)
γ/°	90
Volume/Å^3	1640.2(12)
Z	2
ρ_{calc}g/cm^3	1.271
μ/mm^{-1}	0.088
F(000)	668.0
Crystal size/mm^3	0.34 × 0.11 × 0.03
Radiation	MoKα (λ = 0.71073)
2Θ range for data collection/°	4.264 to 41.794
Index ranges	-13 ≤ h ≤ 13, -6 ≤ k ≤ 6, -18 ≤ l ≤ 19
Reflections collected	14635
Independent reflections	3452 [R_{int} = 0.1698, R_{sigma} = 0.1423]
Data/restraints/parameters	3452/1/185
Goodness-of-fit on F^2	1.141

Final R indexes [I>=2σ (I)]	R_1 = 0.1758, wR_2 = 0.3106
Final R indexes [all data]	R_1 = 0.2034, wR_2 = 0.3283
Largest diff. peak/hole / e Å^{-3}	0.59/-0.45
Flack parameter	-2.6(10)
CCDC No.	2156434

SI 13. Framework Host-Guest chemistry:

Subsequent to de-solvation of the framework and the observation of apparent reinflation upon soaking in ethanol solution (Figure S19), reinflation on **PP_4-SPF_{act}** with various guest molecules was then attempted (Table **S3**). Both ^{1}H NMR (SI 13.3) and PD-XRD (SI 13.4) were used to confirm the degree of encapsulation of guest molecules and the change from the starting phase of the desolvated material after soaking with the guest molecule. The percentage of encapsulated guest, hexane (used as guest diluent) and residual ethanol obtained from ^{1}H NMR (SI 13.3) analyses are summarized in the table below (Table S3). Chiral HPLC was then used to determine the enantioselectivity of the SPF towards 1-Phenylethanol (SI13.5).

Table S3: Determination of encapsulated guest[d]

Entry	Sample treatment	Time /h	guest content /mol%[c]	Hexane content /mol%[c]	Resid. EtOH /mol%[c]
1	Washed with **hexane** (**PP_4_SPF**)	-	-	8	91
2	**Activated** under vac 45 °C (**PP_4-SPF_{act}**)	16	-	-	12
3	**EtOH 5%** /Hex	1.5	54	61	-
4	**EtOH vapour**	26	14	-	-
5	(±)**PhEtOH 5 %** /Hex	1.5	36	20	8.
6	(±)**PhEtOH 5 %** /Hex	16	59	36	6
7	(±)**PhEtOH 2 eq** /Hex	1.5	33	17	5
8	Amorphous **PP_4** Inc. (±)**PhEtOH** 5 %/Hex	1.5	7	16	78
9	**Acetone 5 %** /Hex	16	28 [a]	51	0
10	**THF 5 %** /Hex	16	23[a]	48	2
11	EtOAc **5 %** /Hex	16	33	50	2
12	**Hexane**	16	-	4	7
13	**Toluene 5 %** /Hex	16	33	30	5
14	**1-Bromohexane 5 %** /Hex	16	15	32	2
15	**I_2** saturated hexane	336	12[b]	18	2

[a] Integral of clean spectrum of **PP_4** at same ppm range subtracted from solvent peak integral for % calculation due to overlapping peaks of peptide and guest

[b] Iodine content determined via molecular occupancy from SC-XRD data

[c] All guest mol% calculated against moles of peptide

[d] **General procedure for guest soaking:** A sample of **PP_4-SPF_{act}** (≈5-10 mg) was placed in a premixed solution of guest (typically 5 %) in hexane (300 µL, HPLC grade) for 16 hours before decanting the solution and washing five times with fresh hexane to remove excess guest. The solid was then taken for PD-XRD analysis (**SI 13.4**). The sample was then fully dissolved in MeOD before recording the ^{1}H NMR spectra (**SI 13.3**). The peaks at 7.8-7.85 ppm for two of the Fmoc CH protons were used as the peptide reference, set as 2H, and the integrals of the guest's peaks were used to calculate the mole percentage against the peptide (i.e. 50 % guest = 2 eq. peptide: 1 eq. guest).

For (±) 1-Phenylethanol studies the NMR sample was then dried under compressed air before dissolving in propan-2-ol (30 µL), and diluting with hexane (570 µL) causing the peptide to precipitate. This suspension was then filtered through a 0.2 µm syringe filter and the filtrate taken for chiral HPLC analysis (*further details **SI 13.5***).

SI 13.1 Crystal Structure of PP_4-SPF@I_2

The crystal structure was obtained from orange/yellow crystals, crystallised from a supersaturated hot ethanol solution as previously, before they were dried under vacuum at 45 ^oC overnight and covered with a saturated solution of iodine in hexane for a week. The crystals were stable outside of solution at room temperature showing no signs of deterioration over the timeframe of the experiment.

The experiment was carried out by mounting a crystal without using an inert oil to maximize the guest loss. The crystal was mounted at 290 K, then cooled down to 150 K with a cooling rate of 280 K/h and analyzed *via* single crystal X-ray diffraction. A second data collection was performed after heating the same crystal directly on the mount at 323 K for 4 hours and cooling it down to 150 K with the same rate used for the previous collection. The guest molecules within the pores have been modeled for each of the two sets of data in the same way: the occupancies of the iodine atoms were allowed to freely refine while keeping the atoms isotropic and fixing the isotropic atomic displacement parameters to the same value. The analogous treatment of the two data sets allowed us to obtain the values of 12.3 % and 5.8 % respectively for the chemical occupancies of the iodine molecules respectively before and after the thermal treatment.

In the structures reported (CCDC-2127748 and CCDC-2127749), before and after the thermal treatment, the iodine atoms were treated anisotropically and their anisotropic displacement parameters were kept equivalent. For the **PP_4-SPF@I_2** structure the carbon atom C14 of the ring of pro2 ($C_γ$) was split into two components with a total occupancy of 1. The anisotropic displacement parameters were set to be equivalent for this atom and for the nearest carbon atoms. For the **PP_4-SPF@I_{2_Heated}** structure three carbon atoms (C13, C14 and C15) of the ring of pro2 ($C_{ß,γ,δ}$) were split into two components with a total occupancy of 1. The anisotropic displacement parameters were set to be equivalent for these atoms in both the components. Within the pro2 ring, both the N-C and C-C distances were fixed to be the same between the two components.

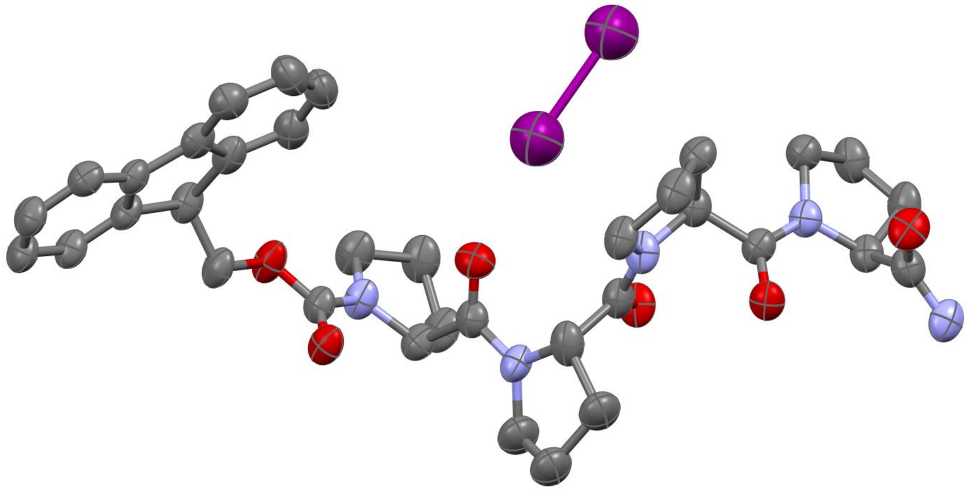

Figure S22 – Mercury ellipsoid representation (50 % probability) of the asymmetric unit of **PP_4-SPF@I_2**, hydrogen atoms removed for clarity

SI 13.2 Crystal data and structure refinement for PP_4-SPF@I_2 and PP_4-SPF@I_{2_Heated}

Identification code	**PP_4-SPF_I_2**	**PP_4-SPF_I_{2_Heated}**
Empirical formula	$C_{35}H_{41}I_{0.27}N_5O_6$	$C_{35}H_{40}I_{0.14}N_5O_6$
Formula weight	661.99	645.12
Temperature/K	150(2)	150(2)
Crystal system	monoclinic	monoclinic
Space group	$P2_1$	$P2_1$
a/Å	16.523(2)	16.5376(15)
b/Å	6.3229(5)	6.2977(4)
c/Å	18.208(4)	18.2023(16)
α/°	90	90
β/°	109.803(19)	109.744(10)
γ/°	90	90
Volume/Å^3	1789.8(5)	1784.3(3)
Z	2	2
ρ_{calc}g/cm^3	1.228	1.201
μ/mm^{-1}	2.487	1.644
F(000)	697.0	681.0
Crystal size/mm^3	0.235 × 0.073 × 0.035	0.235 × 0.073 × 0.035
Radiation	Cu Kα (λ = 1.54184)	Cu Kα (λ = 1.54184)
2Θ range for data collection/°	8.882 to 143.29	8.872 to 143.6
Index ranges	-20 ≤ h ≤ 13, -7 ≤ k ≤ 7, -22 ≤ l ≤ 22	-20 ≤ h ≤ 20, -7 ≤ k ≤ 7, -22 ≤ l ≤ 13
Reflections collected	12910	13057
Independent reflections	6762 [R_{int} = 0.0564, R_{sigma} = 0.0785]	6779 [R_{int} = 0.0442, R_{sigma} = 0.0598]
Data/restraints/parameters	6762/1/421	6779/8/426
Goodness-of-fit on F^2	1.072	1.059
Final R indexes [I>=2σ (I)]	R_1 = 0.1062, wR_2 = 0.2855	R_1 = 0.0952, wR_2 = 0.2548
Final R indexes [all data]	R_1 = 0.1311, wR_2 = 0.3075	R_1 = 0.1140, wR_2 = 0.2763
Largest diff. Peak/hole / e Å^{-3}	0.85/-0.36	1.25/-0.44
Flack parameter	0.08(3)	0.31(3)

SI 13.3 ^{1}H NMR studies of SPF after guest encapsulation

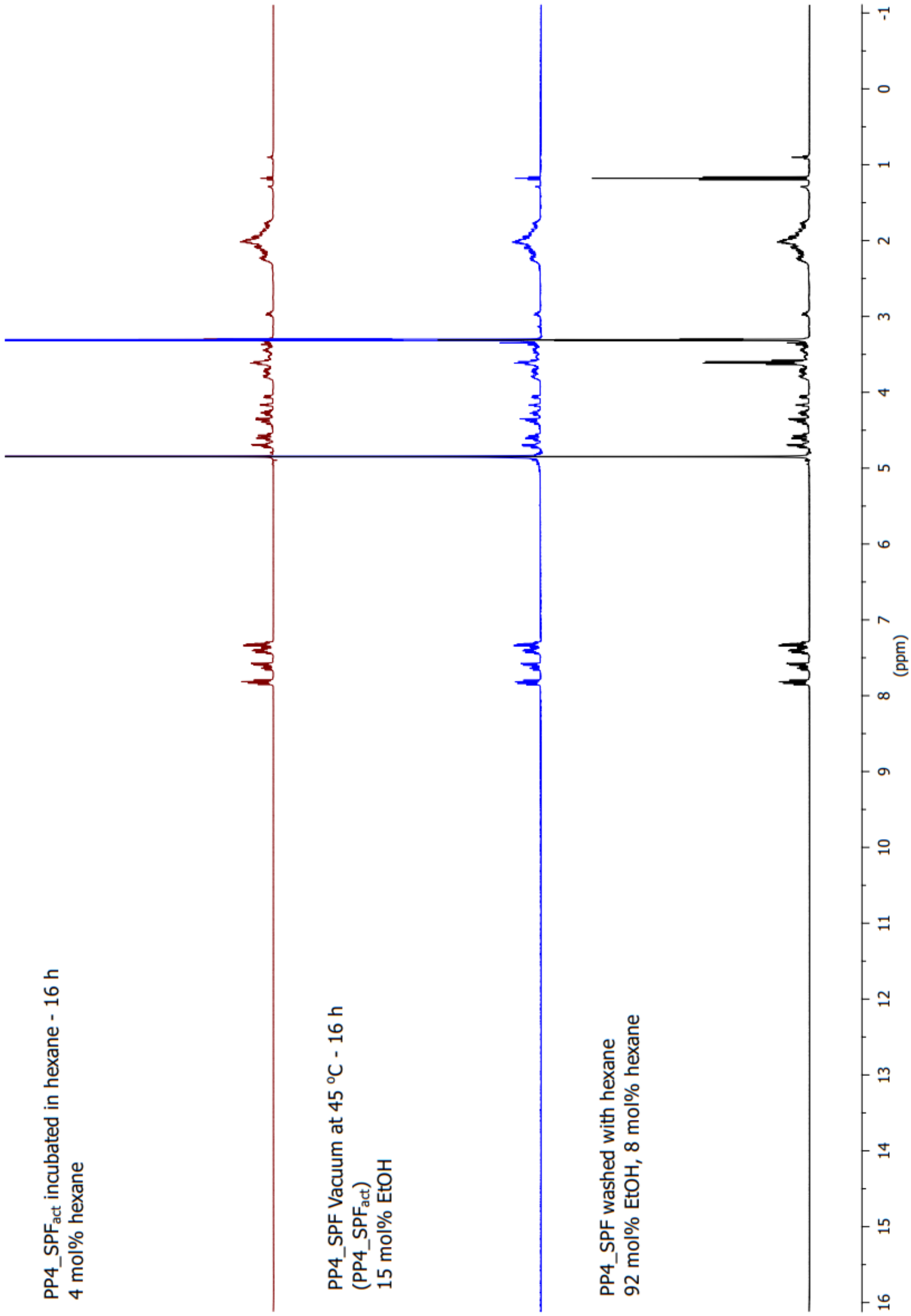

Figure S23 - ^{1}H NMR (400 MHz, MeOD) of **PP$_4$-SPF** washed with hexane (HPLC grade, x 5) (**black**, bottom), PP4-SPF after activation at 45 °C under vacuum for 16 h (**blue**), and **PP$_4$-SPF$_{act}$** after soaking in hexane for 16 h (**red**, top).

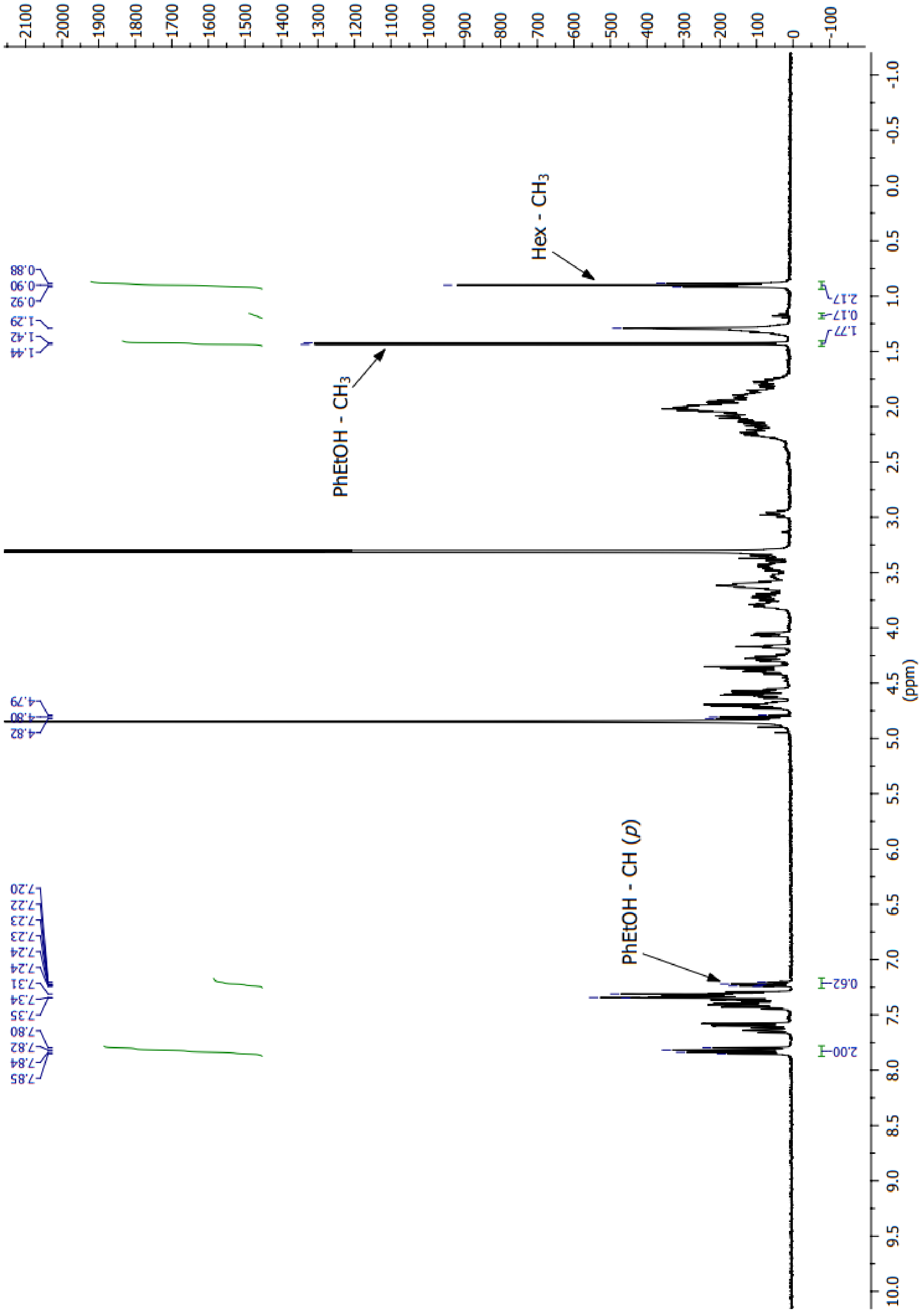

Figure S24 - **^{1}H NMR (400 MHz, MeOD)** of **PP_4-SPF_{act}** after soaking in PhEtOH (5 %, 300 μL in hexane) for 16 h and washed with hexane (HPLC grade, x 5) before drying for 30 mins at rt.

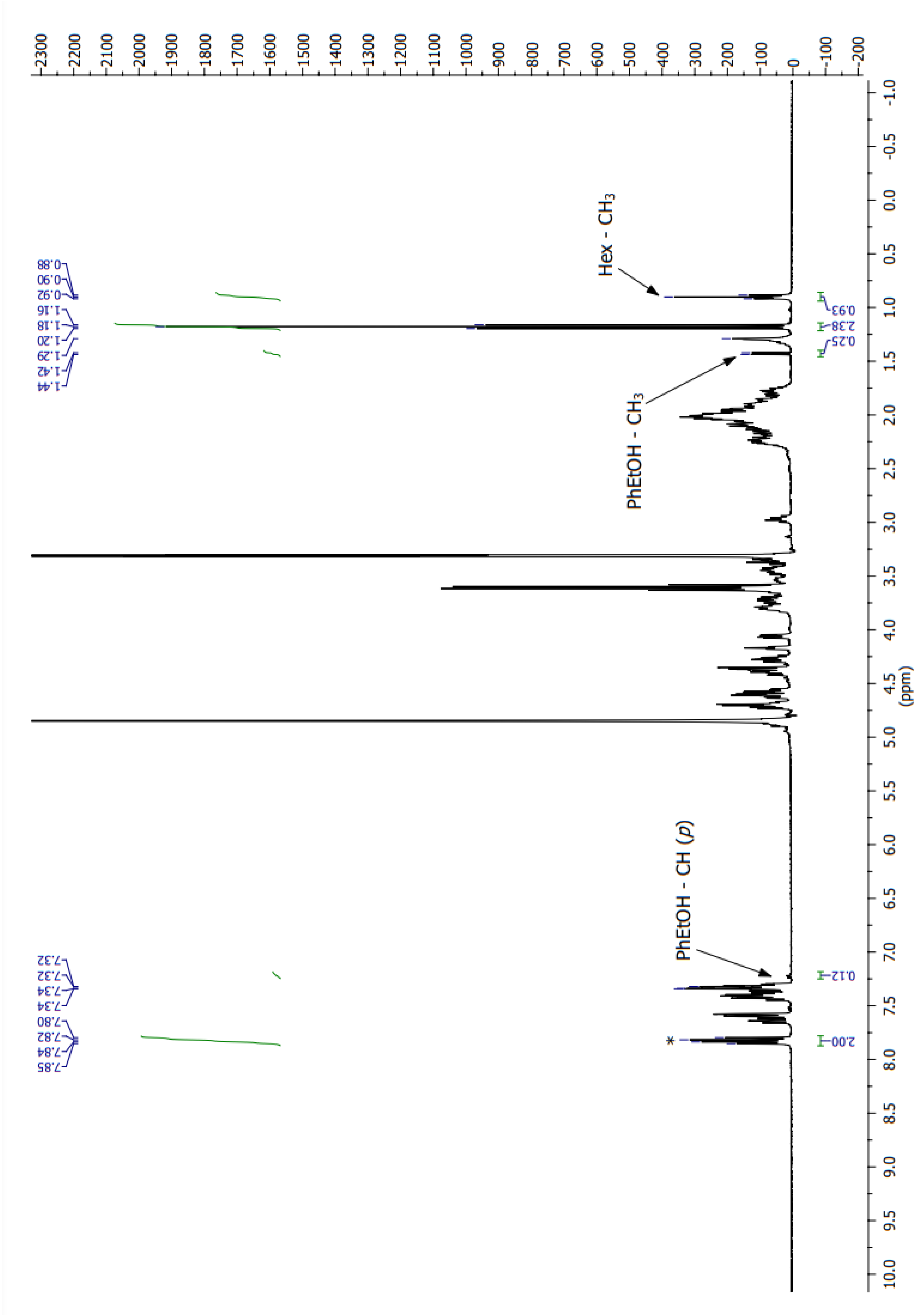

Figure S25 - **^{1}H NMR (400 MHz, MeOD)** of **PP_4** after soaking in PhEtOH (5 %, 300 µL in hexane) for 1.5 h and washed with hexane (HPLC grade, x 5) before drying for 30 mins at rt.

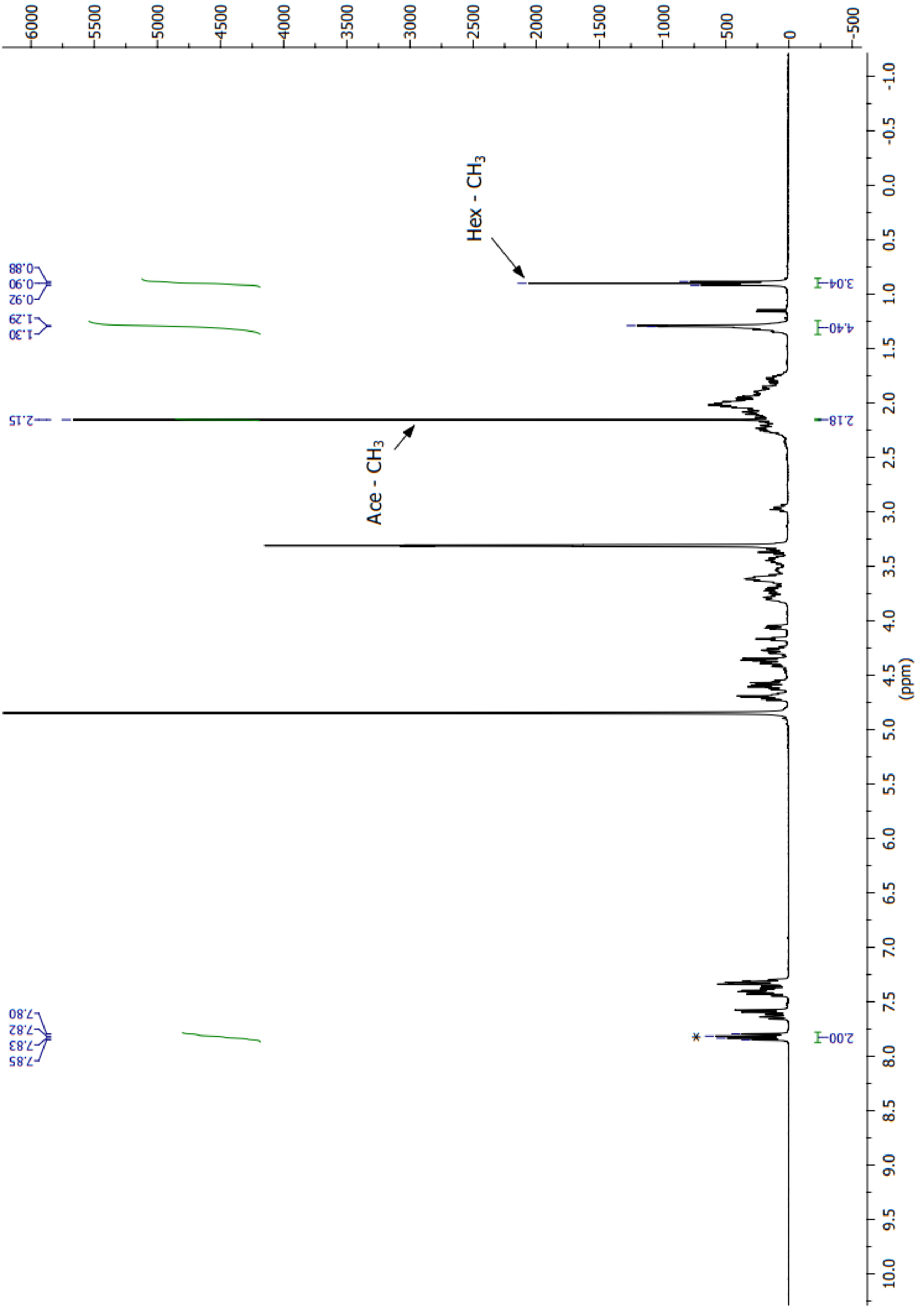

Figure S26 - **^{1}H NMR (400 MHz, MeOD)** of **PP$_4$-SPF$_{act}$** after soaking in Acetone (5 %, 300 µL in hexane) for 16 h and washed with hexane (HPLC grade, x 5) before drying for 30 mins at rt.

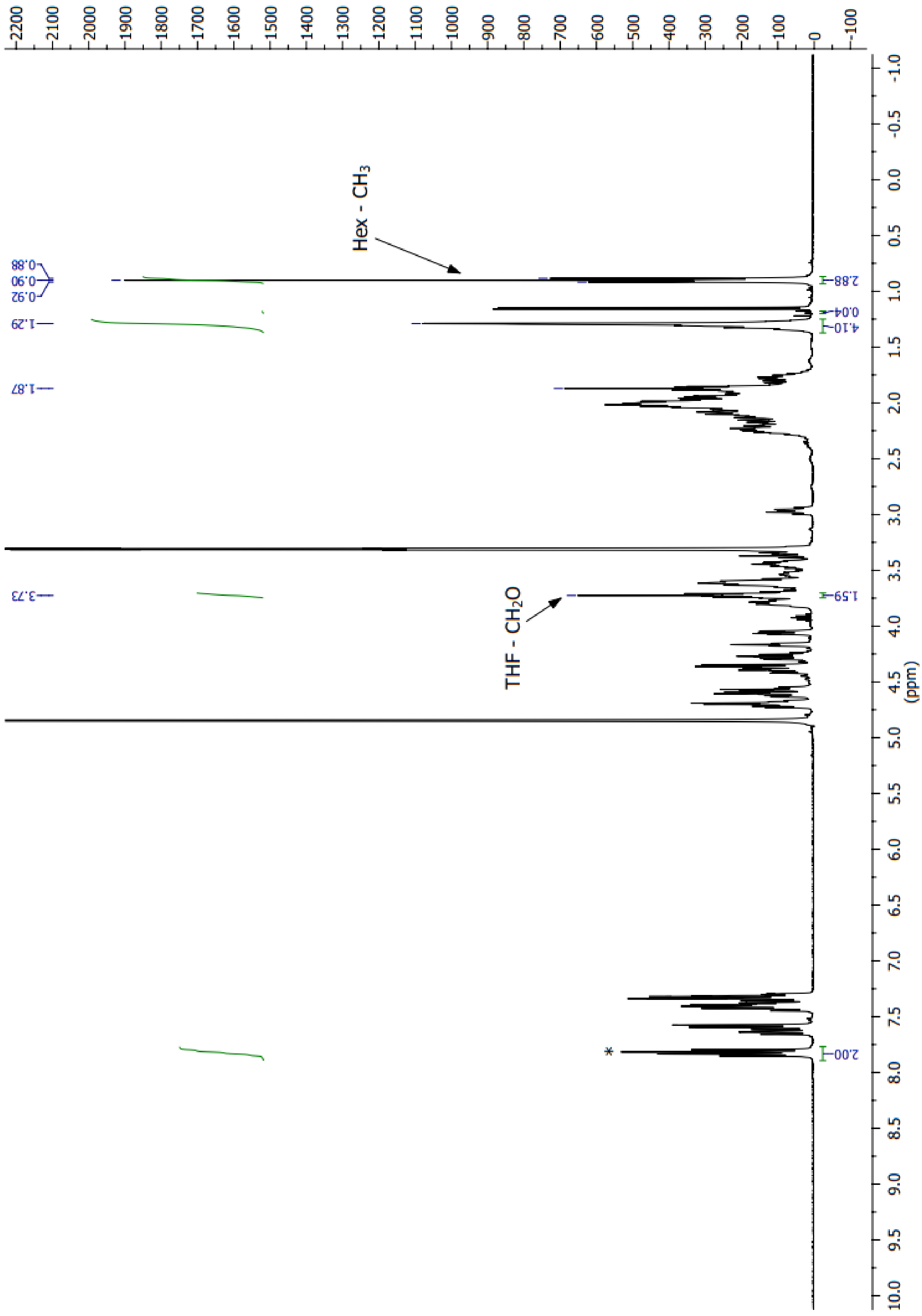

Figure S27 - **^{1}H NMR (400 MHz, MeOD)** of **PP$_4$-SPF$_{act}$** after soaking in THF (5 %, 300 µL in hexane) for 16 h and washed with hexane (HPLC grade, x 5) before drying for 30 mins at rt.

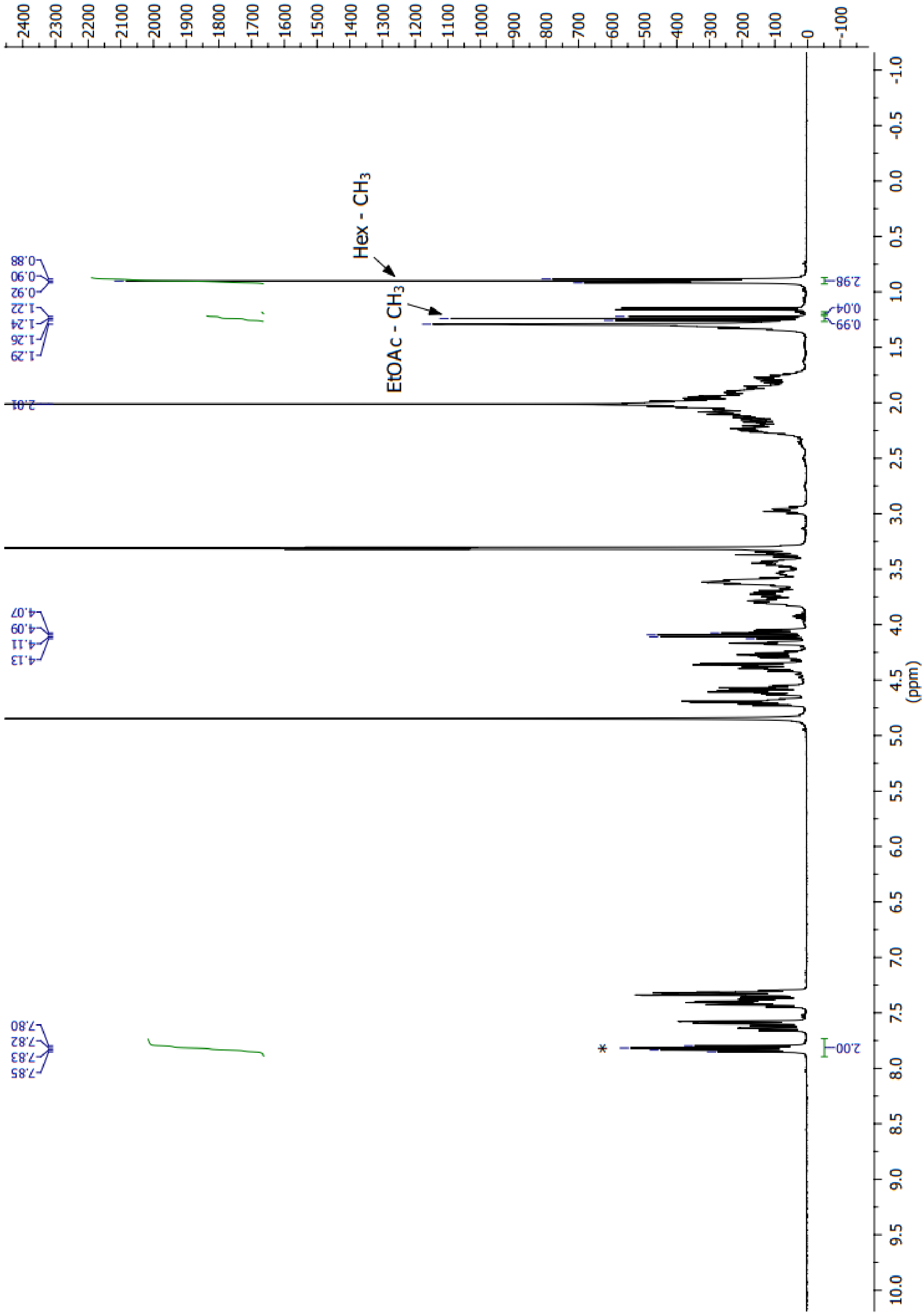

Figure S28 - **^{1}H NMR (400 MHz, MeOD)** of **PP_4-SPF_{act}** after soaking in ethyl acetate (5 %, 300 µL in hexane) for 16 h and washed with hexane (HPLC grade, x 5) before drying for 30 mins at rt.

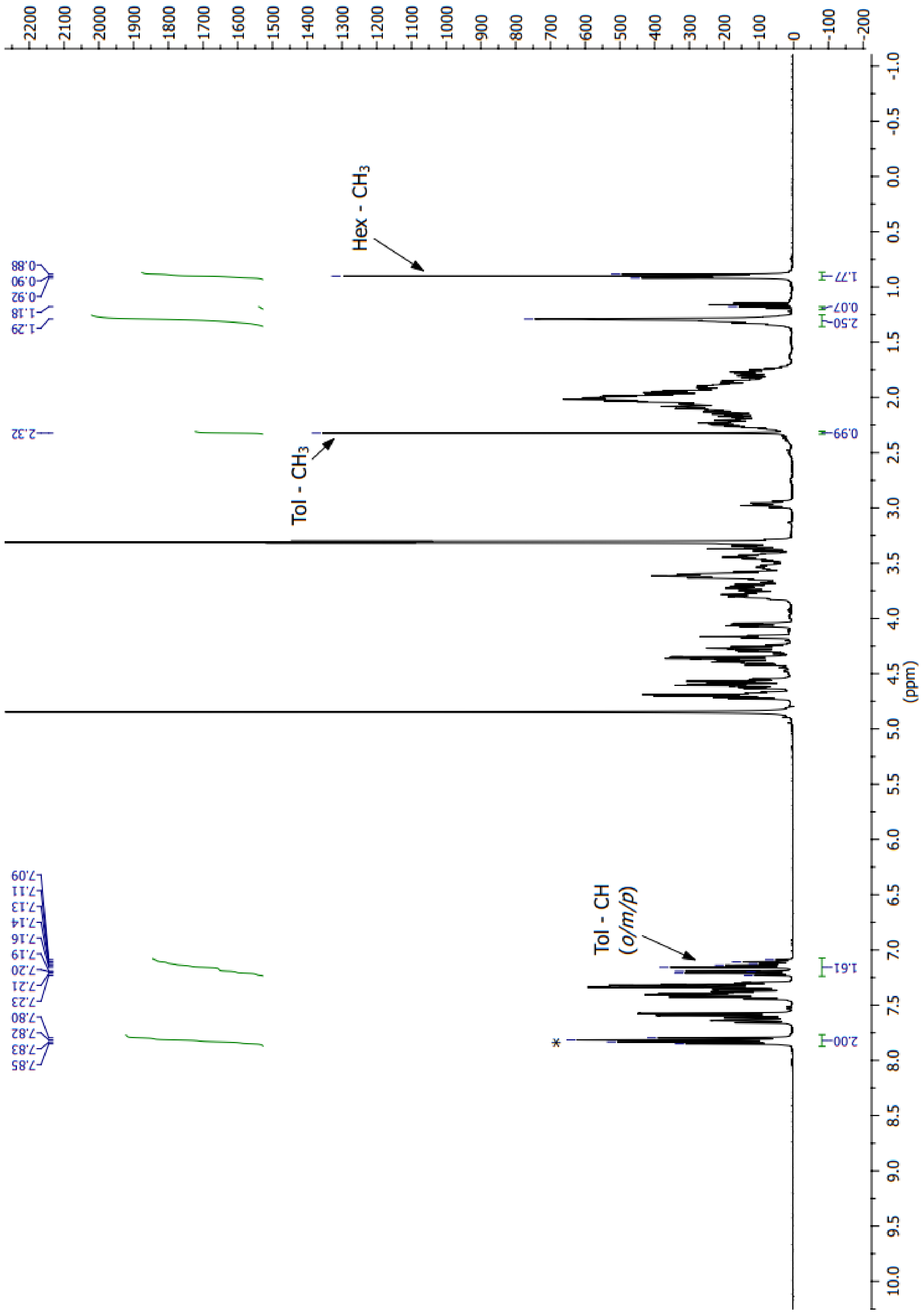

Figure S29 - **^{1}H NMR (400 MHz, MeOD)** of **PP_4-SPF_{act}** after soaking in toluene (5 %, 300 µL in hexane) for 16 h and washed with hexane (HPLC grade, x 5) before drying for 30 mins at rt.

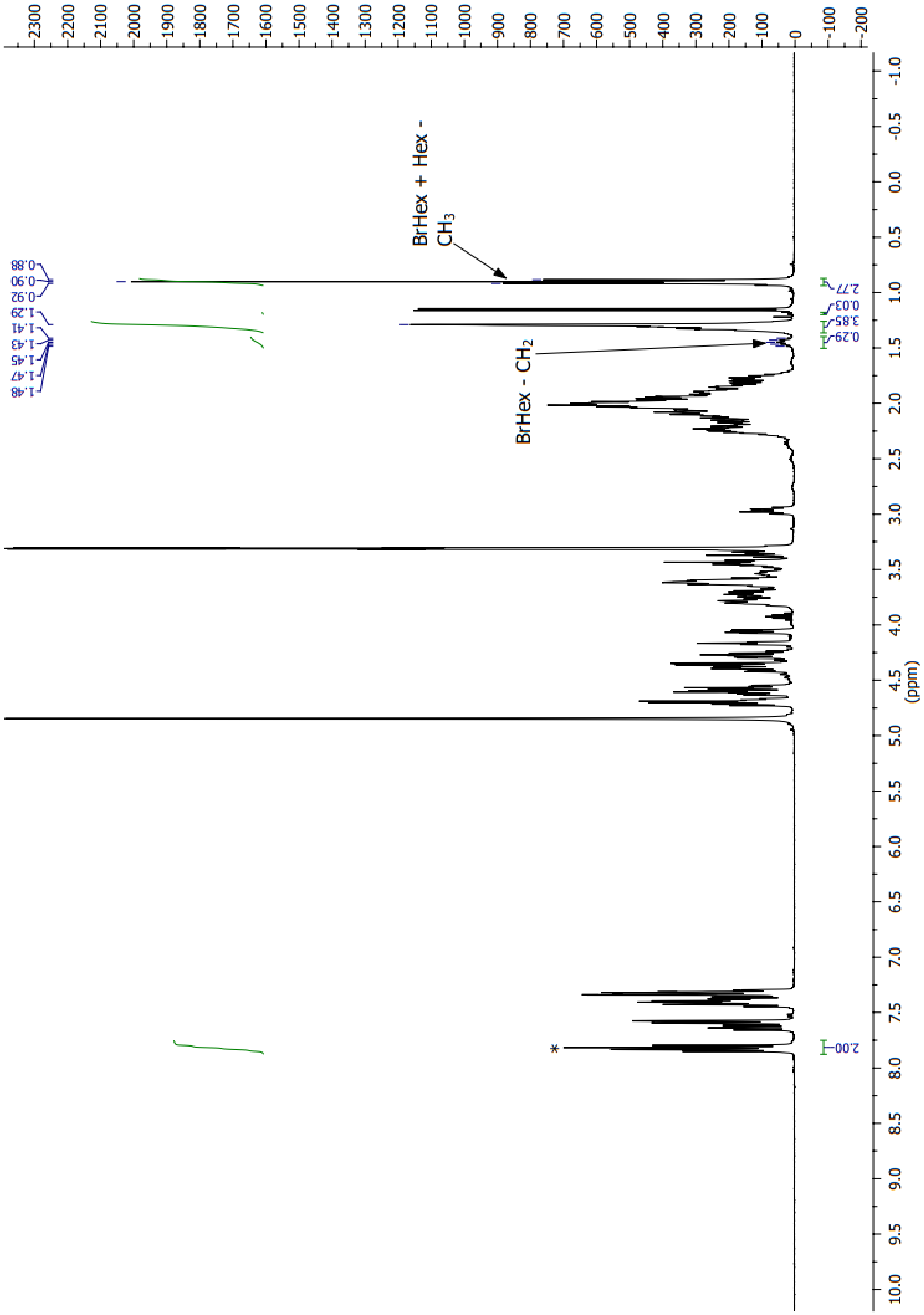

Figure S30 - **^{1}H NMR (400 MHz, MeOD)** of **PP_4-SPF_{act}** after soaking in 1-bromohexane (5 %, 300 µL in hexane) for 16 h and washed with hexane (HPLC grade, x 5) before drying for 30 mins at rt.

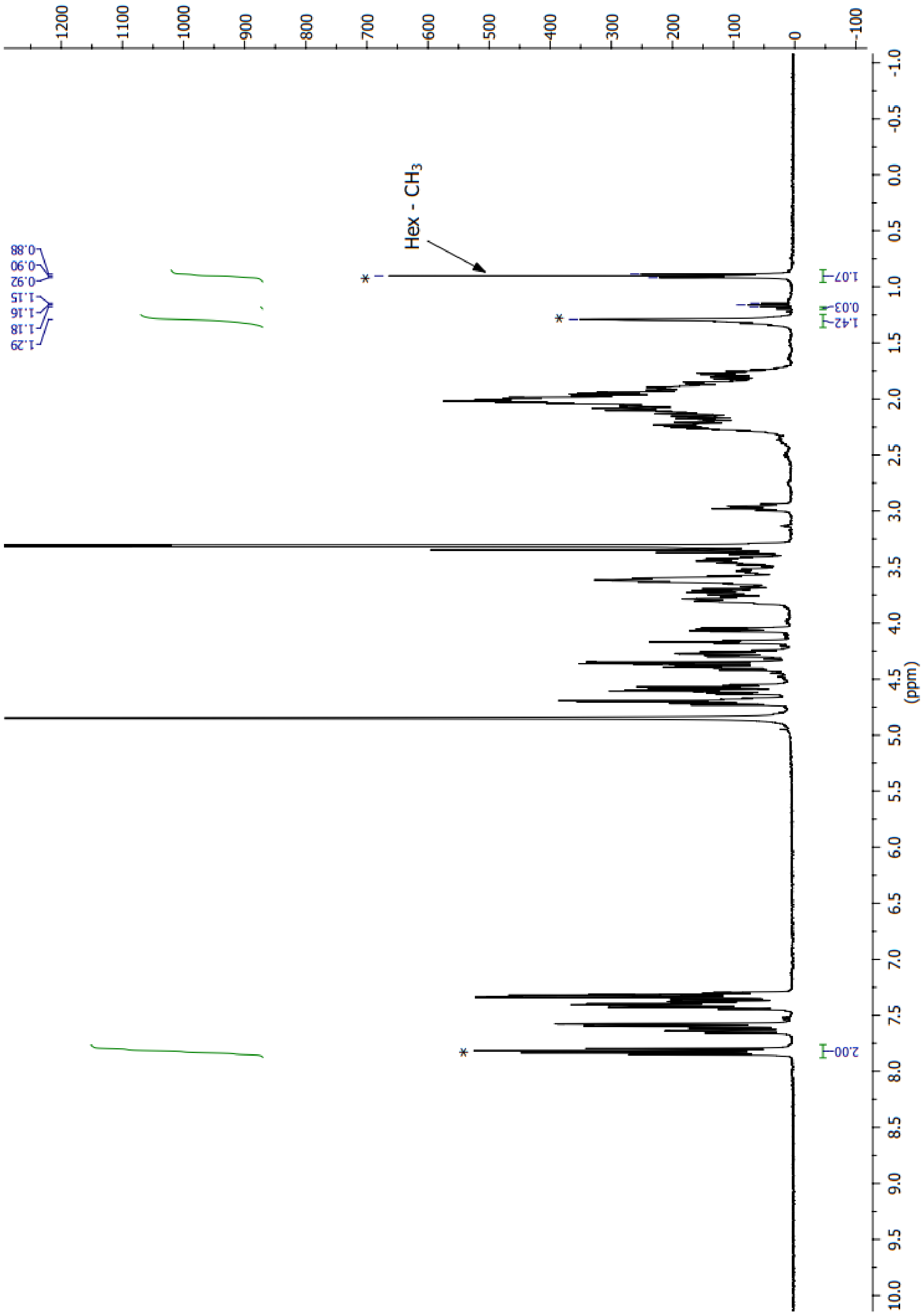

Figure S31 - **^{1}H NMR (400 MHz, MeOD)** of **PP$_4$-SPF$_{act}$** after soaking in saturated iodine solution (300 µL in hexane) for 16 h and washed with hexane (HPLC grade, x 5) before drying for 30 mins at rt.

SI 13.4 Powder Diffraction data after SPF_{act} guest soaking

X-ray powder diffraction patterns for host-guest studies were collected on a Rigaku Miniflex 600 using a Cu radiation source and measurements were performed at room temperature (Scan range 2Θ; 5-55°, Step; 0.02 °, scan speed; 0.8 °min^{-1}).

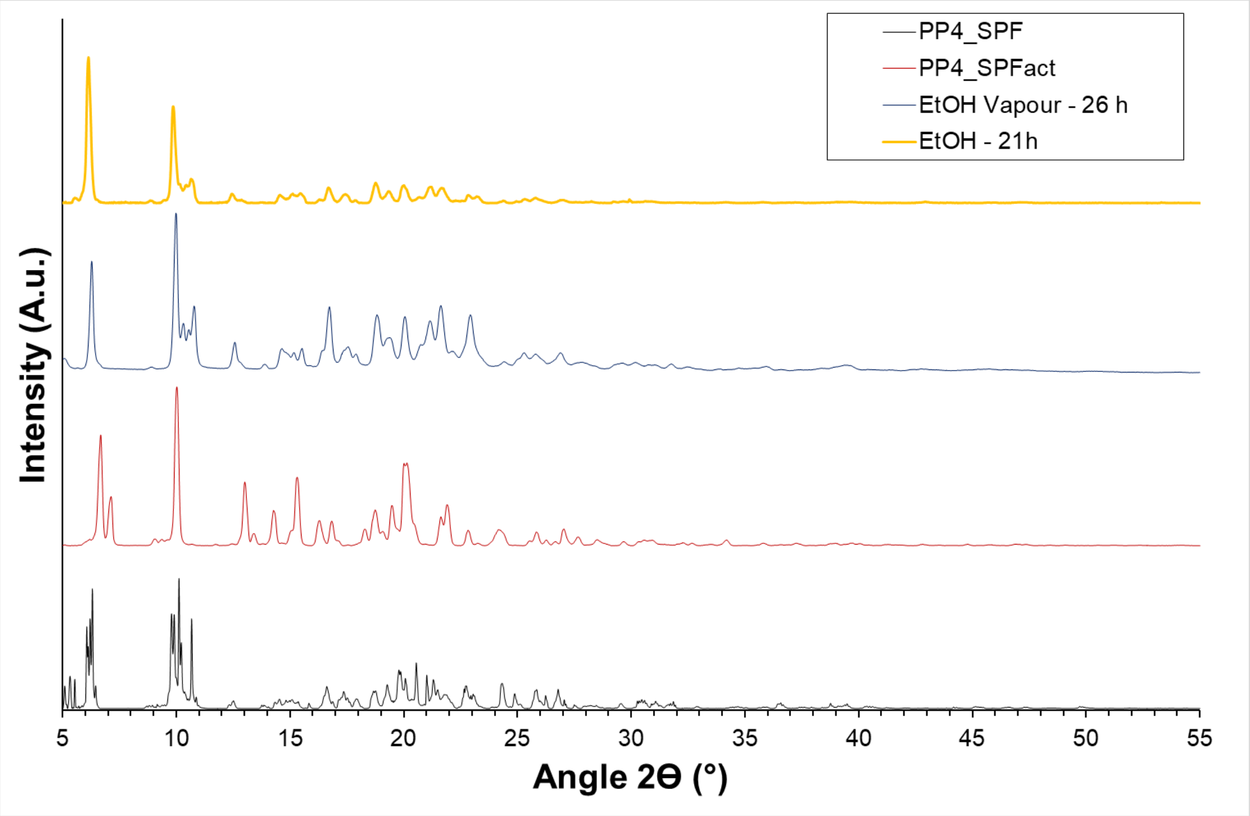

Figure S32 – PD-XRD of fresh crystals of **PP_4-SPF** unground (black, bottom), **PP_4-SPF** after activation at 45 °C under high vacuum (provided by a turbomolecular pump) (**red**), **PP_4-SPF_{act}** after soaking in a chamber saturated with ethanol vapour for 26 h (**blue**), and **PP_4-SPF_{act}** after soaking in ethanol solution for 21 h.

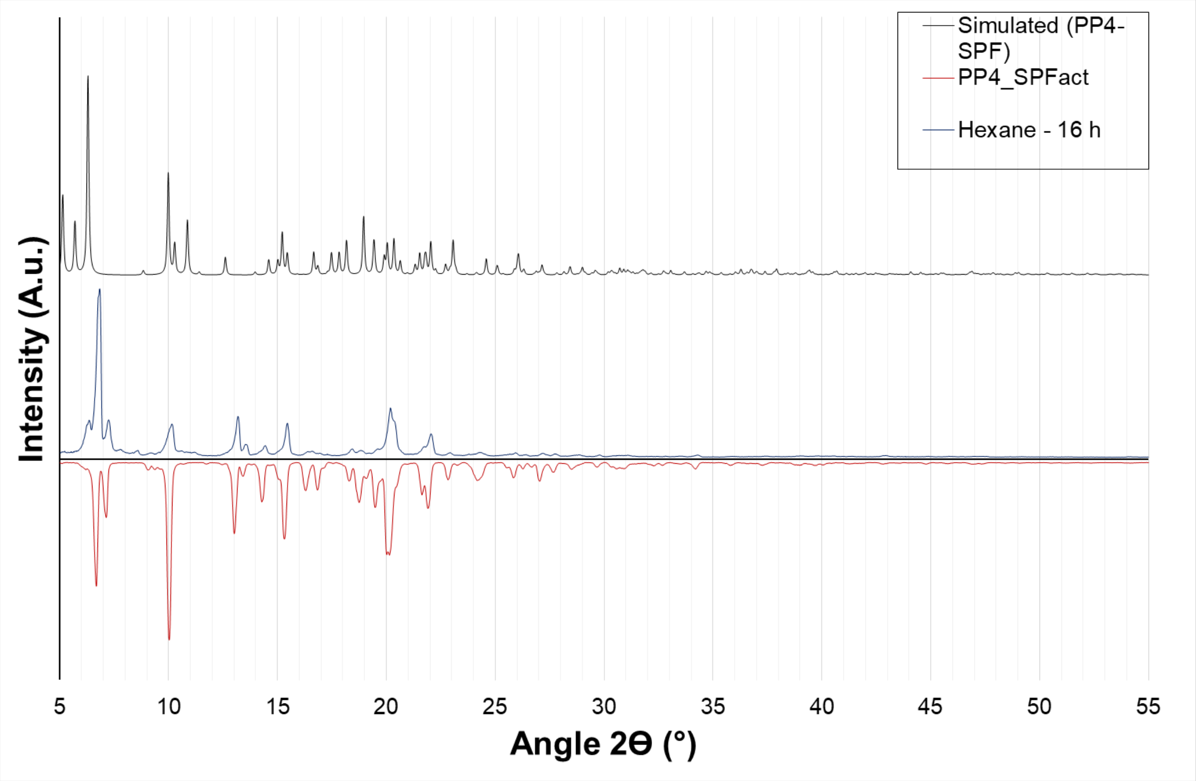

Figure S33 – PD-XRD simulated for **PP$_4$-SPF** (**black**, top), **PP$_4$-SPF** after activation at 45 °C under high vacuum (provided by a turbomolecular pump) (**red**), **PP$_4$-SPF$_{act}$** after soaking in hexane (**blue**)

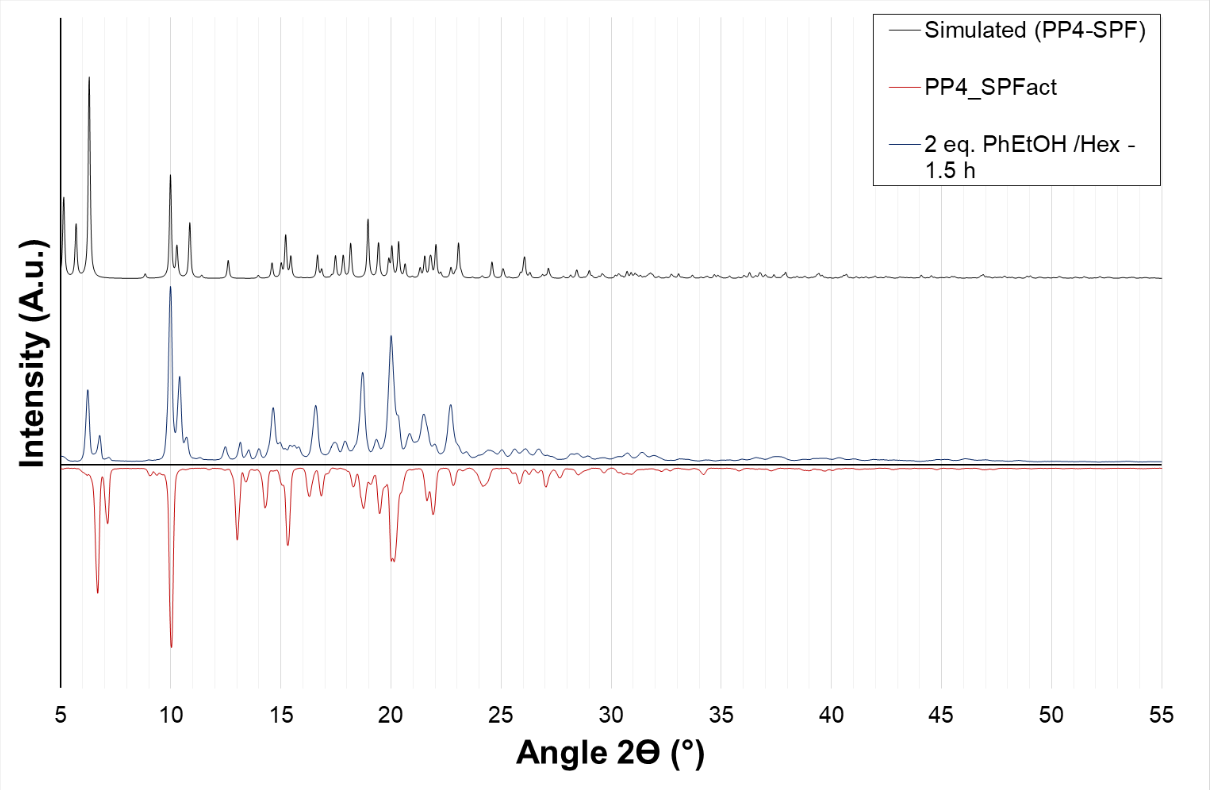

Figure S34 – PD-XRD simulated for **PP$_4$-SPF** (black, top), **PP$_4$-SPF** after activation at 45 °C under high vacuum (provided by a turbomolecular pump) (**red**), **PP$_4$-SPF$_{act}$** after soaking in a PhEtOH solution (2 eq in hexane 300 µL, HPLC grade) for 1.5 h (**blue**)

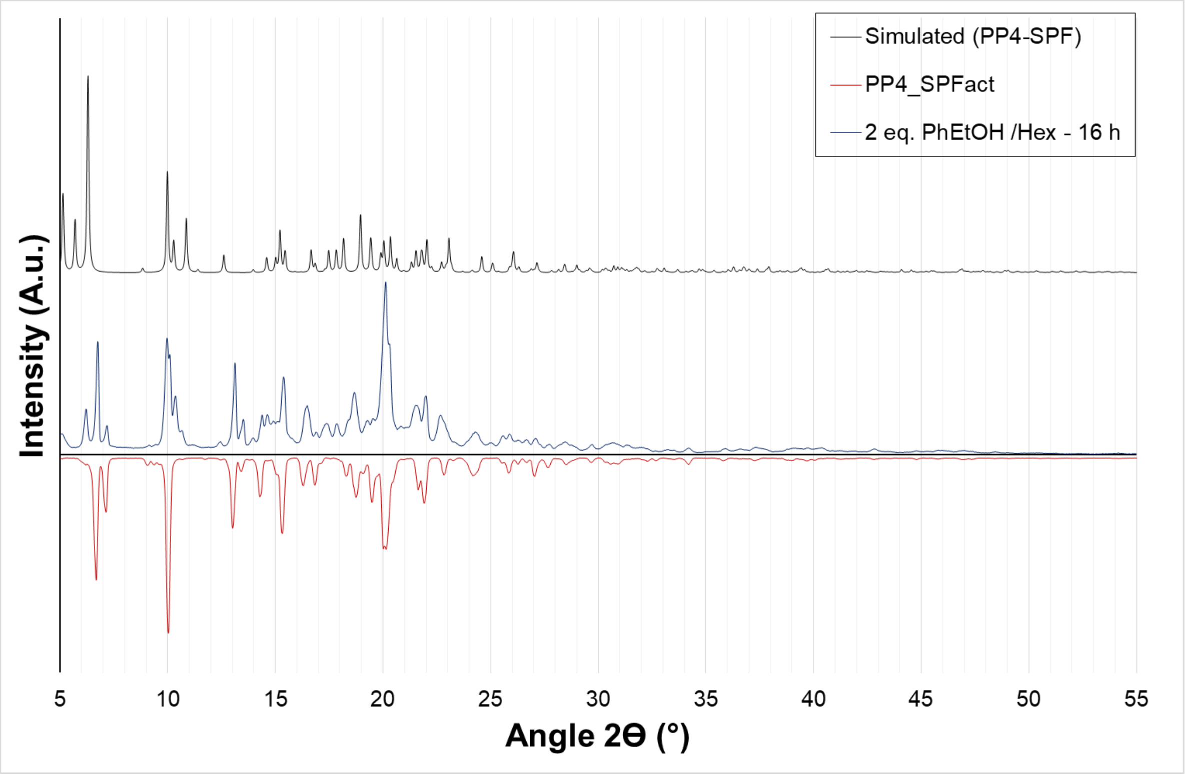

Figure S35 – PD-XRD simulated for **PP_4-SPF** (black, top), **PP_4-SPF** after activation at 45 °C under high vacuum (provided by a turbomolecular pump) (**red**), **PP_4-SPF_{act}** after soaking in a PhEtOH solution (2 eq in hexane 300 µL, HPLC grade) for 16 h (**blue**)

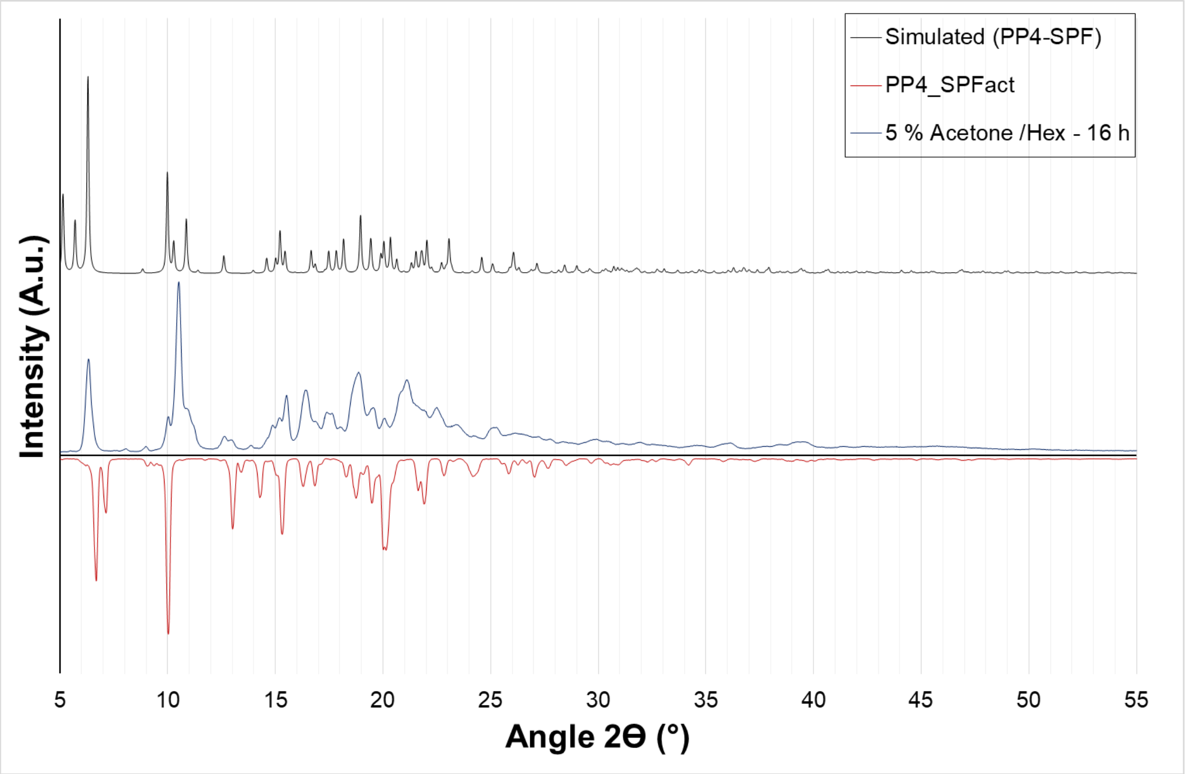

Figure S36 – PD-XRD simulated for **PP_4-SPF** (black, top), **PP_4-SPF** after activation at 45 °C under high vacuum (provided by a turbomolecular pump) (**red**), **PP_4-SPF_{act}** after soaking in a acetone solution (5 % in hexane 300 µL, HPLC grade) for 16 h (**blue**)

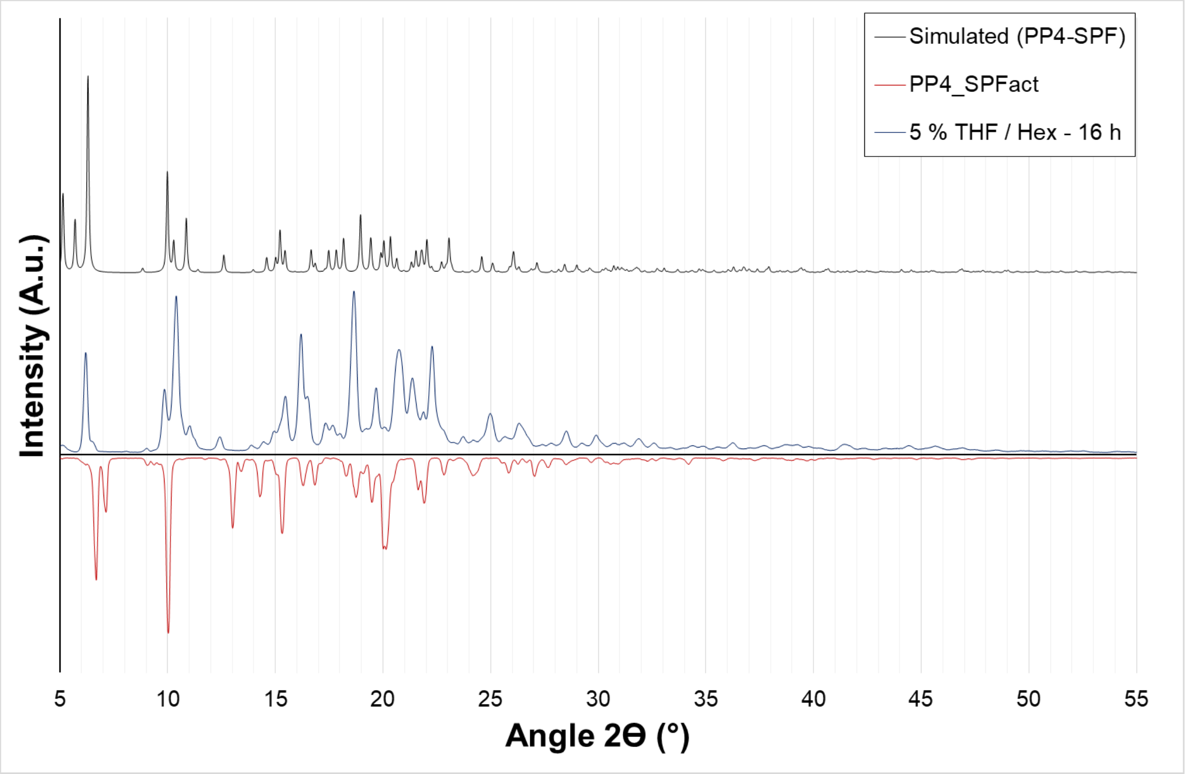

Figure S37 – PD-XRD simulated for **PP_4-SPF** (black, top), **PP_4-SPF** after activation at 45 °C under high vacuum (provided by a turbomolecular pump) (**red**), **PP_4-SPF_{act}** after soaking in a acetone solution (5 % in hexane 300 µL, HPLC grade) for 16 h (**blue**)

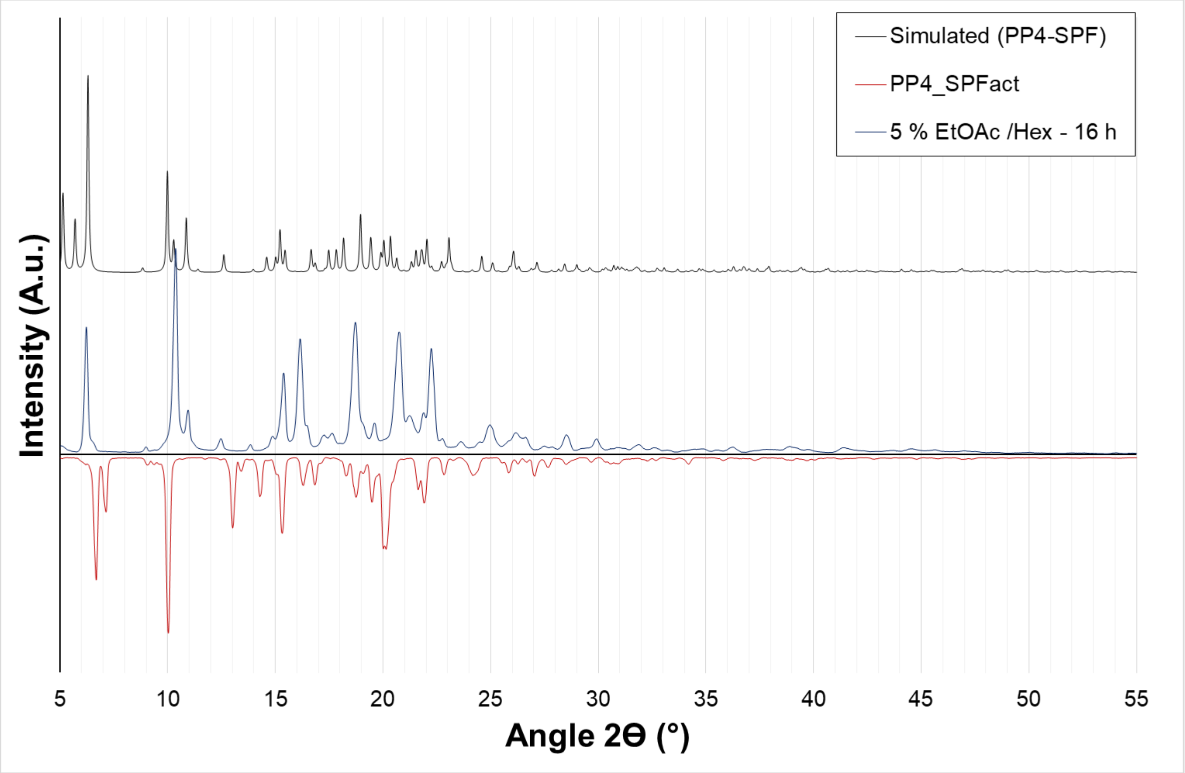

Figure S38 – PD-XRD simulated for **PP_4-SPF** (black, top), **PP_4-SPF** after activation at 45 °C under high vacuum (provided by a turbomolecular pump) (**red**), **PP_4-SPF_{act}** after soaking in a THF solution (5 % in hexane 300 µL, HPLC grade) for 16 h (**blue**)

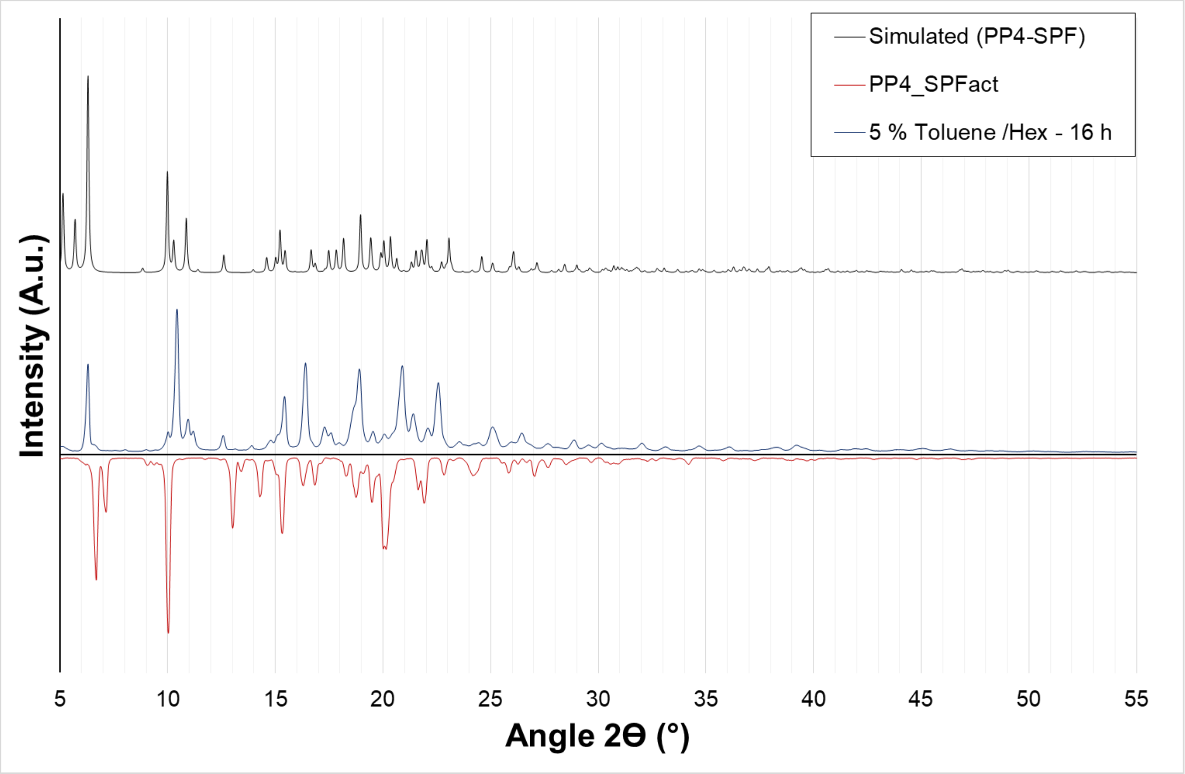

Figure S39 – PD-XRD simulated for **PP_4-SPF** (black, top), **PP_4-SPF** after activation at 45 °C under high vacuum (provided by a turbomolecular pump) (**red**), **PP_4-SPF_{act}** after soaking in a toluene solution (5 % in hexane 300 μL, HPLC grade) for 16 h (**blue**)

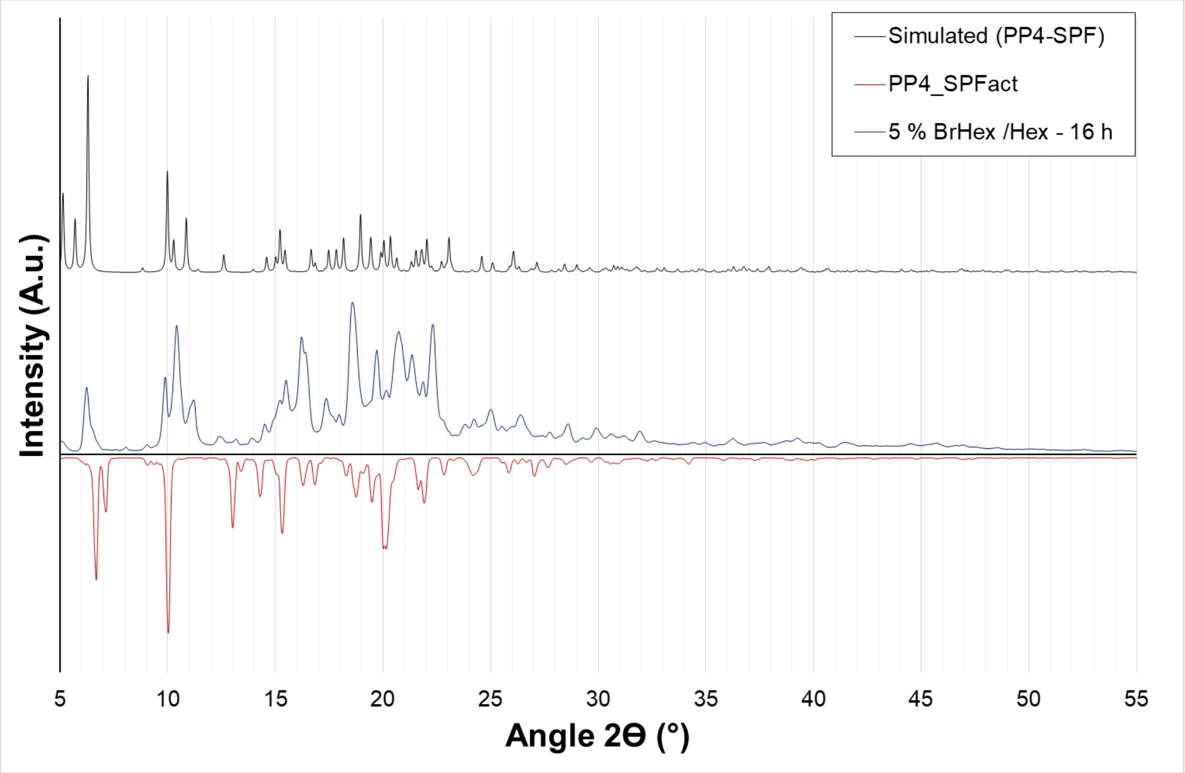

Figure S40 – PD-XRD simulated for **PP_4-SPF** (black, top), **PP_4-SPF** after activation at 45 °C under high vacuum (provided by a turbomolecular pump) (**red**), **PP_4-SPF_{act}** after soaking in a 1-bromohexane solution (5 % in hexane 300 μL, HPLC grade) for 16 h (**blue**)

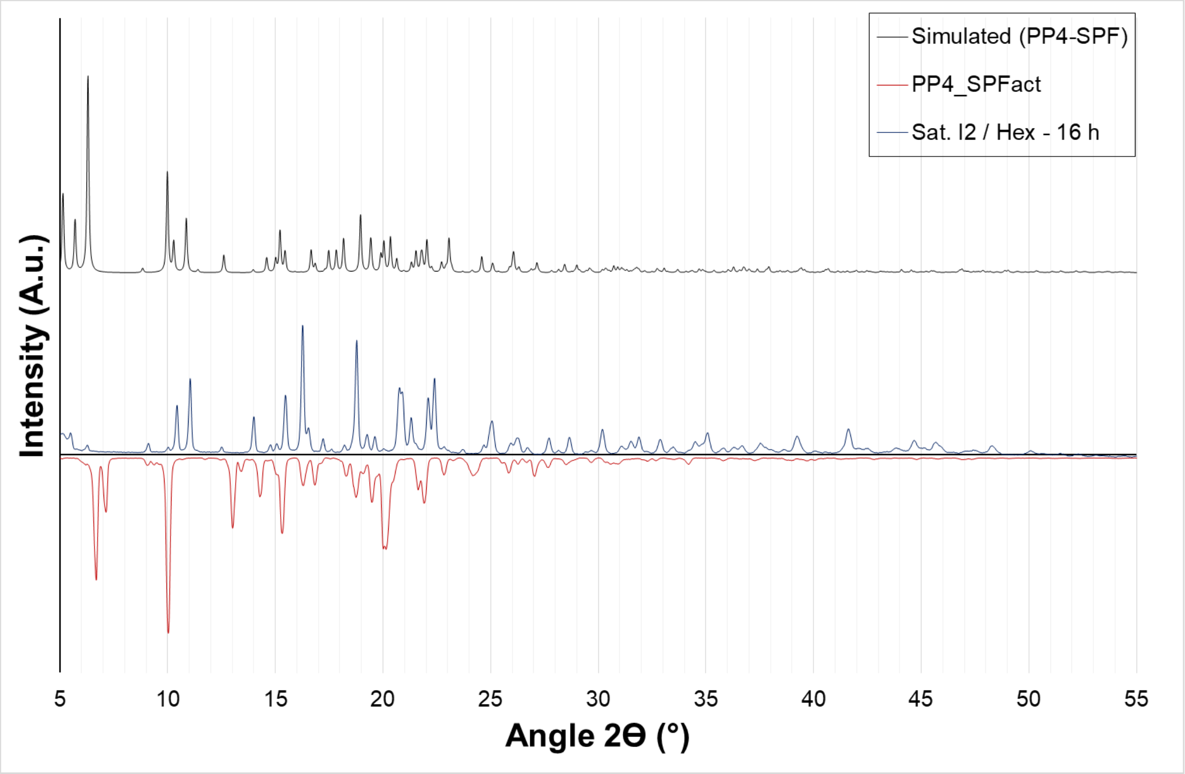

Figure S41 – PD-XRD simulated for **PP$_4$-SPF** (**black**, top), **PP$_4$-SPF** after activation at 45 °C under high vacuum (provided by a turbomolecular pump) (**red**), **PP$_4$-SPF$_{act}$** after soaking in a saturated iodine solution (hexane 300 µL, HPLC grade) for 16 h (**blue**)

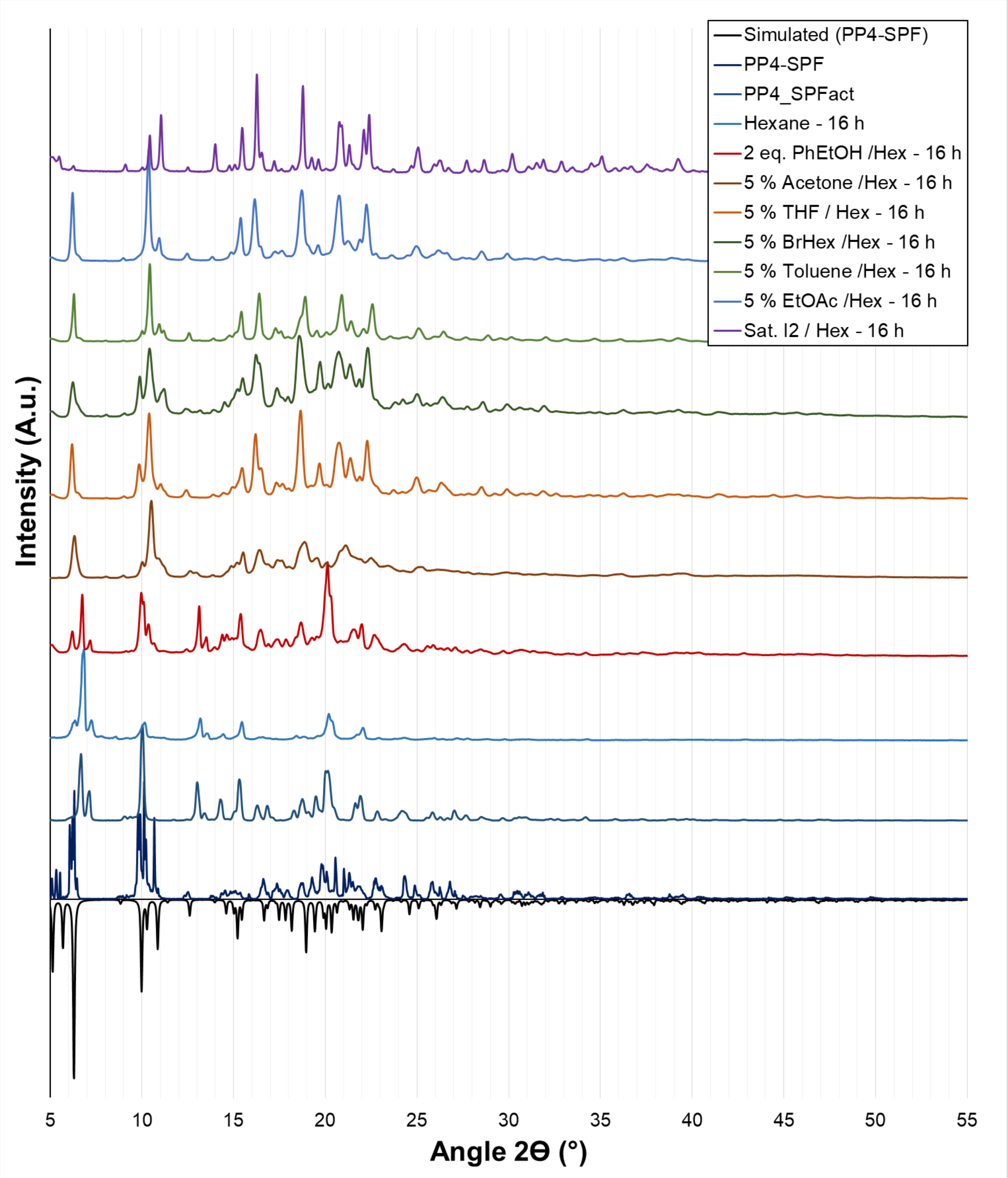

Figure S42 – Experimental PD-XRD of all studied guest molecules compared with original **PP$_4$-SPF** simulated, experimental, and after activation. Legend in order, with simulated (*bottom*) to iodine (*top*).

SI 13.5 Enantioselectivity studies of PP$_4$-SPF$_{act}$ for (±) 1-Phenylethanol

Chiral separation – Samples of **PP$_4$-SPF$_{act}$** (5-10 mg) were soaked in a premixed solution of (±) 1-phenylethanol in hexane and left for a set time without agitation. The solution was then decanted and the solid washed with hexane (300 µL x 5, HPLC grade) to remove excess PhEtOH. The samples were then dissolved in propan-2-ol (30 µL), to release encapsulated guest, before precipitating with hexane (570 µL). The dispersion was then filtered through a syringe filter (0.2 µm) before taking for

HPLC analysis. Where the SPF was used in excess, the supernatant after soaking and first hexane wash were collected, combined, filtered and taken for HPLC analysis without dilution.

HPLC analysis - The determination of enantiomeric selectivity was investigated via normal phase chiral HPLC. These were carried out with an isocratic mobile phase (A: 98 % hexane, B: 2 % propan-2-ol, 1 ml min^{-1} flow rate, 20 min runtime, 298 K) on an Agilent 1100 Series Capillary LC system with a CHIRALCEL® OD chromatographic column (*4.6 mm x 250 mm*, *10 µm*). Samples used 40 µL injections in 5 % propan-2-ol in hexane, the target analytes were monitored at 254 nm. *R*-1-phenylethanol rt = 10.45 min, *S*-1-phenylethanol rt = 13.07 min.

Table S4: HPLC Chromatogram data for *R* and *S*-PhEtOH from consecutive repeats of PP_4_SPF_{act} soaking with PhEtOH (5 % in hexane)

	Peak *rt* / min	Peak area / %	*ee* / %
Run 1	10.111	38.783	22.434
	12.547	61.217	
Run 2	10.578	36.717	26.566
	13.220	63.283	
Run 3	10.661	38.029	23.942
	13.253	61.971	
**Average**	10.450	37.843	**24.314**
	13.067	62.157	

SI 14. Computational Methods

SI 14.1 - Molecular Orbital and Electrostatic Potential Modelling

Summary: Energy decomposition analysis (EDA) of the isolated dimer system (geometry fixed to that of the **PP_4-SPF** crystal structure) was used to determine the relative breakdown of the intermolecular forces between the Fmoc groups in the crystal lattice. Calculations were run with two different functionals, BLYP-D3(BJ) and PBE-D, giving interaction energies of -44.2 and -32.4 $kJmol^{-1}$ respectively. The breakdown suggests that dispersion interactions are the predominant interaction between the Fmoc groups with smaller contributions from electrostatic and orbital interactions (and the Pauli repulsive interaction, SI 14.2, Table S4).

The Fmoc-Fmoc dimer was modelled by density functional theory at the B3LYP-D3/6-311++G (p,d) level using GAMESS version 5 (Dec 2014) with Avogadro as the GUI(v. 1.2.7).[10,11] The input geometry was that of two **PP_4** molecules associated at the terminal Fmoc ends (Figure S43)

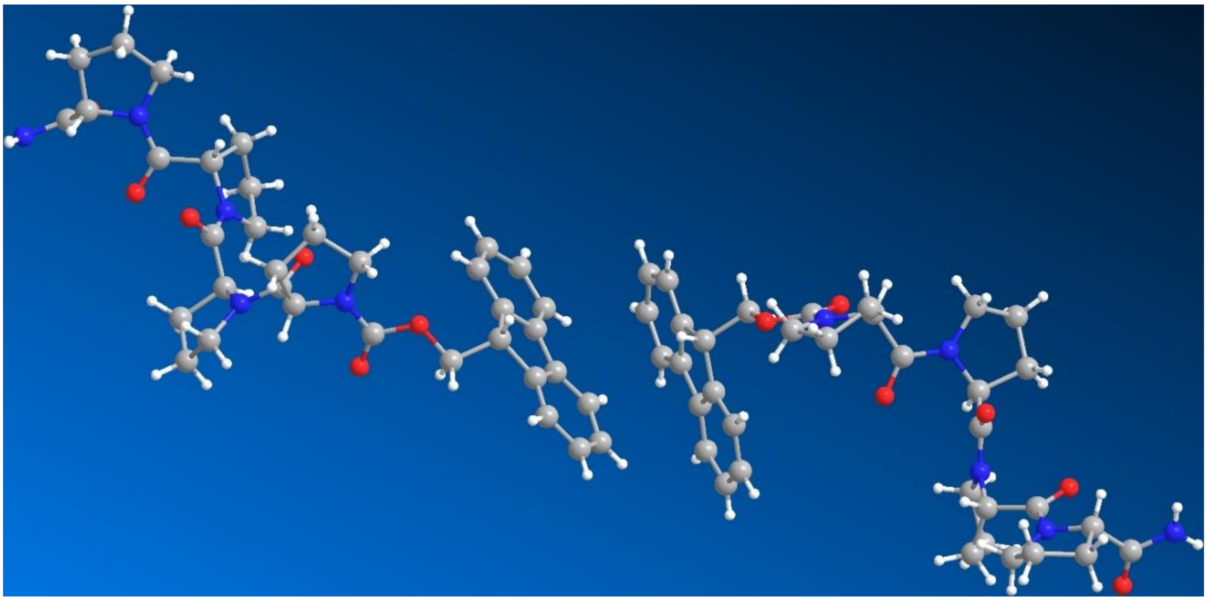

Figure S43 - Fmoc-Fmoc dimer taken from crystal structure and used as fixed geometry for subsequent molecular model calculations

The input for GAMESS is given below.

```
$BASIS GBASIS=N311 NGAUSS=6 NDFUNC=1 NPFUNC=1 DIFFSP=.TRUE. DIFFS=.TRUE. $END
 $CONTRL SCFTYP=RHF RUNTYP=ENERGY DFTTYP=B3LYP $END
 $SCF DIRSCF=.T. DAMP=.T. SOSCF=.F. $END
 $ELPOT IEPOT=1 WHERE=PDC OUTPUT=NONE $END
 $PDC PTSEL=CONNOLLY $END
 $SYSTEM MWORDS=100 $END

 $DATA
Title
C1
O   8.0   1.63200    3.86900    9.38200
O   8.0   7.19400    4.46400    14.39100
O   8.0   3.69000    2.92300    9.11200
O   8.0   8.76800    2.59100    16.50300
O   8.0   4.40400    3.62300    12.57200
O   8.0   9.52000    2.02400    19.65400
N   7.0   8.00300    3.89400    18.17000
N   7.0   3.41100    4.84200    10.29900
N   7.0   6.40200    2.50800    15.18600
N   7.0   6.49100    3.59300    11.76600
N   7.0   11.43500   3.04100    18.99200
H   1.0   11.93900   2.41000    19.28700
H   1.0   11.78500   3.72800    18.61000
C   6.0   10.10500   2.95600    19.12700
C   6.0   7.82000    3.14200    17.07400
C   6.0   6.83000    3.30100    14.20200
C   6.0   5.21300    3.99100    11.72000
C   6.0   -0.96500   1.76200    7.73800
C   6.0   -1.41200   0.56000    8.29800
C   6.0   -1.15500   0.59200    9.74000
C   6.0   2.98100    3.81000    9.56100
C   6.0   6.38900    3.01400    16.55700
H   1.0   5.94000    3.88400    16.59100
C   6.0   -1.99700   -0.41500   7.49500
H   1.0   -2.31800   -1.20600   7.86500
C   6.0   -0.53800   1.81200    10.05400
C   6.0   6.97100    4.52500    18.99400
H   1.0   6.64500    3.91600    19.67700
H   1.0   6.22300    4.82500    18.45400
C   6.0   -0.35800   2.64600    8.81600
H   1.0   -0.84800   3.49100    8.89100
C   6.0   -1.42500   -0.35300   10.73100
H   1.0   -1.84400   -1.15600   10.52100
C   6.0   9.35600    4.19700    18.65300
H   1.0   9.86900    4.66200    17.95900
C   6.0   6.92800    2.67000    12.82200
H   1.0   6.40000    1.84500    12.79000
C   6.0   -1.61800   1.00100    5.56500
```

H	1.0	-1.67700	1.13500	4.64700
C	6.0	4.82300	4.96500	10.60900
H	1.0	5.37300	4.82600	9.81000
C	6.0	-2.09100	-0.17800	6.12400
H	1.0	-2.47700	-0.82100	5.57500
C	6.0	1.10700	2.89800	8.47100
H	1.0	1.61100	2.07100	8.54100
H	1.0	1.18200	3.22400	7.56000
C	6.0	5.84600	1.15000	15.06700
H	1.0	6.42500	0.58000	14.53800
H	1.0	4.96300	1.16600	14.66600
C	6.0	5.56400	1.96400	17.31300
H	1.0	5.87900	1.86600	18.22600
H	1.0	4.62400	2.20500	17.32900
C	6.0	-1.05200	1.98600	6.37600
H	1.0	-0.73900	2.78000	6.00500
C	6.0	-0.16200	2.10400	11.36300
H	1.0	0.25100	2.91000	11.57400
C	6.0	7.71200	5.70100	19.61300
H	1.0	7.74000	6.45600	19.00500
H	1.0	7.29800	5.97800	20.44500
C	6.0	-1.05800	-0.06800	12.04400
H	1.0	-1.23500	-0.68500	12.71700
C	6.0	-0.42300	1.13900	12.35500
H	1.0	-0.16900	1.30800	13.23400
C	6.0	5.78500	0.68000	16.51900
H	1.0	5.05100	0.05900	16.65200
H	1.0	6.61400	0.24900	16.78000
C	6.0	4.92100	6.41400	11.10000
H	1.0	5.63900	6.51600	11.74300
H	1.0	5.07300	7.02100	10.35800
C	6.0	9.10200	5.13400	19.85300
H	1.0	9.13400	4.64200	20.68700
H	1.0	9.76200	5.84400	19.88100
C	6.0	3.57100	6.67400	11.74600
H	1.0	3.37400	7.62300	11.76900
H	1.0	3.54600	6.32300	12.65000
C	6.0	7.56900	3.88300	10.82600
H	1.0	8.02400	4.70600	11.06500
H	1.0	7.22800	3.96200	9.92100
C	6.0	2.58800	5.92600	10.83500
H	1.0	1.83500	5.58000	11.34000
H	1.0	2.25800	6.50100	10.12700
C	6.0	8.39400	2.37700	12.44000
H	1.0	9.00100	2.93300	12.95300
H	1.0	8.61200	1.44500	12.59900
C	6.0	8.48300	2.70700	10.95500
H	1.0	9.38900	2.93800	10.70000

H	1.0	8.17700	1.96400	10.41200
O	8.0	-7.60700	7.00700	7.82100
O	8.0	-13.16900	7.60300	2.81200
O	8.0	-9.66500	6.06200	8.09200
O	8.0	-14.74200	5.73000	0.70000
O	8.0	-10.37800	6.76100	4.63100
O	8.0	-15.49400	5.16300	-2.45100
N	7.0	-13.97700	7.03300	-0.96600
N	7.0	-9.38600	7.98100	6.90400
N	7.0	-12.37700	5.64700	2.01700
N	7.0	-12.46500	6.73100	5.43700
N	7.0	-17.40900	6.18000	-1.78900
H	1.0	-17.91300	5.54900	-2.08400
H	1.0	-17.76000	6.86600	-1.40600
C	6.0	-16.08000	6.09500	-1.92400
C	6.0	-13.79500	6.28100	0.12900
C	6.0	-12.80400	6.43900	3.00100
C	6.0	-11.18700	7.13000	5.48300
C	6.0	-5.01000	4.90100	9.46500
C	6.0	-4.56200	3.69900	8.90500
C	6.0	-4.82000	3.73100	7.46300
C	6.0	-8.95600	6.94800	7.64300
C	6.0	-12.36400	6.15200	0.64600
H	1.0	-11.91500	7.02300	0.61200
C	6.0	-3.97700	2.72400	9.70800
H	1.0	-3.65700	1.93300	9.33800
C	6.0	-5.43700	4.95000	7.15000
C	6.0	-12.94600	7.66300	-1.79100
H	1.0	-12.62000	7.05500	-2.47300
H	1.0	-12.19800	7.96400	-1.25100
C	6.0	-5.61700	5.78500	8.38700
H	1.0	-5.12600	6.63000	8.31300
C	6.0	-4.54900	2.78600	6.47200
H	1.0	-4.13100	1.98300	6.68300
C	6.0	-15.33000	7.33600	-1.44900
H	1.0	-15.84300	7.80100	-0.75600
C	6.0	-12.90300	5.80800	4.38200
H	1.0	-12.37400	4.98400	4.41400
C	6.0	-4.35700	4.14000	11.63800
H	1.0	-4.29700	4.27300	12.55700
C	6.0	-10.79800	8.10400	6.59400
H	1.0	-11.34800	7.96400	7.39300
C	6.0	-3.88400	2.96100	11.07900
H	1.0	-3.49800	2.31800	11.62800
C	6.0	-7.08100	6.03600	8.73200
H	1.0	-7.58600	5.21000	8.66200
H	1.0	-7.15700	6.36200	9.64300
C	6.0	-11.82100	4.28900	2.13700

H	1.0	-12.40000	3.71800	2.66600
H	1.0	-10.93800	4.30500	2.53800
C	6.0	-11.53800	5.10200	-0.11000
H	1.0	-11.85300	5.00400	-1.02300
H	1.0	-10.59900	5.34400	-0.12600
C	6.0	-4.92300	5.12400	10.82800
H	1.0	-5.23600	5.91800	11.19800
C	6.0	-5.81300	5.24200	5.84000
H	1.0	-6.22500	6.04900	5.62900
C	6.0	-13.68600	8.83900	-2.41000
H	1.0	-13.71500	9.59500	-1.80200
H	1.0	-13.27200	9.11700	-3.24200
C	6.0	-4.91600	3.07000	5.15900
H	1.0	-4.73900	2.45400	4.48600
C	6.0	-5.55200	4.27800	4.84800
H	1.0	-5.80500	4.44700	3.96900
C	6.0	-11.76000	3.81900	0.68500
H	1.0	-11.02600	3.19800	0.55100
H	1.0	-12.58800	3.38800	0.42400
C	6.0	-10.89500	9.55300	6.10400
H	1.0	-11.61400	9.65400	5.46100
H	1.0	-11.04700	10.16000	6.84500
C	6.0	-15.07600	8.27300	-2.64900
H	1.0	-15.10800	7.78000	-3.48400
H	1.0	-15.73700	8.98300	-2.67800
C	6.0	-9.54600	9.81200	5.45700
H	1.0	-9.34800	10.76100	5.43400
H	1.0	-9.52100	9.46100	4.55300
C	6.0	-13.54300	7.02200	6.37700
H	1.0	-13.99900	7.84400	6.13900
H	1.0	-13.20300	7.10000	7.28200
C	6.0	-8.56200	9.06500	6.36900
H	1.0	-7.81000	8.71800	5.86300
H	1.0	-8.23200	9.64000	7.07600
C	6.0	-14.36900	5.51600	4.76400
H	1.0	-14.97600	6.07200	4.25000
H	1.0	-14.58700	4.58400	4.60500
C	6.0	-14.45700	5.84600	6.24800
H	1.0	-15.36300	6.07600	6.50300
H	1.0	-14.15100	5.10300	6.79100

$E

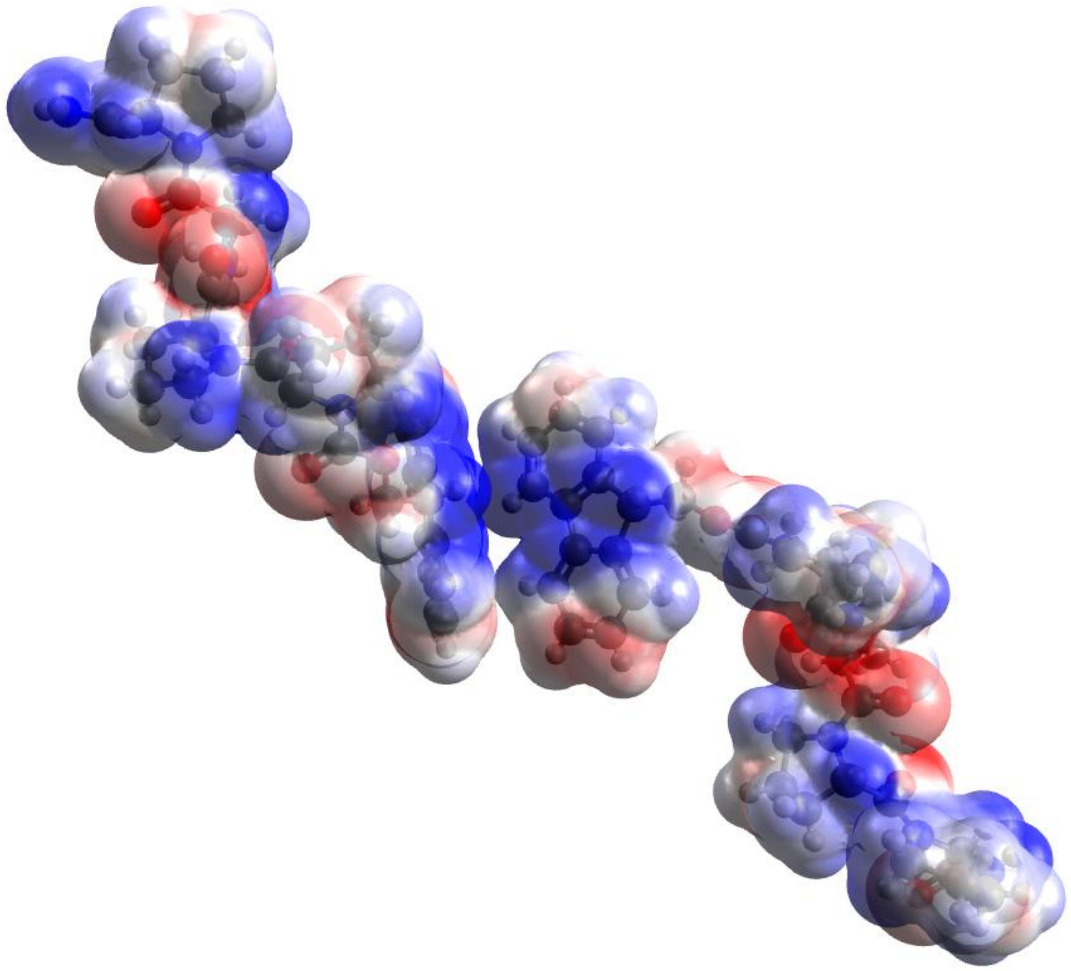

Figure S44 - Electrostatic potential map (blue – positive, red – negative) calculated at B3LYP-D3/6-311-G++(p,d) level of theory

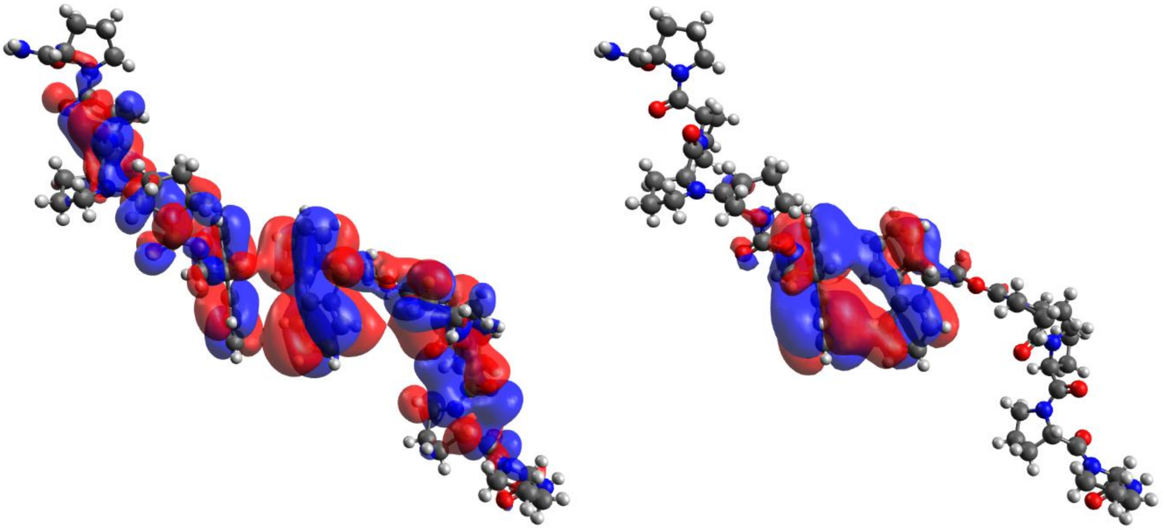

Figure S45 - HOMO-6 (left) and HOMO-19 (right) highlighting orbital interactions between discrete molecules, calculated at B3LYP-D3/6-311-G++(p,d) level of theory

SI 14.2 Energy Decomposition Analysis

EDA was conducted on the Fmoc-Fmoc dimer obtained from the crystal geometry (*Figure S43*) using the Amsterdam Density Functional (Amsterdam Modelling Suite) with the BLYP-D(BJ) and PBE-D functionals (frozen cores). [12]

Table S5. Energy components of Fmoc-Fmoc interactions from EDA (kJ mol^{-1}).

**Func.**	E_{elstat}	E_{Pauli}	E_{orb}	E_{disp}	E_{total}
BLYP-D3(BJ)	-15.5	32.6	-12.9	-48.3	-44.2
PBE-D	-13.7	17.9	-8.7	-27.9	-32.4

Model parameters were as follows.

```
DENSITY FUNCTIONAL POTENTIAL (scf)
    LDA:                               Exchange only
== Not Default ==
    Gradient Corrections:              Becke88 LYP
== Not Default ==

 SPIN  (restricted / unrestr.)
    Molecule:                          Restricted
    Fragments:                         Restricted

 OTHER ASPECTS
    Relativistic Corrections:          scalar (ZORA,MAPA)

    Nuclear Charge Density Model:      Point Charge Nuclei
    Core Treatment:                    Frozen Orbital(s)

    Hyperfine or Zeeman Interaction:   ---

 Settings for Grimme D3 dispersion correction

      damping                          BJ
      s6                            1.000
      s8                            2.700
      a1                            0.430
      a2                            4.236

 Other (technical) parameters

      alpha                        14.000
      version                           4



DENSITY FUNCTIONAL POTENTIAL (scf)
    LDA:                               PW92
== Not Default ==
    Gradient Corrections:              PBEc PBEx
== Not Default ==
```

```
SPIN  (restricted / unrestr.)
   Molecule:                          Restricted
   Fragments:                         Restricted

OTHER ASPECTS
   Relativistic Corrections:          scalar (ZORA,MAPA)

   Nuclear Charge Density Model:      Point Charge Nuclei
   Core Treatment:                    Frozen Orbital(s)

   Hyperfine or Zeeman Interaction:   ---

Settings for Grimme dispersion correction
  use heavy dispersion       T
  scaling of radii              1.100
  alpha (scaling function)     20.000
  cut off distance            100.000
  overall factor                0.750
```

Acknowledgements

A.P., K.S. acknowledge financial support from the EPSRC (Grant number EP/T016140/1). D.F.B. acknowledges support from the University of Kent (Vice Chancellor's Fellowship). G.T. acknowledges financial support from the Royal Society of Chemistry (Grant Number R19-2762), A.P. acknowledges financial support from the Royal Society of Chemistry (Grant E21-9299054940). C.S.H. acknowledges the School of Chemical and Physical Sciences, Keele University for financial support.

Author Contributions: Experiments were planned and executed by D.F.B., K.S, E.J.F and A.P. X-Ray diffraction data were collected and analysed by G.T, D.F.B, C.S.H and H.J.S. NMR, LC-MS were performed by D.F.B, K.S. and A.P. HPLC, Circular dichroism and STA were performed by D.F.B. Gas adsorption studies were carried out by C.S.H. Computational calculations were performed by S.J.H. All authors contributed to data interpretation and writing of the manuscript. All authors have given approval to the final version of the manuscript.

References

[1] D. RK, K. TA, *Biopolymers* **1991**, *31*, 1747–1761.

[2] O. V. Dolomanov, L. J. Bourhis, R. J. Gildea, J. A. K. Howard, H. Puschmann, *J. Appl. Crystallogr.* **2009**, *42*, 339–341.

[3] G. M. Sheldrick, *Acta Crystallogr. Sect. A Found. Crystallogr.* **2008**, *64*, 112–122.

[4] G. M. Sheldrick, *Acta Crystallogr. Sect. A Found. Crystallogr.* **2015**, *71*, 3–8.

[5] G. M. Sheldrick, *Acta Crystallogr. Sect. C Struct. Chem.* **2014**, *71*, 3–8.

[6] H. K. Ganguly, G. Basu, *Biophys. Rev.* **2020**, *12*, 25–39.

[7] P. Wilhelm, B. Lewandowski, N. Trapp, H. Wennemers, *J. Am. Chem. Soc.* **2014**, *136*, 15829–15832.

[8] Bruker APEX-3, **2016**, Bruker-AXS Inc., Madison, WI.

[9] SADABS 2016/2, **2016**, Bruker-AXS Inc., Madison, WI.

[10] M. W. Schmidt, K. K. Baldridge, J. A. Boatz, S. T. Elbert, M. S. Gordon, J. H. Jensen, S. Koseki, N. Matsunaga, K. A. Nguyen, S. Su, T. L. Windus, M. Dupuis, J. A. Montgomery, *J. Comput. Chem.* **1993**, *14*, 1347–1363.

[11] M. D. Hanwell, D. E. Curtis, D. C. Lonie, T. Vandermeerschd, E. Zurek, G. R. Hutchison, *J. Cheminform.* **2012**, *4*, 1–17.

[12] G. te Velde, F. M. Bickelhaupt, E. J. Baerends, C. Fonseca Guerra, S. J. A. van Gisbergen, J. G. Snijders, T. Ziegler, *J. Comput. Chem.* **2001**, *22*, 931–967.
